# GOLGA7 is essential for NRAS trafficking from the Golgi to the plasma membrane but not for its palmitoylation

**DOI:** 10.1186/s12964-024-01498-w

**Published:** 2024-02-05

**Authors:** Chenxuan Liu, Bo Jiao, Peihong Wang, Baoyuan Zhang, Jiaming Gao, Donghe Li, Xi Xie, Yunying Yao, Lei Yan, Zhenghong Qin, Ping Liu, Ruibao Ren

**Affiliations:** 1 Shanghai Institute of Hematology, State Key Laboratory for Medical Genomics, Collaborative Innovation Center of Hematology, National Research Center for Translational Medicine at Shanghai, Ruijin Hospital, Shanghai Jiao Tong University School of Medicine, Shanghai, China; 2 International Center for Aging and Cancer, Hainan Medical College, Haikou, Hainan Province China; 3 Laboratory of Aging and Nervous Diseases, Department of Pharmacology, College of Pharmaceutical Science, Soochow University, Suzhou, 215123 China

## Abstract

*NRAS* mutations are most frequently observed in hematological malignancies and are also common in some solid tumors such as melanoma and colon cancer. Despite its pivotal role in oncogenesis, no effective therapies targeting NRAS has been developed. Targeting NRAS localization to the plasma membrane (PM) is a promising strategy for cancer therapy, as its signaling requires PM localization. However, the process governing NRAS translocation from the Golgi apparatus to the PM after lipid modification remains elusive. This study identifies GOLGA7 as a crucial factor controlling NRAS’ PM translocation, demonstrating that its depletion blocks NRAS, but not HRAS, KRAS4A and KRAS4B, translocating to PM. GOLGA7 is known to stabilize the palmitoyltransferase ZDHHC9 for NRAS and HRAS palmitoylation, but we found that GOLGA7 depletion does not affect NRAS’ palmitoylation level. Further studies show that loss of GOLGA7 disrupts NRAS anterograde trafficking, leading to its *cis*-Golgi accumulation. Remarkably, depleting GOLGA7 effectively inhibits cell proliferation in multiple NRAS-mutant cancer cell lines and attenuates NRAS^G12D^-induced oncogenic transformation in vivo. These findings elucidate a specific intracellular trafficking route for NRAS under GOLGA7 regulation, highlighting GOLGA7 as a promising therapeutic target for NRAS-driven cancers.

**Supplementary Information:**

The online version contains supplementary material available at 10.1186/s12964-024-01498-w.

## Introduction

RAS proteins are small GTPases that act as binary molecular switches, transducing extracellular signals from activated receptors through various signaling pathways. These pathways trigger cellular processes relevant to proliferation and survival [1, 2]. Constitutively active *RAS* mutations are associated with up to approximately 30% of all human cancers, including both solid tumors and hematologic malignancies, with specific RAS isoforms preferentially associated with specific cancer types [3].

The three human *RAS* genes encode four highly homologous proteins: HRAS, NRAS, KRAS4A, and KRAS4B (KRAS). The latter two are splice variants of a single locus. RAS proteins localize to the inner leaflet of the plasma membrane (PM) by two motifs contained in their C-terminal hypervariable domain [3]. The first motif, which is common to all RAS proteins, is a C-terminal CAAX motif that undergoes post-translational modifications, generating a cysteine farnesyl carboxyl-methyl ester [4]. The second motif varies between RAS isoforms, consisting of a polybasic domain for KRAS4B and either one or two palmitoylation sites for NRAS, HRAS, and KRAS4A [5–8]. The trafficking of RAS to the PM depends on the nature of the second signal. KRAS4B reaches the PM by an unknown mechanism that independent of vesicular transport, whereas palmitoylated RAS isoforms are delivered from the Golgi to the PM as part of the secretory pathway that has yet to be fully unraveled [9–12]. Although novel inhibitors of KRAS4B^G12C^ have displayed promising results, direct targeting of other mutations and RAS isoforms remains challenging [13, 14]. Preventing the localization of RAS to the PM completely annuls RAS biological activity [5, 6, 15]. Thus, interfering with RAS-PM interactions has therapeutic potentials.

NRAS is the most frequently mutated RAS isoform in melanoma and many hematologic malignancies, with a prevalence of approximately 20% to 40% in acute myeloid leukemia (AML), myelodysplastic syndrome (MDS) and juvenile chronic myelomonocytic leukemias [16–18]. Studies have shown that oncogenic NRAS requires palmitoylation for its PM localization and activation of downstream signaling, both in vitro and in vivo [6, 19, 20]. The protein palmitoyltransferase encoded by the *ZDHHC9* gene, in conjunction with its accessory protein GOLGA7 (Golgin Subfamily A Number 7, also known as GCP16), catalyzes the palmitoylation of NRAS and HRAS in vitro [21]. However, previous research using mouse models has demonstrated that inactivation of ZDHHC9 only partially mitigates the leukemogenic potential of oncogenic NRAS [22]. This is likely due to the redundancy among ZDHHC family proteins [23, 24].

GOLGA7 is a reportedly Golgi-resident peripheral membrane protein with two palmitoylation sites at cysteine 69 and 72, respectively [25]. Recent studies have suggested that GOLGA7 acts as an accessory protein for multiple ZDHHC family proteins, including ZDHHC9, ZDHHC14 and ZDHHC18 [21, 24, 26]. GOLGA7 also plays a regulatory role in PM-localized ZDHHC5 or ZDHHC8, which have been linked to retrograde flux from the PM or endosomes in a cell-context dependent manner [26]. Furthermore, GOLGA7 is involved in protein transport from the Golgi to the PM, as overexpression of wild type GOLGA7 retains VSV-G proteins in the Golgi [15]. In this study, we demonstrate that depletion of GOLGA7 specifically inhibits the PM localization of NRAS and traps NRAS in the *cis*-Golgi without affecting its palmitoylation level. Additionally, we found that GOLGA7 is essential for the proliferation and signaling of cancer cells harboring oncogenic NRAS.

## Methods

### CRISPR/Cas9 gene editing

Genomic disruption of GOLGA7 was performed by infecting HeLa cells and Ba/F3 cells with lentivirus generated by transfecting HEK293 cells with pLentiCRISPR v2 with two sgRNAs targeting GOLGA7 (GOLGA7-sg1,5’- ATGAGGCCGCAGCAGGCGC-3’ or GOLGA7-sg2, 5’- CAGGCGCCGGTGTCCGGAA -3’), and Cas9 using Lipofectamine 3000 (Invitrogen, Life Technologies). Cells were selected with 1.5 μg/ml puromycin 7 d after infection and used immediately for experiments.

### Cell culture and transfection

HeLa, 293 T, Hep-G2, T24, SK-MEL-2 and SK-MEL-30 cells were from ATCC. These cells were cultured in DMEM supplemented with 10% FBS. OCI-AML3, HL-60, THP-1 and NB4 (all from ATCC) cells were cultured in RPMI-1640 medium supplemented with 10% FBS. The growth factor dependent Ba/F3 cells were cultured in RPMI 1640 medium including 10% FBS and supplemented with recombinant IL-3 (Roche) at the final concentration of 1 ng/mL. All cells used in our study were maintained at 37 °C in 5% CO2 incubator and tested negative for mycoplasma. Transfection was performed using Lipofectamine 2000 (Invitrogen) or DharmaFECT kb DNA transfection reagent (HORIZON) according to the manufacturer’s instructions.

### Immunofluorescence imaging

For immunofluorescence microscopy, cells were transduced with plasmids containing the required fluorescent protein-tagged construct 1 day before imaging. Cells were then fixed with 4% paraformaldehyde for 15 min at room temperature, permeabilized with Triton™ X-100 and blocked with PBS + 10% goat serum for 1 h. Cells were then incubated with indicated primary antibodies for 2 h at room temperature followed by matching secondary antibodies for 1 h at room temperature and counterstained with Hoechst33342. Cell imaging was performed using an LSM 880 inverted confocal microscope running Zen Blue software (ZEISS) with a 63 × 1.4 NA objective. Colocalization analysis using Pearson’s correlation coefficiency test was performed by the Coloc2 plugin of ImageJ.

### Animal study

In the Ba/F3 mouse model, *Golga7*-knockout Ba/F3 cells generated by CRISPR/Cas9 were transfected and selected to stablely expess GFP-tagged NRAS^G12D^ or KRAS4B^G12D^ (*Golga7*-knockout Ba/F3-N or Ba/F3-K cells) at the presence of IL-3. Lethally-irradiated (7 Gy) six-week-old female BALB/c mice (Charles River, Beijing, China) were randomized into four groups (Ba/F3-N and Ba/F3-K expressing or depletion of GOLGA7, respectively, *n* = 10 for each group). For each recipient mouse, 5 × 10^4^ Ba/F3 cells along with 1 × 10^6^ bone marrow cells from healthy donor mice were injected via the tail vein at Day 0 of the experiment, immediately after IL-3 deprivation. Peripheral blood (PB) was collected to assess the leukemia burden at Day 10 by detecting the percentage of GFP-positive cells. GraphPad Prism was used to plot Kaplan–Meier survival curves.

### Subcellular fractionation assay

Subcellular Protein fractions (plasma membrane, organelle and cytosol) were separated using the Minute™ plasma membrane isolation kit (Invent Biotechnologies) according to the manufacturer’s instructions.

### ts045-VSVG-EGFP trafficking assay

Cells were transfected with a plasmid containing the ts045-VSVG–EGFP construct, incubated at 40 °C for 20 h and then transferred to 32 °C and left for fixed intervals of 0, 30, 60, 90 or 120 min. At each time point indicated cells were fixed, permeabilized and counterstained with the nuclear marker Hoechst33342 before imaging.

### Western blot analysis

Cells were lysed at 1 × 10^7^ cells per ml in 1 × sodium dodecyl sulfate (SDS) sample loading buffer, and were cleared by centrifugation at 12 000 × g for 10 min. Equal amounts of protein samples were loaded to 8–15% Bis–Tris gels, transferred onto nitrocellulose membranes, and then blotted with specific primary and secondary antibodies. Luminescence signals were detected with Immobilon Western HRP Substrate (Millipore, Darmstadt, Germany) and blots were imaged by a ChemiDoc MP imaging system (Bio-Rad). List of antibodies and uncropped western blots are presented in Supplementary files.

### Fluorescence recovery after photobleaching and photoactivation assay

FRAP was performed on genome-edited HeLa cells transfected with GFP-NRAS^G12D^. STED confocoal microscopy (Leica TCS SP8) was used to analyze live cells at 37 °C and 5% CO2 in 10% FBS containing DMEM media. Circular regions of interest (ROI) were drawn around the regions of the Golgi, which were subsequently bleached using 100% 488 nm laser power for 3 iterations after 1 initial scan. Serial 512 × 512-pixel resolution micrographs were collected using a minimal pixel dwell time and a maximum scan speed every 1 s for 3 min. The ROI fluorescence intensities were corrected and normalized at each time point, and the fluorescence recovery was graphed as the fraction of initial fluorescence after bleaching. Curves are acquired from mean recovery values ± SD. All data were acquired using identical bleach and acquisition settings for all cells tested (*n* = 10 per condition).

Kinetic tracing of photoactivated GFP-NRAS^G12D^ was performed by photoactivating circular ROI indicating the Golgi apparatus in cells expressing G(PA)C-NRAS^G12D^ and imaged using 488 nm laser at 5% power, 559 nm laser at 5% power and a 4 μs/pixel dwell time. Photoactivation was achieved using a 405 nm laser at 7% power, a 40 μs/pixel dwell time, and a 200-ms activation pulse. Acquired fluorescence intensities analysis again included correction and normalization. Data points reflect the mean fluorescence intensity ± SD over time relative to the maximum fluorescence generated immediately after activation for cells examined (*n* = 10 per condition).

### Acyl-RAC assay

Detection of S-palmitoylated GFP-NRAS^G12D^ proteins was conducted using the commercially available CAPTUREome™ S-Palmitoylated Protein Kit (Badrilla, K010-311). The acyl-RAC assay includes (1) blocking of free thiols, (2) cleavage of thioester linkages to release the palmitate group, (3) capture of nascent thiols on the resins and (4) elution of captured proteins. Briefly, proteins were extracted from collected cells, diluted to the same concentration and then incubated at 40 °C for 4 h in the blocking buffer for blocking of free thiols. Proteins were precipitated, washed and re-dissolved in 300 μL binding buffer. After removing insoluble debris by centrifuging, 20 μL of each supernatant was saved as the “total input (IF)”. The remaining lysates were equally divided to two groups. Thioester cleavage reagent was added to the “Palm” group and preserve reagent to the “Neg” group as negative control. The capture resin slurry was equally added to each group and incubated to capture nascent thiols. Resins were then centrifuged and washed 5 times. 50 µL of supernatant was saved as “Supernatant”, referring to unbound fraction in the “Palm” group or the “Neg” group. After removing the final wash, captured proteins were eluted from the resin and saved as “IP”, referring to captured fraction in the “Palm” group or the “Neg” group. Saved fractions were boiled with 2 × Laemmli Sample Buffer for further western blotting.

### Statistical analysis

To compare differences observed in mouse survival, *p* values were calculated with the log-rank test, with a threshold for statistical significance of *p* < 0.05. Comparison between different groups were carried out using a two-tailed Student’s t-test (GraphPad Prism® Software). Error bars represent the standard deviation of the mean (± SD). A *P*-value < 0.05 was considered to be statistically significant.

## Results

### GOLGA7 is required for NRAS PM localization

**Fig. 1:**
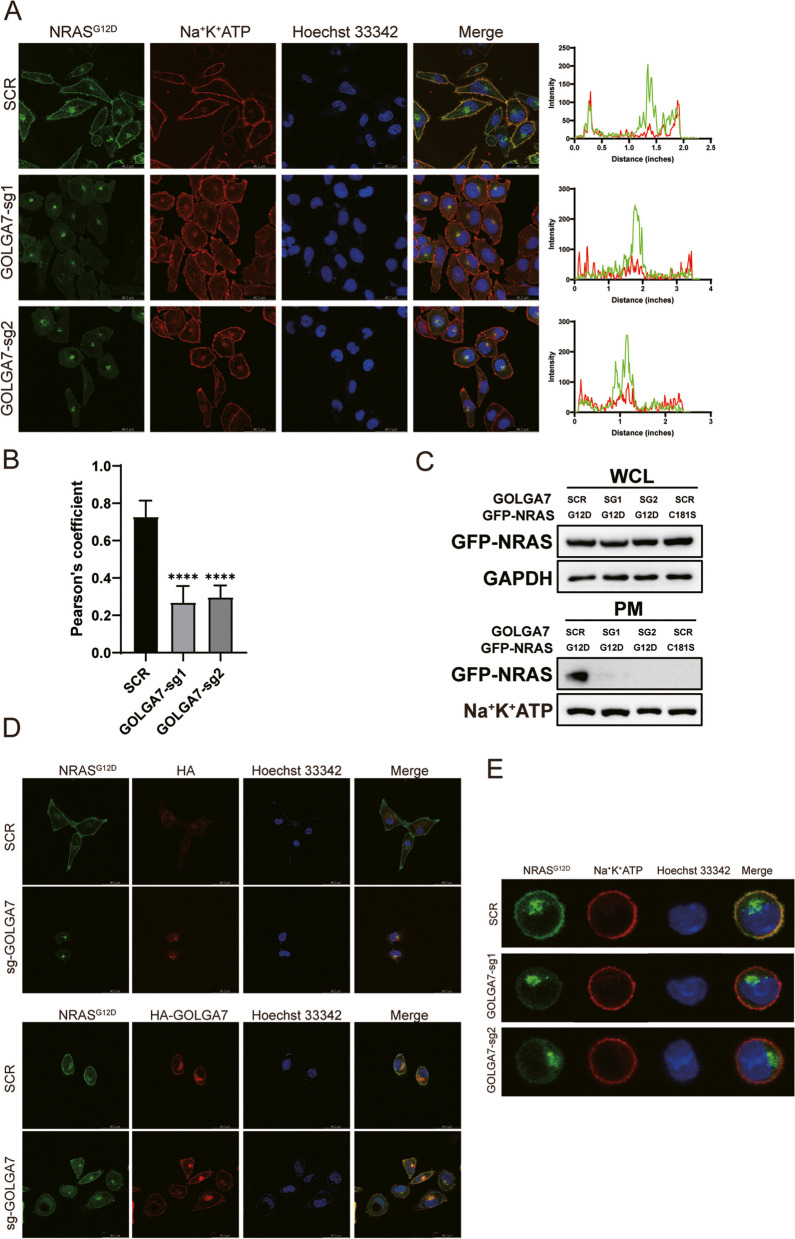
GOLGA7 is required for the PM localization of NRAS (**A**) GFP-NRAS^G12D^ (green) was visualized by confocal microscopy in HeLa cells ± sgGOLGA7, the plasma membrane(red) is indicated with anti-Na^+^K^+^ATPase, the nuclei(blue) is indicated with staining of Hoechst 33,342. The intensity profiles of GFP-NRAS^G12D^ / the plasma membrane were plotted in the right panel. Scale bar, 42.6 µm. **B** Statistical quantification of the colocalizations (Pearson’s coefficient) of GFP-NRAS^G12D^/ the plasma membrane. Values are the means ± SD from *n* = 15 per group, compared using two- tailed Student’s t test. GOLGA7-sg1, ****p < 0.0001; GOLGA7-sg2, ****p < 0. 0001. **C** Whole cell lysates (WCL) of HeLa cells ± sgGOLGA7 were subjected to fractionation for the detection of the subcellular localization of GFP-NRAS^G12D^ by Western blot analysis. GFP-NRAS^G12D^ were validated using anti-GFP. Subcellular fractions were validated using anti-Na^+^K^+^ATPase (PM) and anti-GAPDH (WCL). **D** Ectopic expression of HA-GOLGA7 in sgGOLGA7-targeted HeLa cell rescues proper NRAS trafficking. **E** Similar experiment of (A) in NRAS^G12D^-transformed Ba/F3 cells

**Fig. 2:**
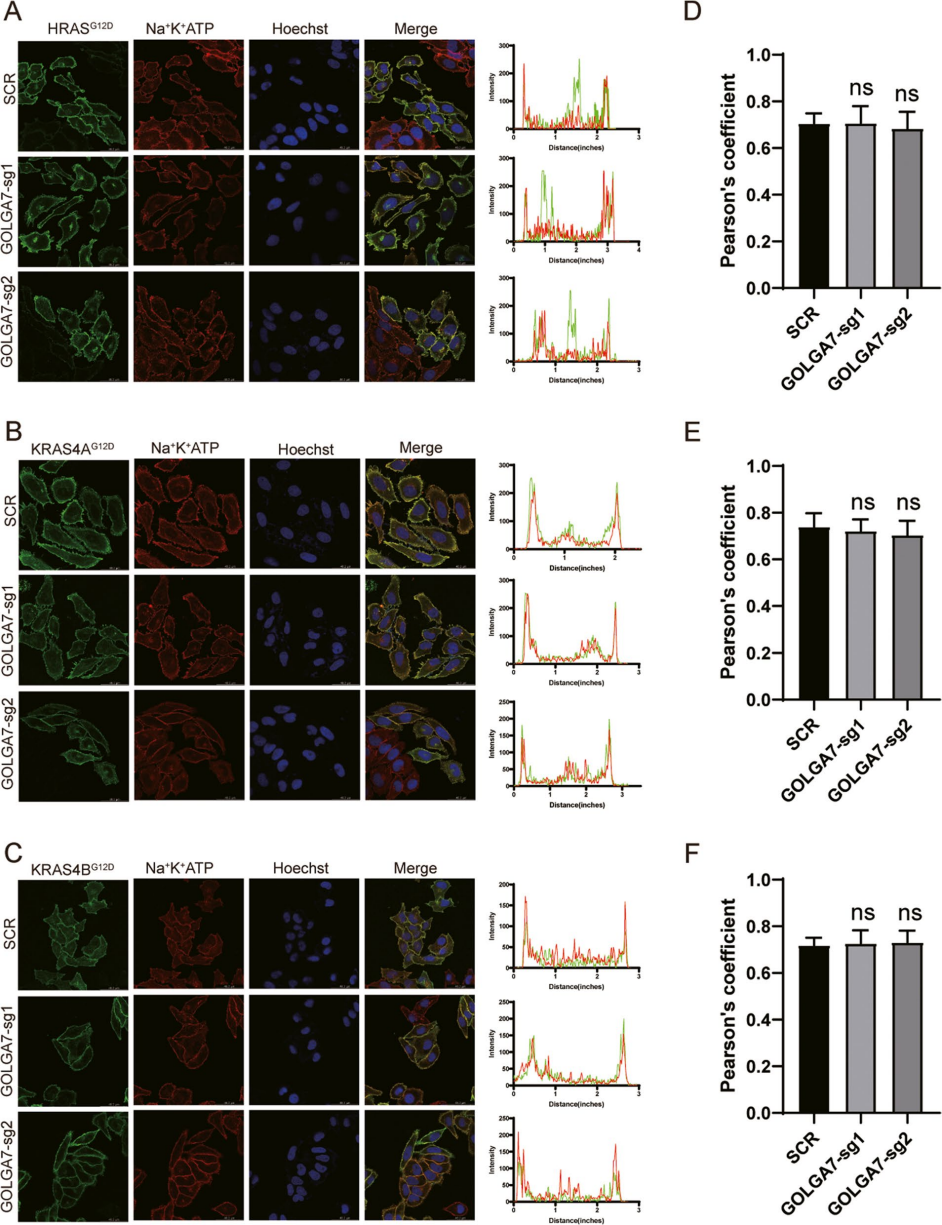
GOLGA7 knockout does not disturb the plasma localization of HRAS, KRAS4A and KRAS4B (**A**-**C**) Immunofluorescence showing the colocalization of the plasma membrane and GFP-HRAS^G12D^ (**A**), GFP-KRAS4A^G12D^ (**B**) and GFP-KRAS4B^G12D^ (**C**) in HeLa cells ± sgGOLGA7. The intensity profiles of GFP-HRAS^G12D^ (**A**), GFP-KRAS4A^G12D^ (**B**) and GFP-KRAS4B^G12D^ (**C**) / the plasma membrane were plotted in the right panel. Scale bars, 42.6 µm. **D**-**F** Statistical quantification of the colocalizations (Pearson’s coefficient) of GFP-HRAS^G12D^/GFP-KRAS4A^G12D^/GFP-KRAS4B^G12D^ and the plasma membrane. Values are the means ± SD from *n* = 15 per group, compared using two- tailed Student’s t test. GFP-HRAS^G12D^ GOLGA7-sg1, ns *p* = 0.9519; GFP-HRAS^G12D^ GOLGA7-sg2, ns *p* = 0.3184; GFP-KRAS4A^G12D^ GOLGA7-sg1, ns *p* = 0.3668; GFP-KRAS4A^G12D^ GOLGA7-sg2, ns *p* = 0.1120; GFP—KRAS4B^G12D^ GOLGA7-sg1, ns *p* = 0.6088; GFP—KRAS4B^G12D^ GOLGA7-sg2, ns *p* = 0.4221

The biological activity of oncogenic NRAS is highly dependent on its localization on the PM [27–29]. To investigate whether GOLGA7 affects the subcellular distribution of oncogenic NRAS, we utilized CRISPR/Cas9 genome editing technology to generate *GOLGA7*-knockout cells. HeLa cells were used for their large size, enabling clear imaging. The efficiency of the knockout was validated by western blot analysis of whole-cell lysates for cells targeted with either a scramble single guide RNA (sgRNA, SCR) or two GOLGA7-targeted sgRNAs (SG1 and SG2) (Fig. S1A). We then transfected GFP-tagged *NRAS*^*G12D*^ constructs into the *GOLGA7*-knockout and control cells. A GFP-tagged palmitoylation-deficient NRAS^G12D^ (NRAS^G12D, C181S^) was used as a non-PM targeting control. Confocal imaging showed that GFP-NRAS^G12D^ was distributed at the PM and in the perinuclear region in control cells, while in the *GOLGA7*-knockout cells, GFP-NRAS^G12D^ showed extensive loss in the PM and was enhanced in the perinuclear region (Fig. 1A). Notably, the Pearson’s coefficient between GFP-NRAS^G12D^ and the PM was significantly reduced in the *GOLGA7*-knockout cells (Fig. 2B). This result was further confirmed by a plasma membrane isolation assay (Fig. 1C).

The altered PM localization was not due to changes in the expression levels of GFP-NRAS^G12D^ proteins, as quantitative immunoblotting of whole cell lysates (WCL) showed that the GFP-NRAS^G12D^ were expressed at equivalent levels (Fig. 1C).

Previous studies have shown that different mutations of RAS have allele-specific biological properties [30]. To determine if GOLGA7 is required for PM localization of other NRAS variants, we expressed GFP-tagged NRAS^WT^, NRAS^G12C^, NRAS^G13D^, or NRAS^Q61R^ in the *GOLGA7*-knockout and control cells, respectively. The mislocalization of NRAS was similar for all variants of NRAS tested (Fig. S1), indicating that the requirement of GOLGA7 for PM localization of NRAS^G12D^ applies to commonly mutated NRAS in cancer.

To exclude the possibility of off-target effects of *GOLGA7* sgRNAs, we restored the PM localization of GFP-NRAS^G12D^ by ectopically expressing HA-tagged GOLGA7 (Fig. 1D).

Similar results were observed in Ba/F3 cells, a murine pro-B cell line, showing diminished PM localization of GFP-NRAS^G12D^ after GOLGA7 depletion (Fig. 1E, Fig. S2B). This finding demonstrates that the requirement of ICMT for GFP-NRAS^G12D^ transport to the PM is independent of the cell type.

Overall, these findings demonstrate that GOLGA7 is necessary for proper trafficking of NRAS to the PM.

### Loss of GOLGA7 does not affect PM localization of HRAS, KRAS4A and KRAS4B

RAS isoforms have both common and distinct motifs for PM targeting. In addition to shared modifications of the CAAX sequence, RAS plasma membrane association requires a “second signal,” which can be either palmitoylation of one (NRAS) or two cysteines (HRAS) or a polybasic stretch of lysine residues (KRAS4B). KRAS4A is also monopalmitoylated but contains an adjacent basic patch that is sufficient to allow plasma membrane localization. These second signal motifs also specify the trafficking routes: HRAS and NRAS transit via the conventional secretory pathway, while KRAS4B transits via a Golgi-independent route [31]. To investigate the effect of GOLGA7 on the plasma membrane association of these other RAS isoforms, we expressed GFP-tagged HRAS^G12D^, KRAS4A^G12D^, and KRAS4B^G12D^ in *GOLGA7*-knockout and control HeLa cells and investigated the PM localization by confocal fluorescence microscopy. Consistent with previous reports [32–35], in control cells, GFP-HRAS^G12D^ was maintained at the PM and perinuclear structures similar to GFP-NRAS^G12D^, while GFP-KRAS4A^G12D^ was predominantly observed at the PM and to a less degree on intracellular vesicles, and GFP-KRAS4B^G12D^ was localized exclusively to the PM with no significant labeling of intracellular structures (Fig. 2A-C). Surprisingly, except for NRAS^G12D^, the PM localization of all other RAS isoforms was not altered in *GOLGA7*-knockout cells (Fig. 1A, Fig. 2A-C). The Pearson’s coefficient analysis and signal quantification result of the other RAS isoforms further validated the results (Fig. 2D-F, Fig. S3-4). These data reveal that GOLGA7 specifically regulates the subcellular trafficking of NRAS.

### NRAS is trapped on the cis-Golgi in GOLGA7-knockout cells

**Fig. 3:**
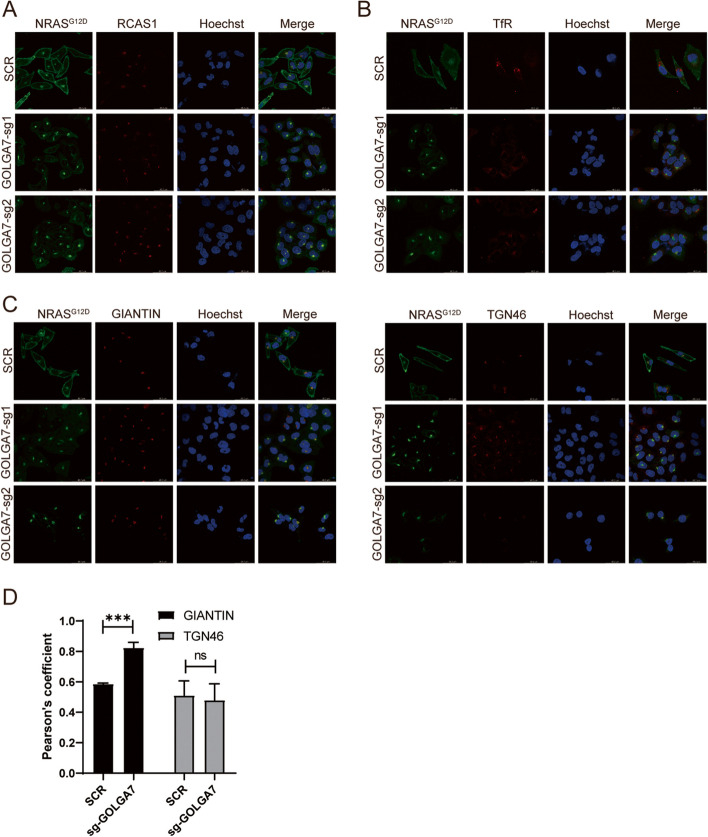
GOLGA7 knockout leads to elevated NRAS co-localization with the *cis*-Golgi (**A**, **B**) Immunofluorescence showing the colocalization of GFP-NRAS^G12D^ and the Golgi (**A**) or recycling endosomes (**B**) in HeLa cells ± sgGOLGA7. Scale bars, 42.6 µm. **C** Immunofluorescence showing the colocalization of GFP-NRAS^G12D^ and GIANTIN (*cis* and *medial* compartment of the Golgi, the upper panel) or TGN46 (*trans* compartment of the Golgi, the bottom panel) in HeLa cells ± sgGOLGA7. Scale bars, 42.6 µm (**D**) Statistical quantification of the colocalizations (Pearson’s coefficient) of GFP-NRAS.^G12D^ and GIANTIN or TGN46. Values are the means ± SD from *n* = 4 per group, compared using two- tailed Student’s t test. GIANTIN, ***p = 0.0001; TGN46, ns *p* = 0.0551

After observing that depletion of GOLGA7 specifically eliminates the PM localization of NRAS, we aimed to identify the precise location where GFP-NRAS^G12D^ displays persistent perinuclear fluorescence in the absence of GOLGA7. We transfected GFP-NRAS^G12D^ into *GOLGA7*-knockout and control HeLa cells and stained subcellular compartment markers of the Golgi (RCAS1) or recycling endosomes (TfR). We discovered strong co-localization of GFP-NRAS^G12D^ with the Golgi marker RCAS1 in both *GOLGA7*-knockout and control HeLa cells (Fig. 3A). However, we found no significant accumulation of GFP-NRAS^G12D^ in recycling endosomes marked by TfR irrespective of GOLGA7 expression (Fig. 3B).

Since the Golgi complex comprises two functionally and structurally distinct networks, namely the *cis* Golgi network (CGN) and the *trans* Golgi network (TGN), responsible for sorting proteins that are received (at the *cis* face) or released (at the *trans* face) by the organelle, we investigated in which of these compartments NRAS was trapped by *GOLGA7* knockout. We transfected GFP-NRAS^G12D^ into *GOLGA7*-knockout and control HeLa cells and assessed its degree of colocalization with markers of the *cis-*Golgi (GIANTIN) or the *trans-*Golgi (TGN46). We observed increased colocalization of GFP-NRAS^G12D^ with the *cis* compartment of the Golgi in GOLGA7 knockout conditions (Fig. 3C, D). In contrast, there were no significant differences in the colocalization of GFP-NRAS^G12D^ with the *trans* compartment of the Golgi compared to control cells (Fig. 3C, D). These findings suggest that GOLGA7 knockout in HeLa cells traps NRAS on the *cis-*Golgi.

### GOLGA7 depletion does not alter the NRAS palmitoylation level

**Fig. 4:**
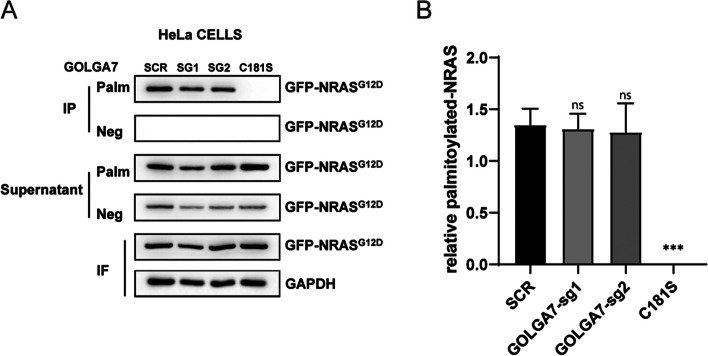
GOLGA7 knockout does not affect NRAS palmitoylation (**A**) Acyl-RAC assays of HeLa cells ± sgGOLGA7 transfected with GFP-NRAS^G12D^ or HeLa cells transfected with GFP-NRAS^G12D, C181S^. Palmitoylation of NRAS^G12D, C181S^ mutant is undetectable, while GFP-NRAS.^G12D^ in HeLa cells ± sgGOLGA7 are all shown to be palmitoylated (Palm in IP). The preserved fraction of immunoprecipitants (Neg in IP) show the specificity of the assay. The cleaved fraction (Palm in Supernatant) and preserved fraction of supernatants (Neg in Supernatant) represent non-palmitoylated proteins and the total protein unbound to the resin, respectively. **B** Statistical quantification of relative palmitoylated protein in (A). Values are the means ± SD from three independent experiments, compared using two-sided Student’s t test. GOLGA7-sg1, ns *p* = 0.7751; GOLGA7-sg2, ns *p* = 0.7229; C181S, ***p = 0.0001

Previous studies have highlighted the critical role of palmitoylation in the association of NRAS with the plasma membrane, indicating its essentiality [6, 19–21]. GOLGA7 has been found to form a complex with the protein palmitoyltransferase (PAT) ZDHHC9 in vitro to facilitate the palmitoylation of HRAS and NRAS [21]. In addition, GOLGA7 has also been shown to form complexes with DHHC14 or DHHC18, suggesting its potential as a common accessory protein for DHHCs [24]. Recent research has revealed, through the cryo-EM structure of the ZDHHC9-GOLGA7 complex, that GOLGA7 does not directly participate in the catalytic process but instead stabilizes the architecture of ZDHHC9 [24]. As a result, one possibility we considered was that GOLGA7 knockout could lead to reduced PM localization of NRAS via disturbing the regulation of its palmitoylation. To test this possibility, we measured the palmitoylation level of NRAS in *GOLGA7*-knockout and control HeLa cells stably expressing GFP-NRAS^G12D^ using an S-palmitoylation acyl-RAC assay. GFP-NRAS^G12D, C181S^ was used as a non-palmitoylated NRAS control and a GFP antibody was used for detection of these GFP-NRAS proteins. As expected, in control cells, palmitoylated GFP-NRAS was detected in the pull-down fraction (Palm in IP) of cells stably expressing GFP-NRAS^G12D^ but not of cells expressing GFP-NRAS^G12D, C181S^ (Fig. 4A, B). Interestingly, unlike the complete abrogation of GFP-NRAS^G12D, C181S^ palmitoylation, no significant reduction of palmitoylated GFP-NRAS^G12D^ was detected in *GOLGA7*-knockout cells (Fig. 4A-B). Similar results were obtained in Ba/F3 cells (Fig. S5). These findings suggest that mislocalization of GFP-NRAS was not a consequence of disrupted palmitoylation. They also highlight that GOLGA7 is more than an accessory protein of palmitoyltransferase and participates in the NRAS trafficking process.

### GOLGA7 regulates anterograde transport of NRAS exit from the Golgi

**Fig. 5:**
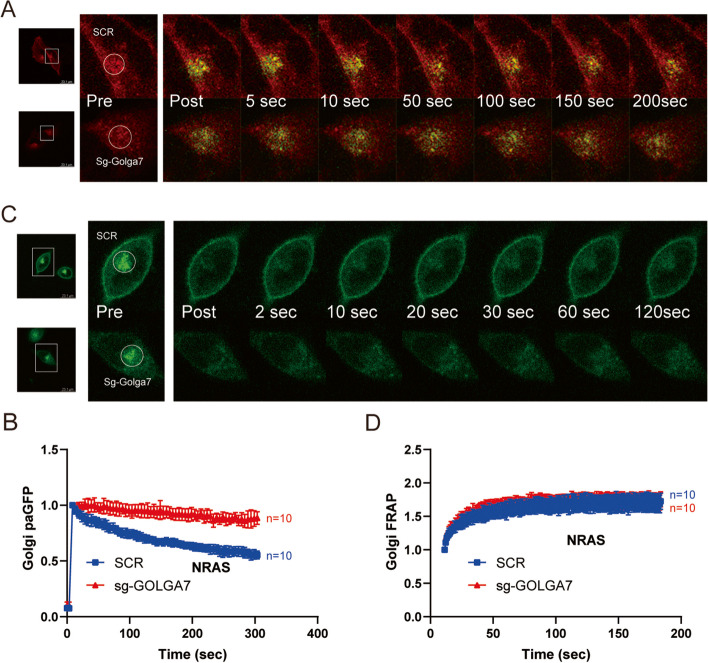
GOLGA7 regulates anterograde trafficking of NRAS away from the Golgi (**A**) Anterograde trafficking of NRAS away from the Golgi was assessed via photoactivation of paGFP-NRAS on the Golgi (marked by mCherry-NRAS) and monitoring fluorescence loss over time; a representative cell is shown. Bars indicate 46.2 µm. **B** Curves trend the loss of initial fluorescence post-photoactivation in the region of the Golgi in cells ± sgGOLGA7 as mean ± SD for cells examined. Values are the means ± SD from *n* = 10 per group. **C** Retrograde trafficking of NRAS to the Golgi was examined using GFP-NRAS^G12D^ expressed in HeLa cells with FRAP kinetics determined in the region of the Golgi; a representative cell is shown. Bars indicate 46.2 µm. **D** Recovery curves trend the mean ± SD of the calculated fraction of initial fluorescence over time in the cells examined. Values are the means ± SD from *n* = 10 per group

After finding that GOLGA7 does not regulate the PM localization of NRAS through palmitoylation, we investigated its potential role in protein transport. Specifically, we examined whether GOLGA7 is involved in NRAS cycling between the Golgi and the PM. Using a two-color photoactivatable mCherry-PAGFP(G(PA)C) probe, we analyzed the anterograde transport of NRAS away from the Golgi with 10 cells per group. Our results showed that NRAS was blocked on the Golgi in *GOLGA7*-knockout HeLa cells (Fig. 5B), indicating decreased anterograde flux of NRAS due to GOLGA7 knockout (Fig. 5A, B). Additionally, we used a fluorescence recovery after photobleaching (FRAP) assay (Fig. 5C) to measure retrograde transport of NRAS from the PM and cytosol to the Golgi. We found that there was no significant difference in NRAS recovery in the presence or absence of GOLGA7 with 10 cells per group examined (Fig. 5D). It is worth noting that the anterograde movement of NRAS from the Golgi to the PM is slower than the retrograde movement from the PM to the Golgi (Fig. 5B, D), consistent with the non-vesicular pathway for NRAS retrograde transport [11] and the vesicular pathway for anterograde transport [9, 10, 12].

To assess if GOLGA7 knockout caused structural disruption, we examined the structure of the Golgi and the cytoskeleton in *GOLGA7*-knockout and control HeLa cells. Immunofluorescence analysis of the Golgi marker GM130 showed no significant physical disruption of the Golgi apparatus (Fig. S6A), and phalloidin-labeled actin or tubulin did not appear to be affected either (Fig. S6B, C). Therefore, it seems unlikely that altered structure of the Golgi or cytoskeleton explains NRAS mislocalization induced by GOLGA7 knockout.

Vesicular stomatitis virus G protein (VSV-G) is commonly used to visualize cellular protein transport, and studies have shown that overexpression of wild type GOLGA7 retains VSV-G in the Golgi [25]. Thus, we used a temperature-sensitive and GFP-tagged VSV-G protein (ts045-VSVG-EGFP) to examine the effect of GOLGA7 knockout on protein transport in the secretory pathway in HeLa cells. The ts045-VSVG-EGFP protein was retained in the endoplasmic reticulum at a nonpermissive temperature (40 °C) and transported to the cell surface via the Golgi complex at a permissive temperature (32 °C) in both the *GOLGA7*-knockout and control HeLa cells identically, as revealed by the GFP image (Fig. S7). This result indicates that the transport of NRAS is different from VSV-G, and GOLGA7 knockout does not affect the classical secretion pathway.

These results suggest that GOLGA7 knockout inhibits the anterograde transport of NRAS from the Golgi.

### GOLGA7 is required for the malignant transformation of oncogenic NRAS

**Fig. 6:**
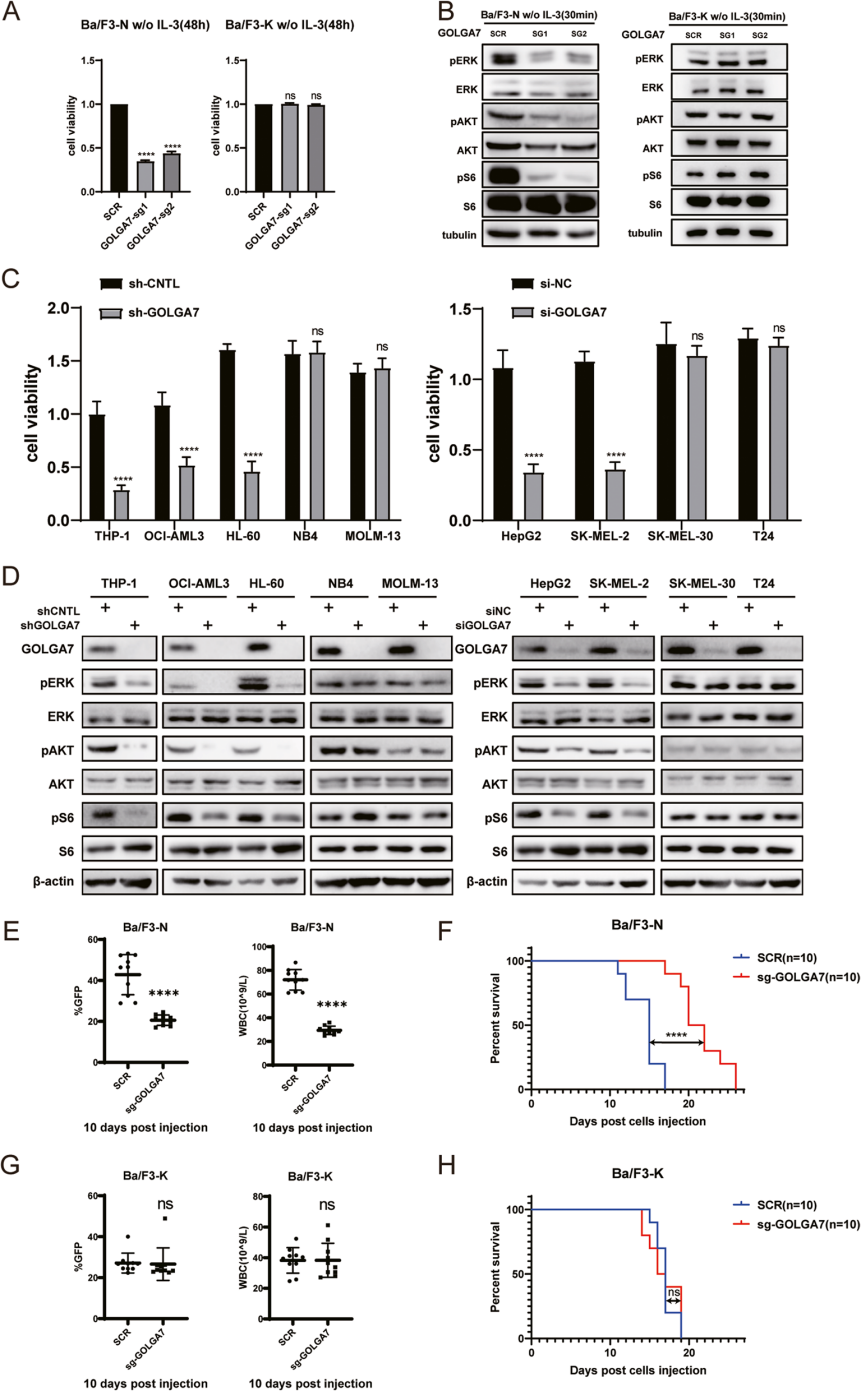
GOLGA7 is essential for the viability of NRAS-dependent cells and the transformation of oncogenic NRAS in mice. **A** Ba/F3-N cells (left panel) or Ba/F3-K cells (right panel) ± sgGOLGA7 were cultured without(w/o) IL-3. Cell viability of each cell line was measured by CellTiter-Glo Luminescent Cell Viability Assay 48 h after plating. Values are the means ± SD from three independent experiments, compared using two-sided Student’s t test. Ba/F3-N GOLGA7-sg1, ****p < 0.0001; Ba/F3-N GOLGA7-sg2, ****p < 0.0001; Ba/F3-K GOLGA7-sg1, ns *p* = 0.5134; Ba/F3-K GOLGA7-sg2, ns *p* = 0.2820. **B** Ba/F3-N cells (left panel) and Ba/F3-K cells (right panel) were cultured without(w/o) IL-3 for 30 min and analyzed subsequently by Western blotting using total or phospho-ERK, -AKT and -S6 antibodies. β-Actin was used as a loading control. **C** Cell viability of human cancer cell lines ± shGOLGA7/siGOLGA7 was detected by the CellTiter Glo assay. Values are the means ± SD from three independent experiments, compared using two-sided Student’s t test. Cell lines with mutant NRAS: THP-1(NRAS^G12D^), ****p < 0.0001; OCI-AML3(NRAS^Q61L^), ****p < 0.0001; HL-60(NRAS^Q61L^), ****p < 0.0001; HepG2(NRAS^Q61L^), ****p < 0.0001; SK-MEL-2(NRAS^Q61R^), ****p < 0.0001. Cell lines with wild-type NRAS: MOLM-13(NRAS^WT^), ns *p* = 0.4193; SK-MEL-30(NRAS^WT^), ns *p* = 0.2299. Cell lines with mutant HRAS: T24(HRAS^G12V^), ns *p* = 0.7229. Cell lines with mutant KRAS4B: NB4(KRAS4B.^A18D^), ns *p* = 0.1698. **D** Cells in (**C**) were analyzed by Western blotting using total or phospho-ERK, -AKT and -S6 antibodies. β-actin was used as a loading control. **E** The percentage of GFP-positive Ba/F3-N cells and the number of white blood cells in peripheral blood (PB) samples from mice with Ba/F3-N ± sgGOLGA7 cells injection after 10 days. ****p < 0.0001. **E** Kaplan–Meier survival curves of lethally irradiated mice with Ba/F3-N ± sgGOLGA7 cells injection. **** *P* < 0.0001. **G** The percentage of GFP-positive Ba/F3-K cells and the number of white blood cells in peripheral blood (PB) samples from mice with Ba/F3-K cells ± sgGOLGA7 injection after 10 days. ns *p* = 0.9853. **H** Kaplan–Meier survival curves of lethally irradiated mice with Ba/F3-K ± sgGOLGA7 cells injection, ns *p* = 0.9303

RAS is known to propagate growth factor signaling, particularly the mitogenic MAPK signaling pathway (Raf/MEK/ERK) and the PI3K/AKT/mTOR survival pathways. To investigate the impact of GOLGA7 on RAS signaling, we used GFP-tagged NRAS^G12D^ or KRAS4B^G12D^ transformed Ba/F3 cells (Ba/F3-N or Ba/F3-K cells), a murine cell line reliant on the oncogenic NRAS or KRAS4B under IL-3 deprivation [36–38]. Knocking out* Golga7* in IL-3 deprived Ba/F3-N cells significantly impaired cell proliferation (Fig. 6A), concurrently decreasing phospho-ERK, phospho-AKT, and phospho-S6 levels (Fig. 6B). These observations extended to human cancer cell lines, where depletion of GOLGA7 substantially inhibited proliferation in cell lines with *NRAS* mutations (THP-1, OCI-AML3, HL-60, HepG2, and SK-MEL-2), but not in NRAS-wild type cells (SK-MEL-30, and MOLM-13), or cells with HRAS or KRAS4B mutations (T24 and NB4, respectively) (Fig. 6C). Likewise, the levels of phospho-ERK, phospho-AKT, and phospho-S6 were reduced upon GOLGA7 depletion in cells with oncogenic NRAS but not cells with wild-type NRAS or other oncogenic RAS mutants (Fig. 6D).

We further transplanted the aforementioned Ba/F3 cell lines into the syngeneic BALB/c mouse strain to test the efficacy in vivo. Briefly, *Golga7*-knockout Ba/F3 cells were generated using CRISPR/Cas9 and engineered to stablely expess GFP-tagged NRAS^G12D^ or KRAS4B^G12D^ at the presence of IL-3. Subsequently, these *Golga7*-knockout Ba/F3-N or Ba/F3-K cells were transplanted into lethally irradiated recipient mice. IL-3 was withdrawn from the cells immediately before transplantation to switch their dependency on the oncogenic RAS. Mice receiving Ba/F3-N or Ba/F3-K cells expressing the endogenous *Golga7* were used as controls. Compared to the control group, *Golga7* knockout Ba/F3-N group showed lower percentage of GFP-positive cells and white blood cell (WBC) counts in the peripheral blood 10 days after transplantation, indicating less leukemia burden (Fig. 6E). Furthermore, mice receiving *Golga7*-knockout Ba/F3-N cells also survived a much longer time (median survival 20 vs. 15 days, Fig. 6F). In contrast, no significant differences of GFP-positive cell population and WBC counts were found in the Ba/F3-K model (Fig. 6G) and *Golga7*-knockout failed to prolong the survival time in mice receiving Ba/F3-K cells (Fig. 6H). Taken together, these results indicate that *Golga7* plays an important role in efficient transformation of NRAS^G12D^, and *Golga7* deficiency could significantly suppress the ability of oncogenic NRAS but not KRAS4B at both in vitro and in *vivo* level.

## Discussion

In this study, we discovered that GOLGA7 is essential for the PM localization of NRAS, but not for HRAS, KRAS4A, or KRAS4B. Remarkably, the altered localization of NRAS in GOLGA7-depleted cells occurred without any changes in palmitoylation status. Our research also demonstrated that the knockout of GOLGA7 traps NRAS in the *cis*-Golgi and obstructs the anterograde transport of NRAS exit from the Golgi. Additionally, GOLGA7 knockout inhibited and the ability of oncogenic NRAS both in vitro and in vivo. Our findings offer new insights into GOLGA7’s function in NRAS trafficking and suggest the potential for GOLGA7 as a specific therapeutic target for NRAS.

The findings of our study suggest that the role of GOLGA7 in NRAS palmitoylation is redundant since it is essential for PM localization but not palmitoylation. GOLGA7 is known to be the mammalian homologue of Erf4, the accessory protein for the ZDHHC enzyme Erf2 in yeast [21, 39]. Studies have demonstrated that the palmitoylation of NRAS and HRAS at cysteine residues in humans is catalyzed by the acyltransferase ZDHHC9 in complex with its accessory protein GOLGA7 [21, 22, 24]. However, the cryo-EM structure of the ZDHHC9-GOLGA7 complex suggests that GOLGA7 stabilizes the architecture of ZDHHC9 rather than being directly involved in the catalytic process through four interfaces [24]. In human, 23 ZDHHC acyltransferases (ZDHHCs) have been identified and the redundancy of ZDHHCs has been indicated by several studies [23, 40, 41]. Previous studies have implicated the redundancy of ZDHHC acyltransferases, including ZDHHC9, in NRAS palmitoylation [22]. Studies have shown that ZDHHC6 [42], ZDHHC14, and ZDHHC18 [24, 43] can also catalyze NRAS palmitoylation. Similarly, other accessory proteins, such as GOLGA7B, SelK, and HTT, have been reported to interact with ZDHHCs [44]. However, the specific role of these accessory proteins in NRAS palmitoylation needs to be further investigated, as their ability to compensate for the function of GOLGA7 in NRAS palmitoylation is not yet clear. Furthermore, GOLGA7B interacts with ZDHHC5 and ZDHHC8, similar to GOLGA7 [43, 45]. However, siRNA-mediated depletion of GOLGA7B did not rescue the effect on the plasma membrane localization of ZDHHC5, even with the expression of GOLGA7 [45]. This suggests that GOLGA7B may have a specific role in its interaction with ZDHHC5. In addition, while NRAS palmitoylation can be catalyzed by ZDHHC6, ZDHHC9, ZDHHC14, and ZDHHC18, GOLGA7B is found to potentiate only ZDHHC5 and ZDHHC8 [43]. Therefore, it is unclear whether GOLGA7B contributes to the unchanged palmitoylation level of NRAS in the absence of GOLGA7. To explain this finding, other accessory proteins of the pamitoyltransferases need to be identified and studied, including SelK, a potential candidate for ZDHHC-mediated NRAS palmitoylation [42]. Further investigation is required to understand the involvement of other accessory proteins in NRAS palmitoylation in conditions of GOLGA7 knockout.

One of the most noteworthy findings of this study is that GOLGA7 showed selectivity for NRAS but not for other RAS isoforms, particularly HRAS, which undergoes similar post-translational modifications and shares a similar trafficking route to the PM through vesicle transport from the Golgi [9, 10, 12]. Although KRAS4A is also monopalmitoylated, it contains a weaker polybasic motif that allows for PM targeting [6]. Several differences in the trafficking of HRAS and NRAS have been examined. For example, the differential palmitoylation states of NRAS and HRAS determine their distinct Golgi localization patterns [46]. While the doubly palmitoylated HRAS is distributed throughout the Golgi stacks, singly palmitoylated NRAS is polarized with a relative paucity of expression on the *trans*-Golgi [46]. As a result, it is possible that NRAS takes an alternate path to the PM on vesicles derived from the *cis*- or *medial*-Golgi and requires GOLGA7 to bud from these Golgi stacks. This would also explain the elevated expression of NRAS on the *cis*-Golgi in conditions of GOLGA7 knockout. The observation that some NRAS-containing, but not HRAS-containing, vesicles were occasionally seen adjacent to Golgi cisternae further supports the model [47]. Chaperones for RAS trafficking also differ in their selectivity for these isoforms, such as VPS35 for NRAS[35] and Nogo-B receptor (NgBR) for HRAS [48]. Therefore, it is also possible that GOLGA7 serves as a selective chaperone that shuttles NRAS. The fact that GOLGA7 localizes to both the Golgi and the PM further supports this hypothesis.

There have been several studies linking GOLGA7 to protein transport. When the wild-type GOLGA7 is overexpressed, it can cause an inhibitory effect on the transport of VSV-G proteins from the Golgi. This effect is considered dominant-negative, as similar results have been observed in other proteins, including GCP60 [25]. GOLGA7 has also been found to stabilize ZDHHC5, an enzyme that regulates retrograde transport from the PM [26]. In the present study, we discovered that GOLGA7 regulates the anterograde trafficking of NRAS, demonstrating its non-canonical role in the transport process, which is independent of palmitoylation. However, the underlying mechanisms remain to be identified.

**Fig. 7:**
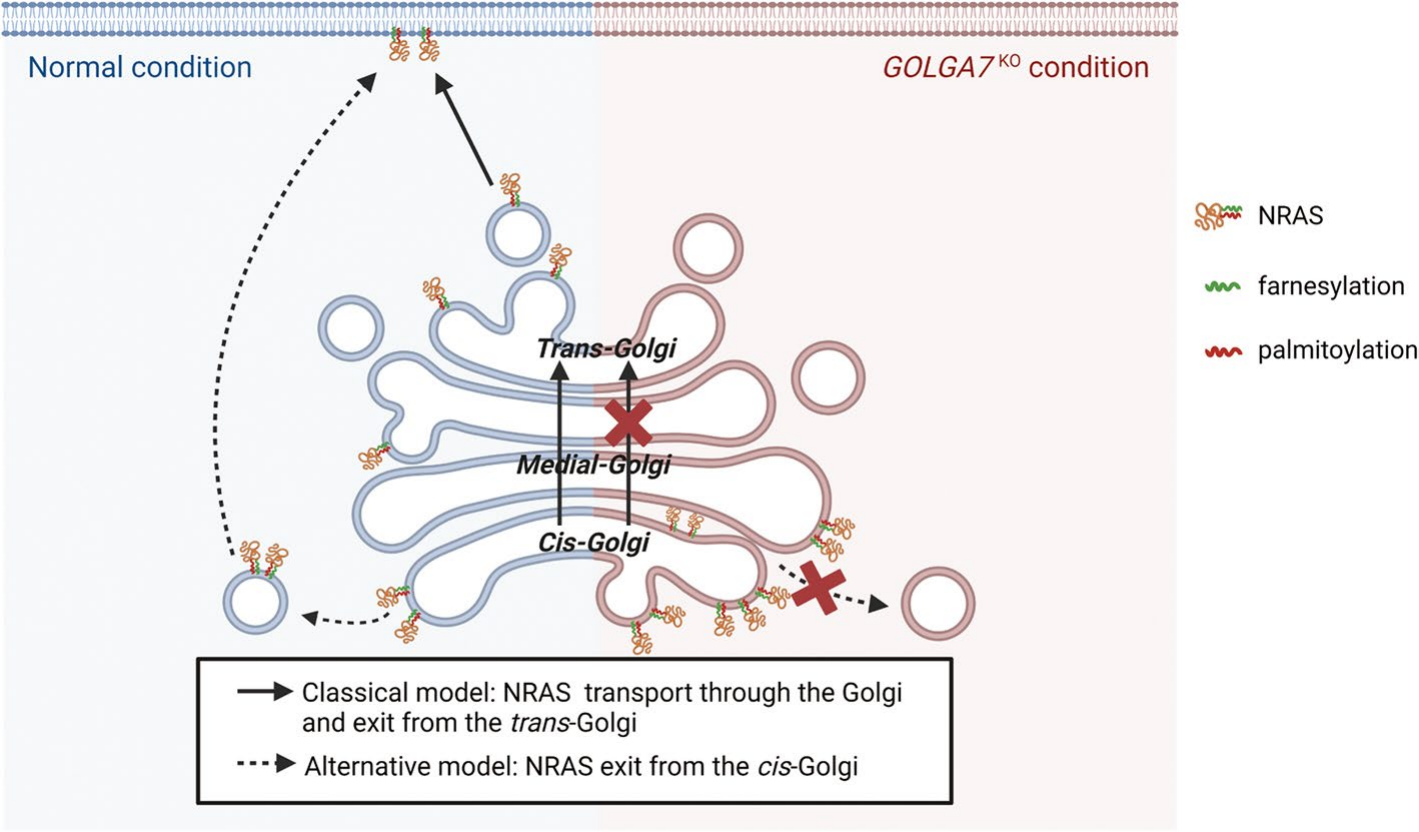
Potential models for GOLGA7 regulation on NRAS transport based on its different exit sites from the Golgi. (Full arrows) classical model: in the normal condition, NRAS undergoes conventional trafficking from the *cis*-Golgi to the *trans*-Golgi and then reaches the plasma membrane. In this case, GOLGA7 may regulate the anterograde trafficking of NRAS from the *cis*-Golgi to the *trans*-Golgi. In the condition of GOLGA7 depletion, the intra-Golgi transport of NRAS is disturbed and results in its accumulation at the *cis*-Golgi. (Dashed arrows) alternative model: in the normal condition, NRAS vesicles can bud directly from the *cis*-Golgi. In this case, GOLGA7 may play a role in the budding process of NRAS. In the condition of GOLGA7 depletion, the budding process of NRAS is disturbed and results in its accumulation at the *cis*-Golgi. Image created with BioRender.com

Based on our findings that GOLGA7 depletion results in NRAS accumulation in the *cis*-Golgi and inhibits its anterograde trafficking from the Golgi, two potential models have emerged (Fig. 7). If NRAS undergoes conventional trafficking from the *cis*-Golgi to the *trans*-Golgi before reaching the PM, GOLGA7 may regulate its anterograde trafficking within the Golgi. Alternatively, if NRAS is transported directly from the *cis*-Golgi to the PM, then GOLGA7 may regulates its budding from the Golgi. Nevertheless, our data supports that GOLGA7 is most likely to control the budding of NRAS from the *cis*-Golgi. A recent genome-wide CRISPR screening study identified GOLGA7 as a NRAS co-dependent gene in leukemia cell lines with or without NRAS mutation [49]. This finding supports our discovery that GOLGA7 depletion selectively affects NRAS-mutant cells, suggesting that targeting GOLGA7 may have a favorable safety profile since cells without NRAS mutation are unaffected.

In conclusion, we demonstrated that GOLGA7 is essential for the PM localization of NRAS in a non-palmitoylation dependent way. Of note, our data revealed that the PM translocation under GOLGA7 regulation is uniquely required by NRAS but not other RAS isoforms. Altogether, our findings elucidate a specific intracellular trafficking route for NRAS under GOLGA7 regulation and suggest the potential for GOLGA7 as a specific therapeutic target for NRAS.

### Supplementary Information

**Additional file 1.**

**Additional file 2.**

**Additional file 3.**

## Data Availability

No datasets were generated or analysed during the current study.
