# Supplementary material for: GOLGA7 is essential for NRAS trafficking from the Golgi to the plasma membrane but not for its palmitoylation
Source: Cell Commun Signal. 2024 Feb 5;22:98. doi: 10.1186/s12964-024-01498-w (PMC10845536; doi:10.1186/s12964-024-01498-w)

Full and uncropped western blot for Figure 1C Lanes 1-4 are on the figure

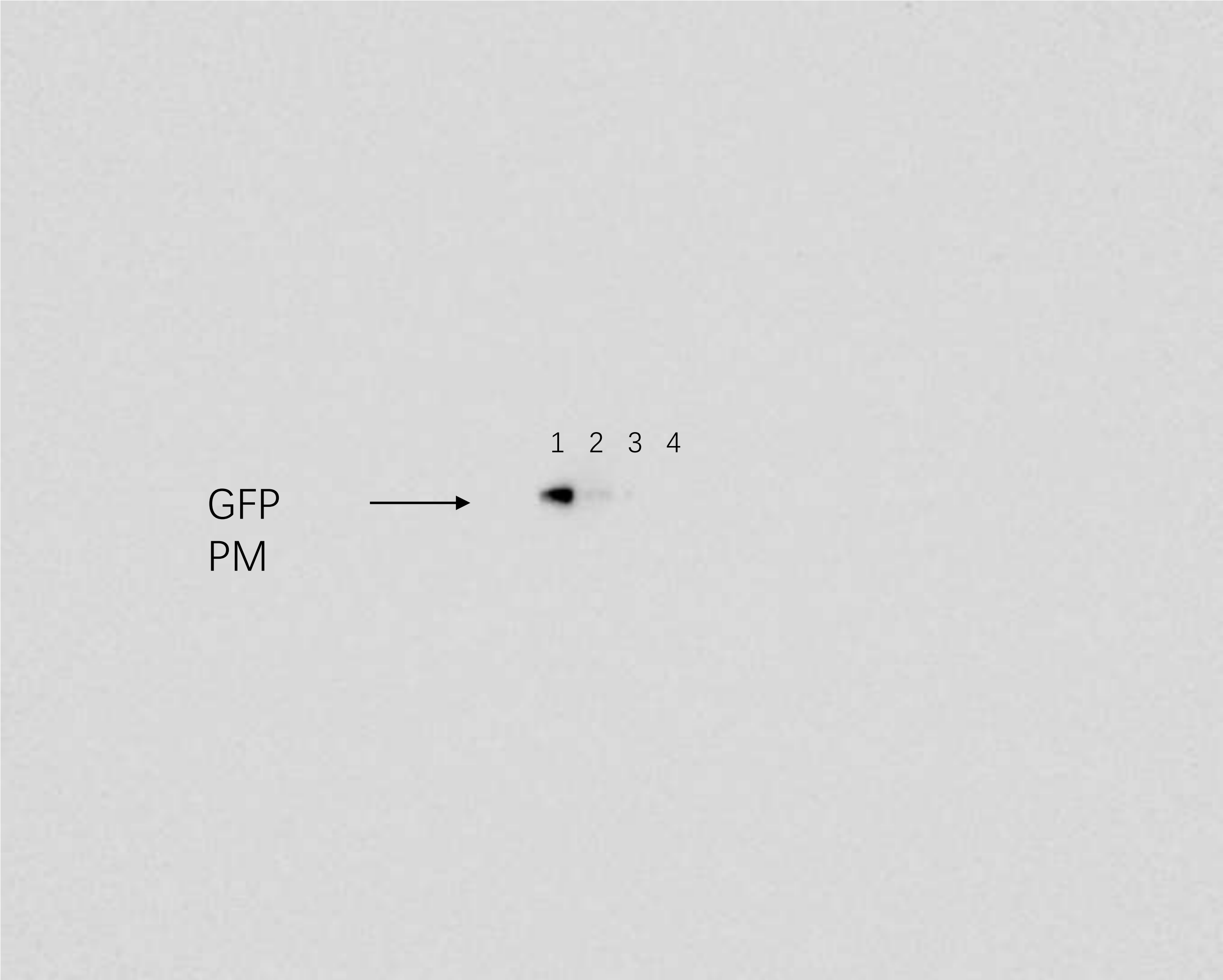

Full and uncropped western blot for Figure 1C Lanes 1-4 are on the figure

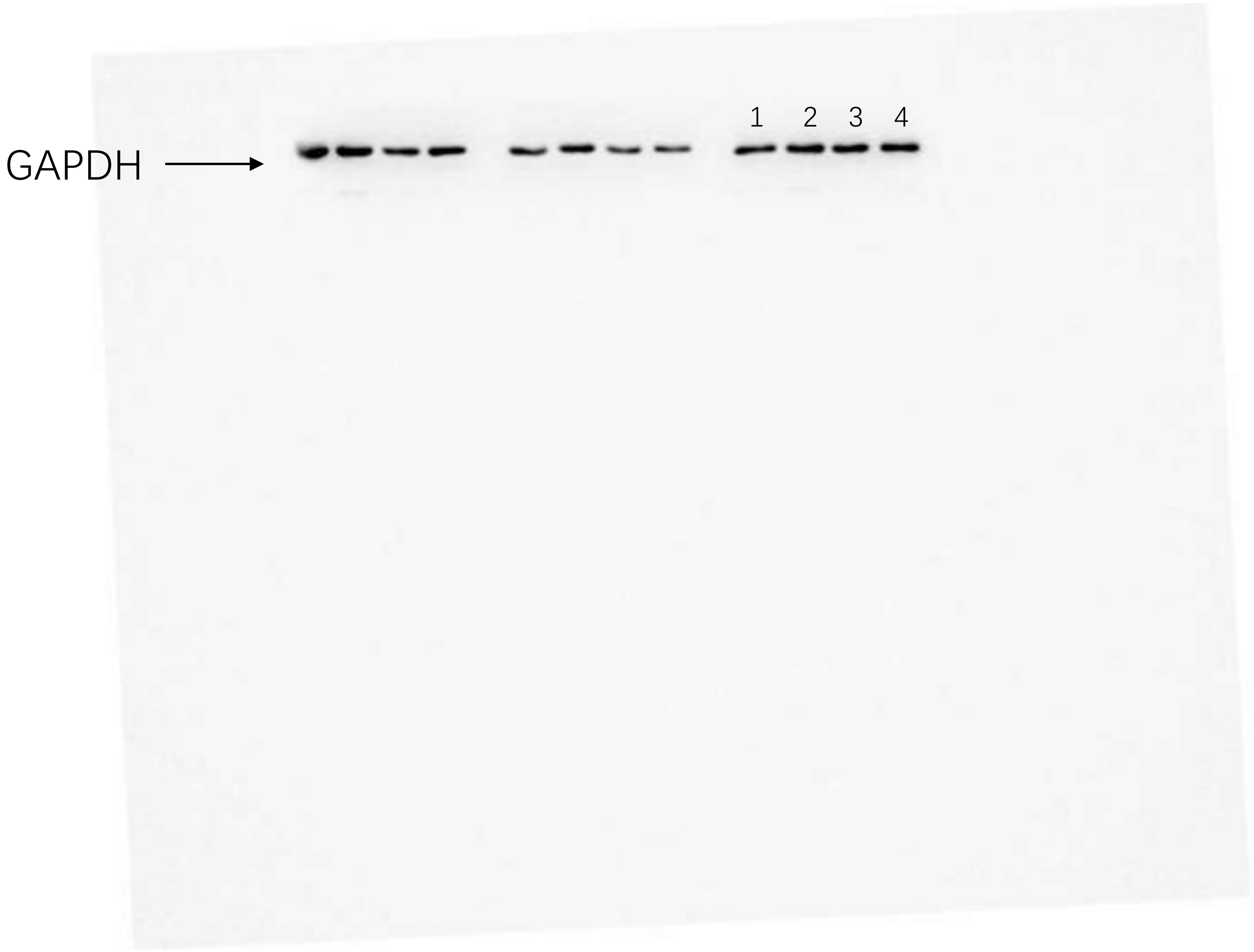

Full and uncropped western blot for Figure 1C Lanes 1-4 are on the figure

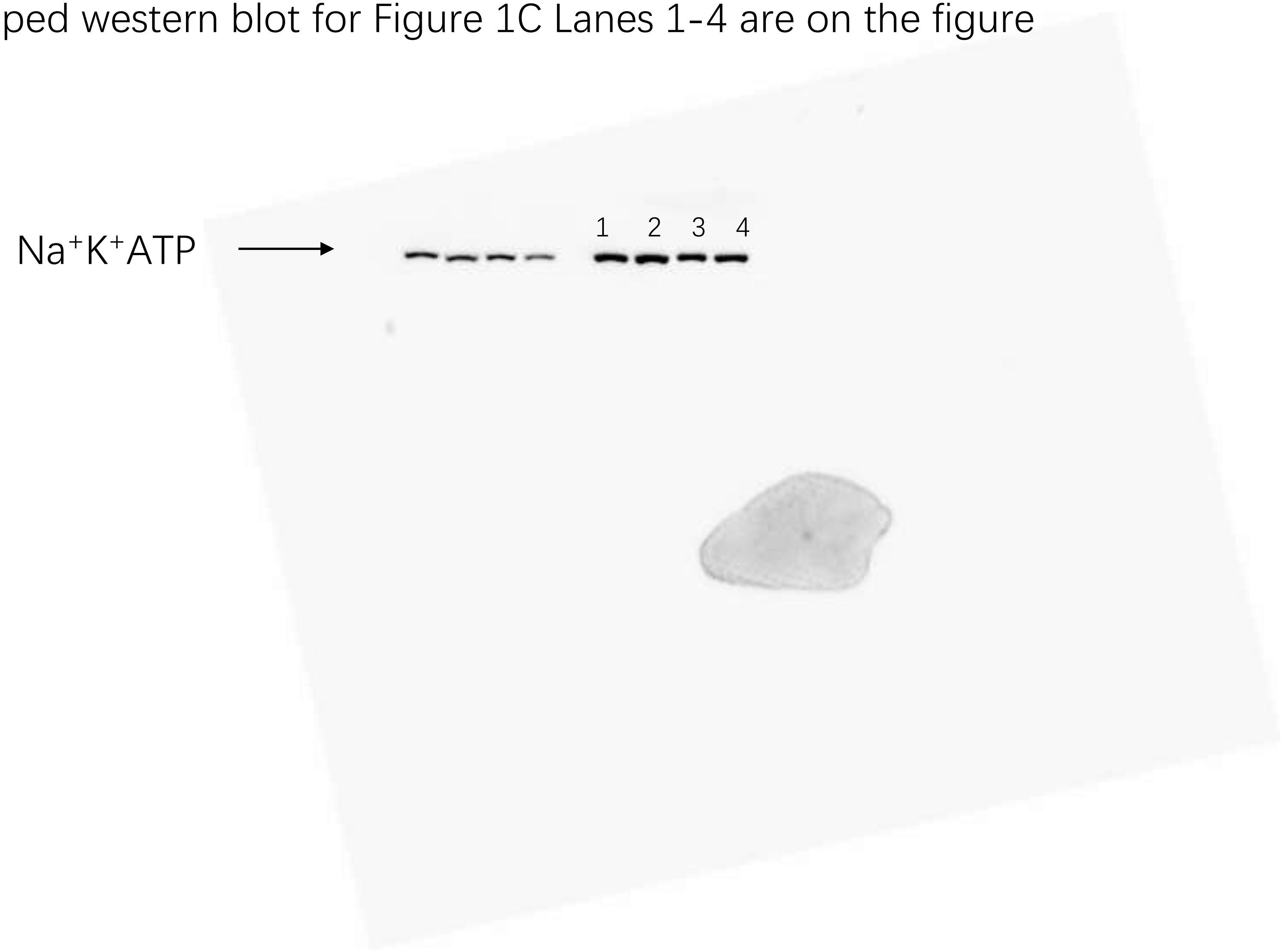

Full and uncropped western blot for Figure 1C Lanes 1-4 are on the figure

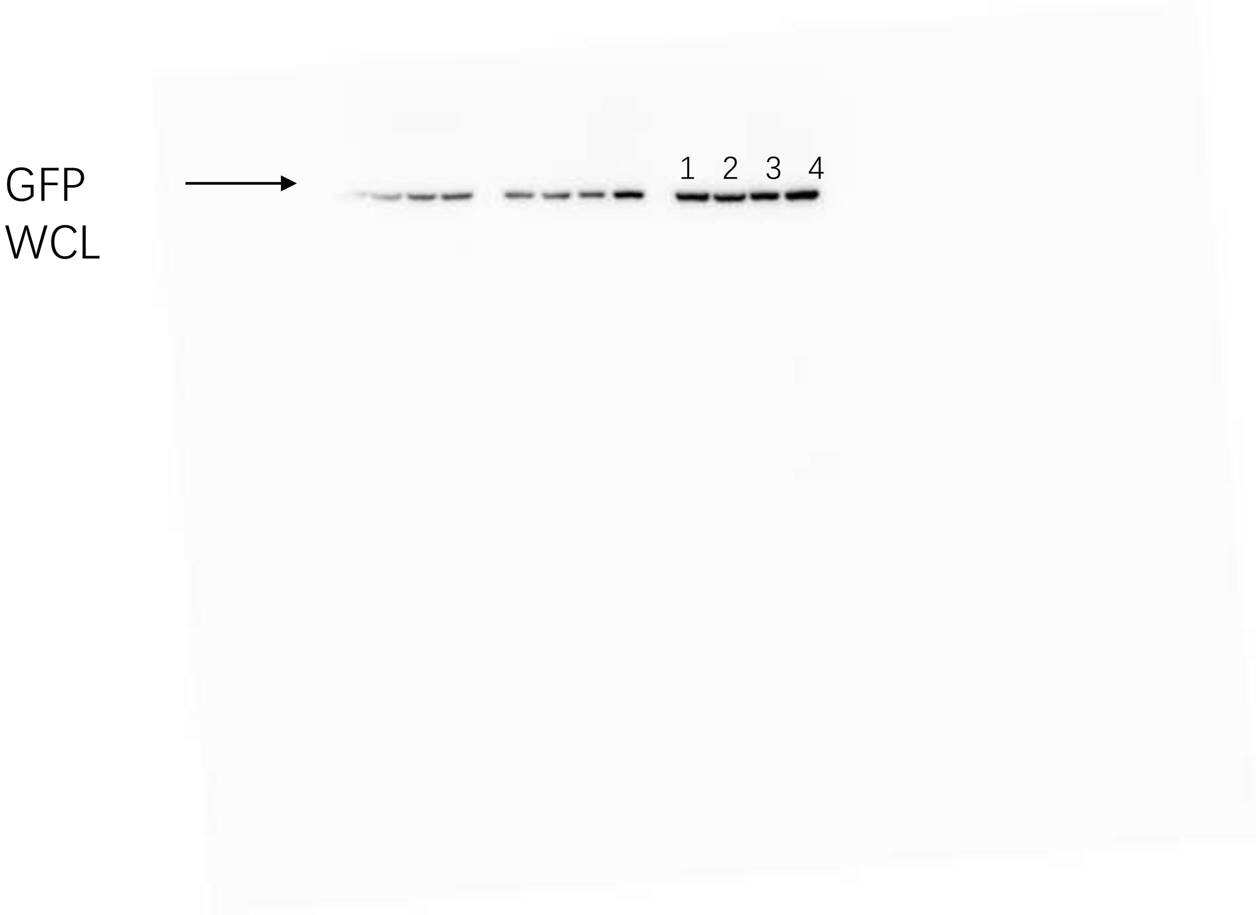

Full and uncropped western blot for Figure 5A Lanes 1-4 are on the figure

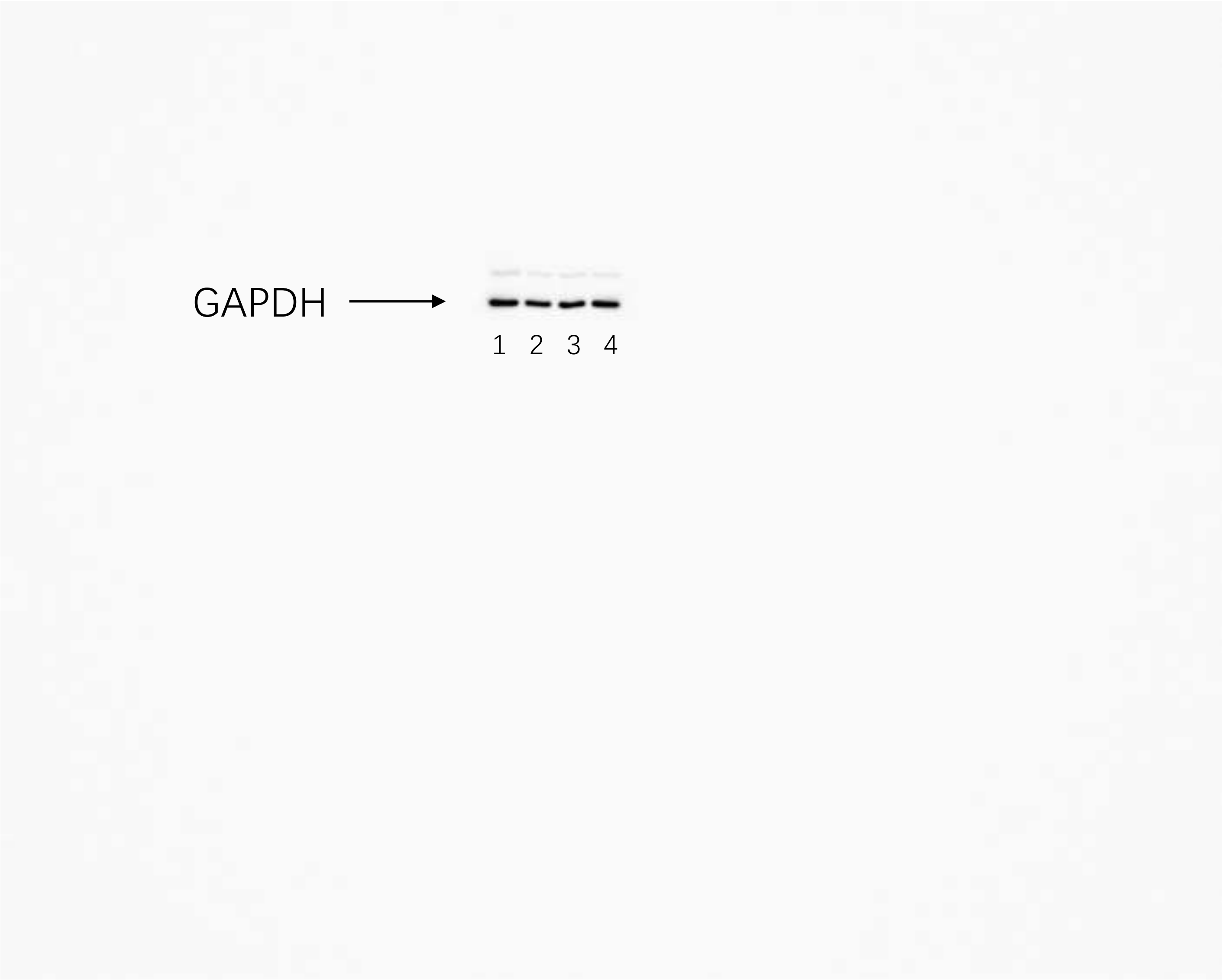

Full and uncropped western blot for Figure 5A Lanes 1-4 are on the figure

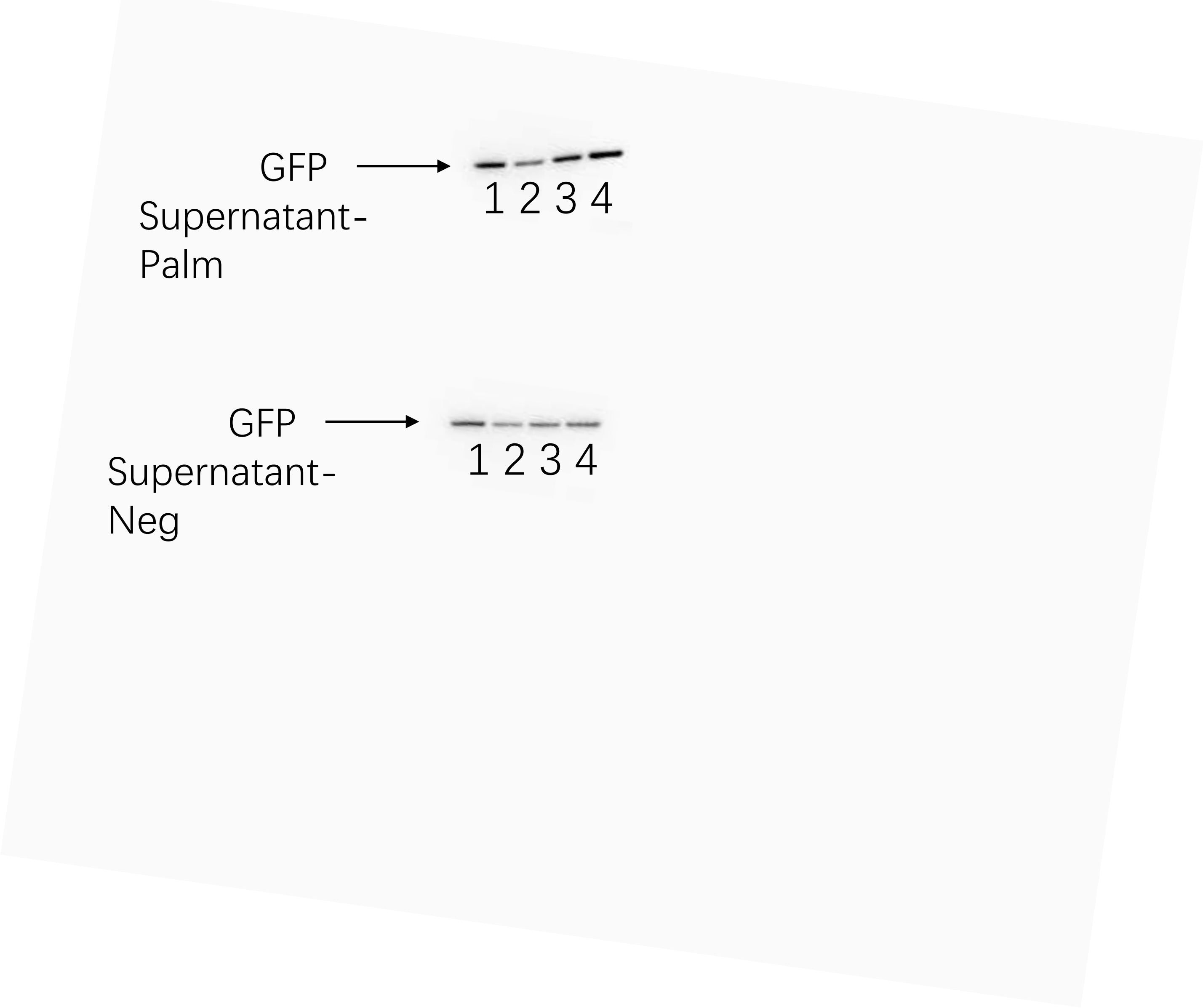

Full and uncropped western blot for Figure 5A Lanes 1-4 are on the figure

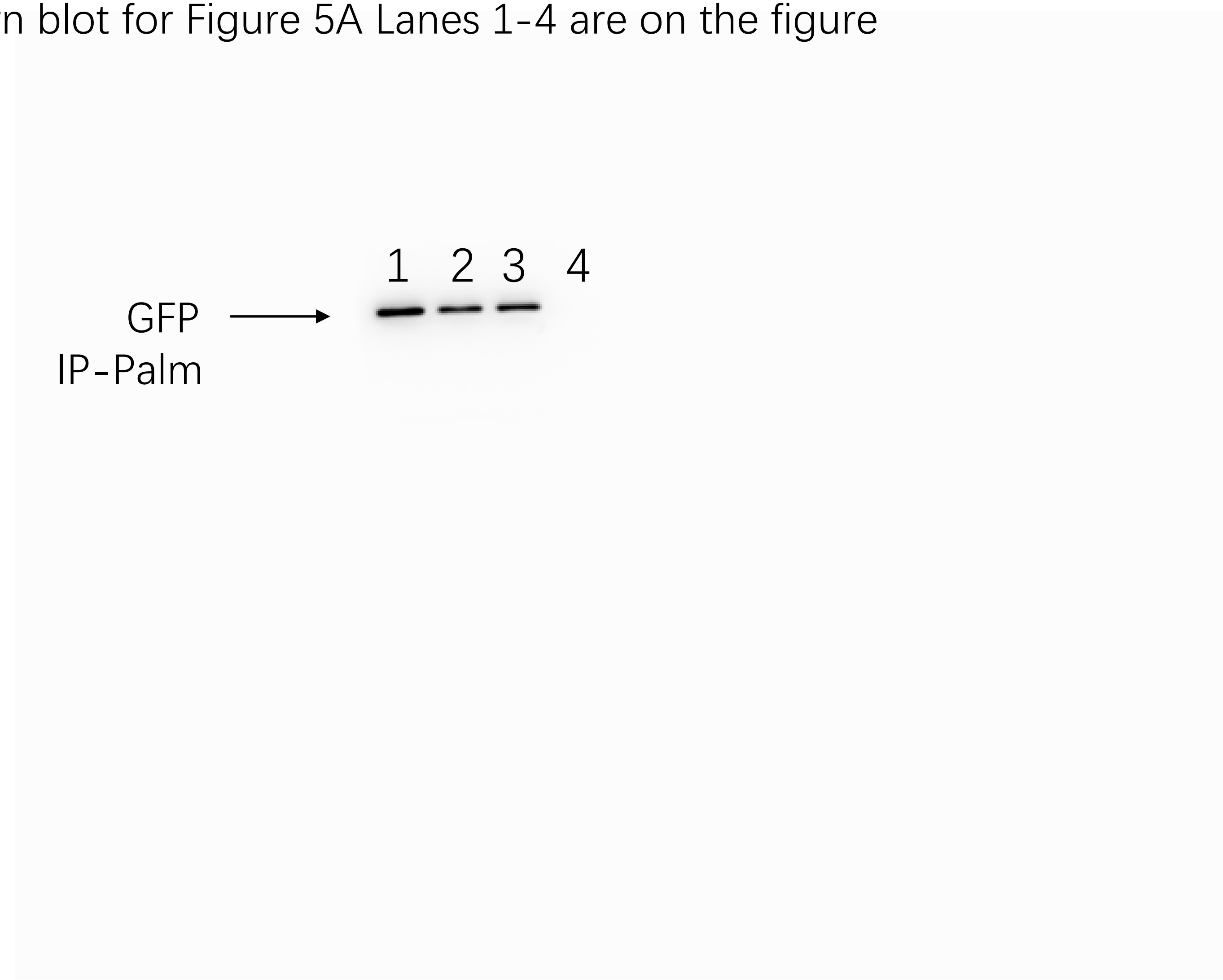

Full and uncropped western blot for Figure 5A Lanes 1-4 are on the figure

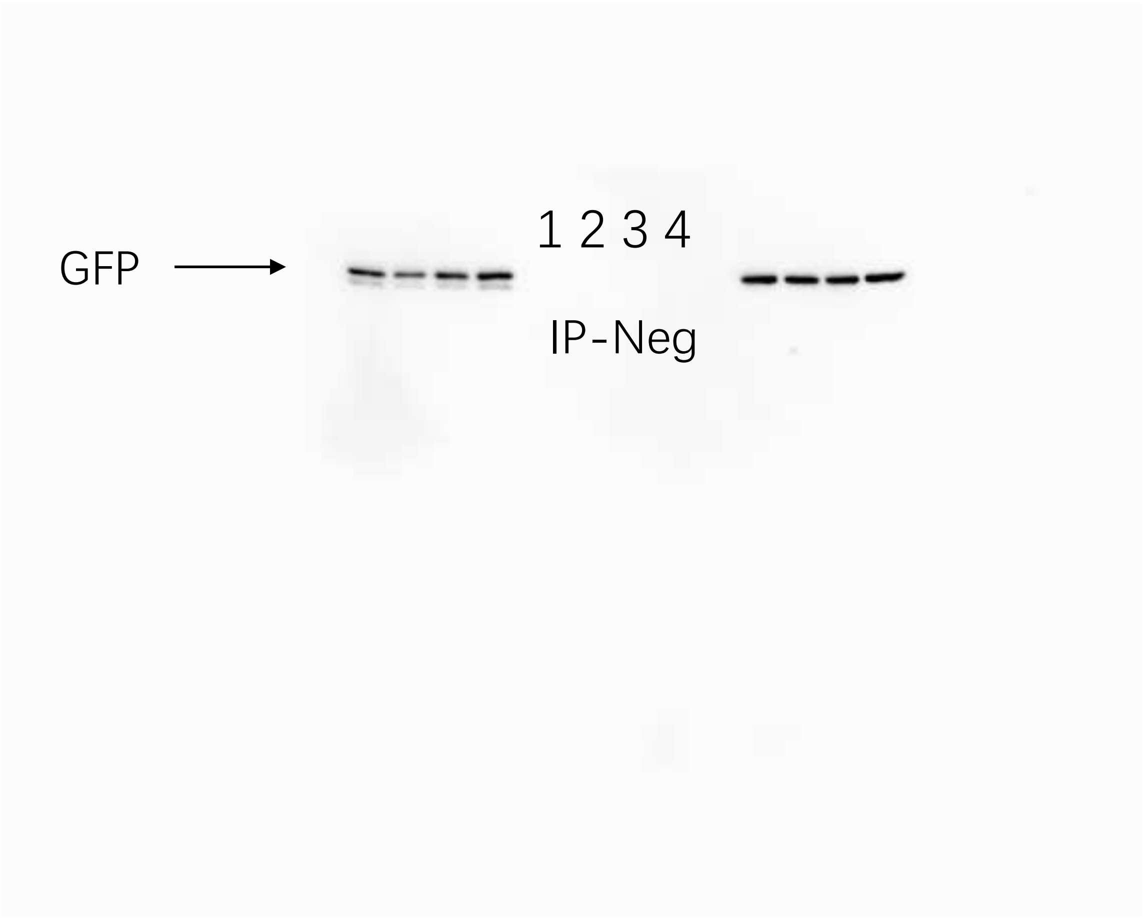

Full and uncropped western blot for Figure 5A Lanes 1-4 are on the figure

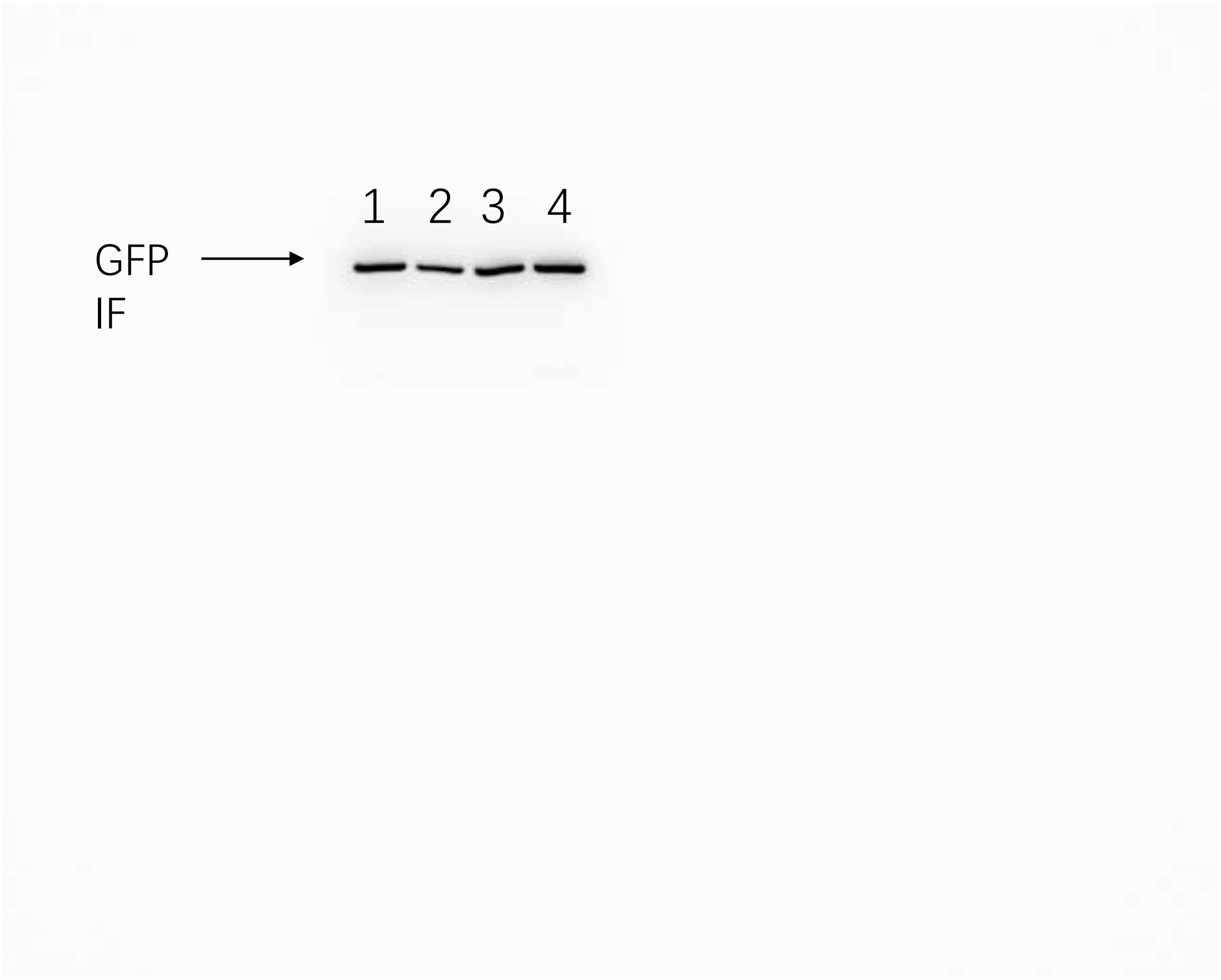

Full and uncropped western blot for Figure 6B Lanes 1-3 are on the figure

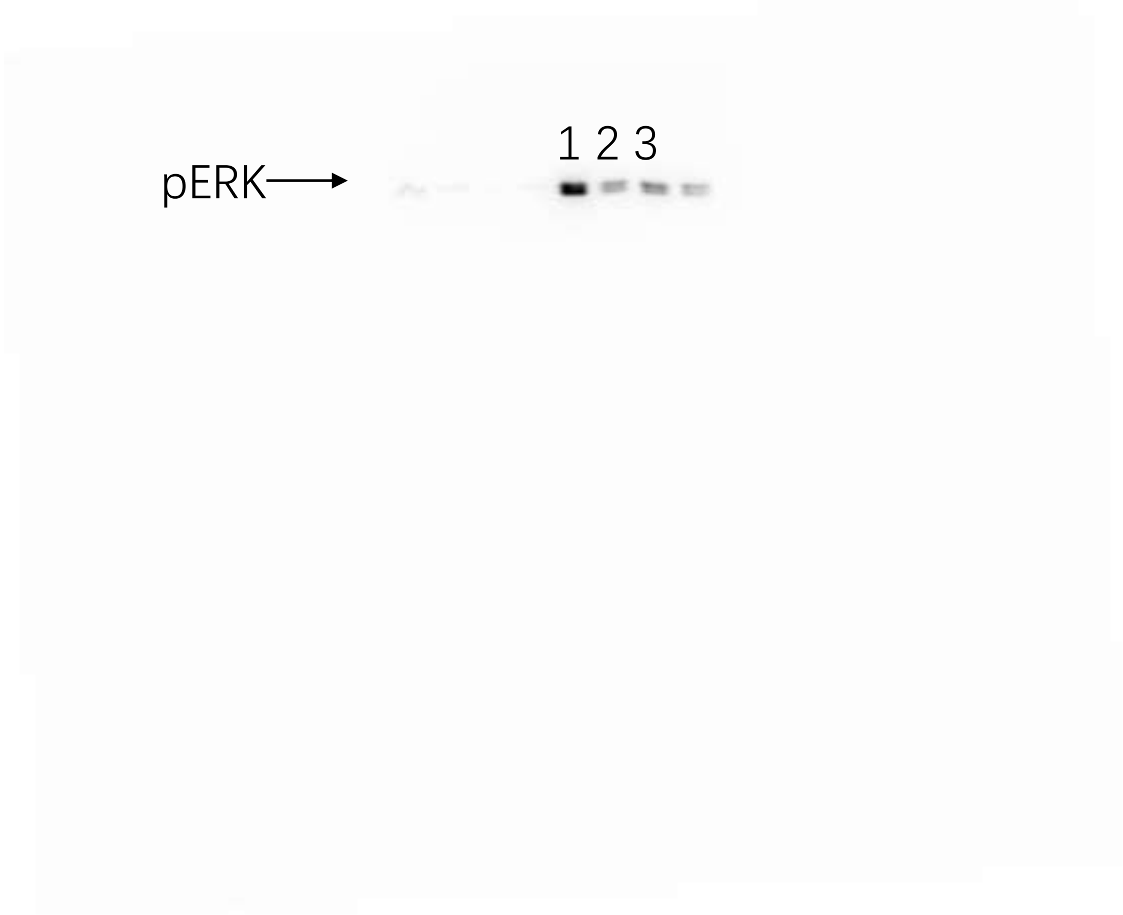

Full and uncropped western blot for Figure 6B Lanes 1-3 are on the figure

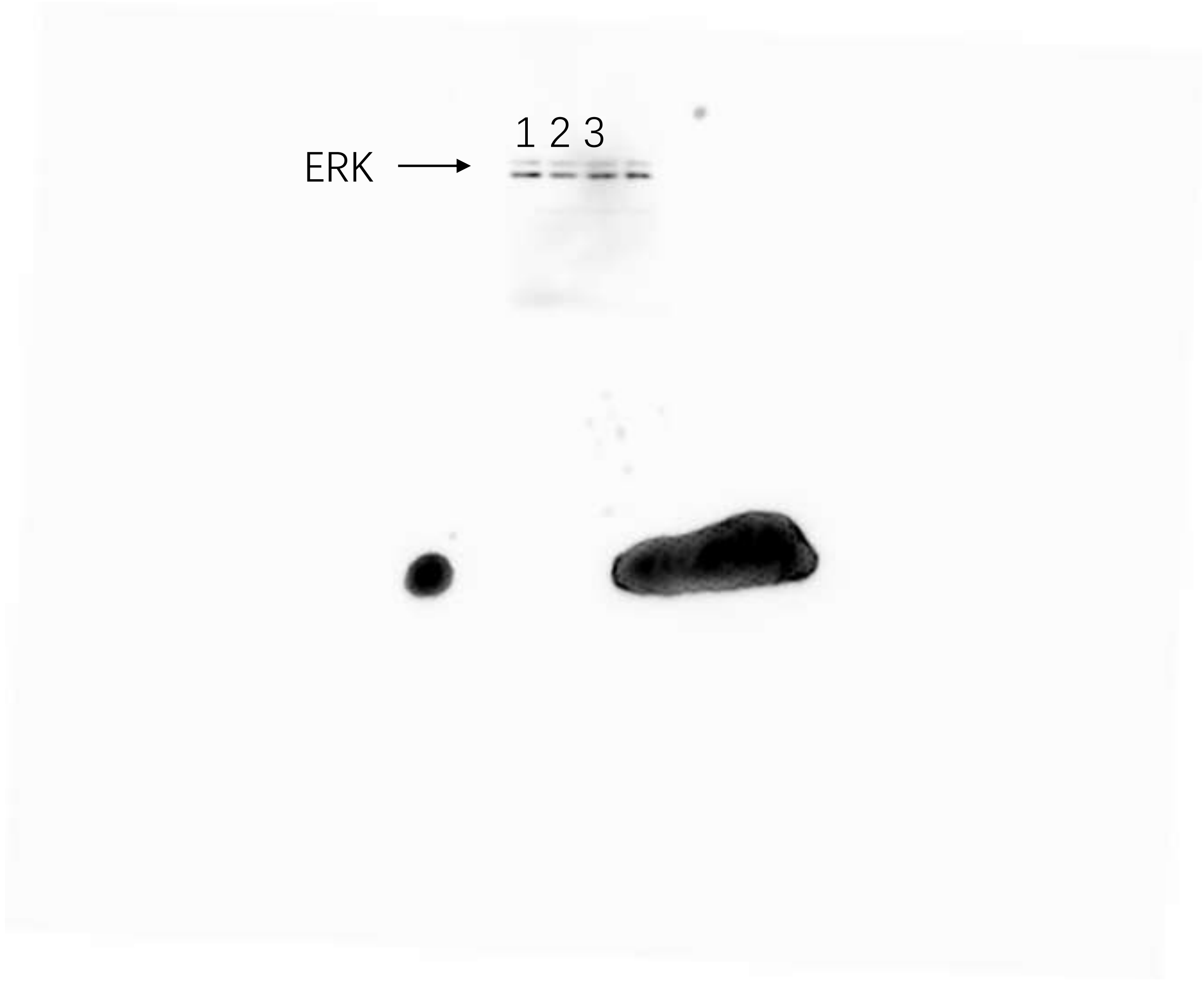

Full and uncropped western blot for Figure 6B Lanes 1-3 are on the figure

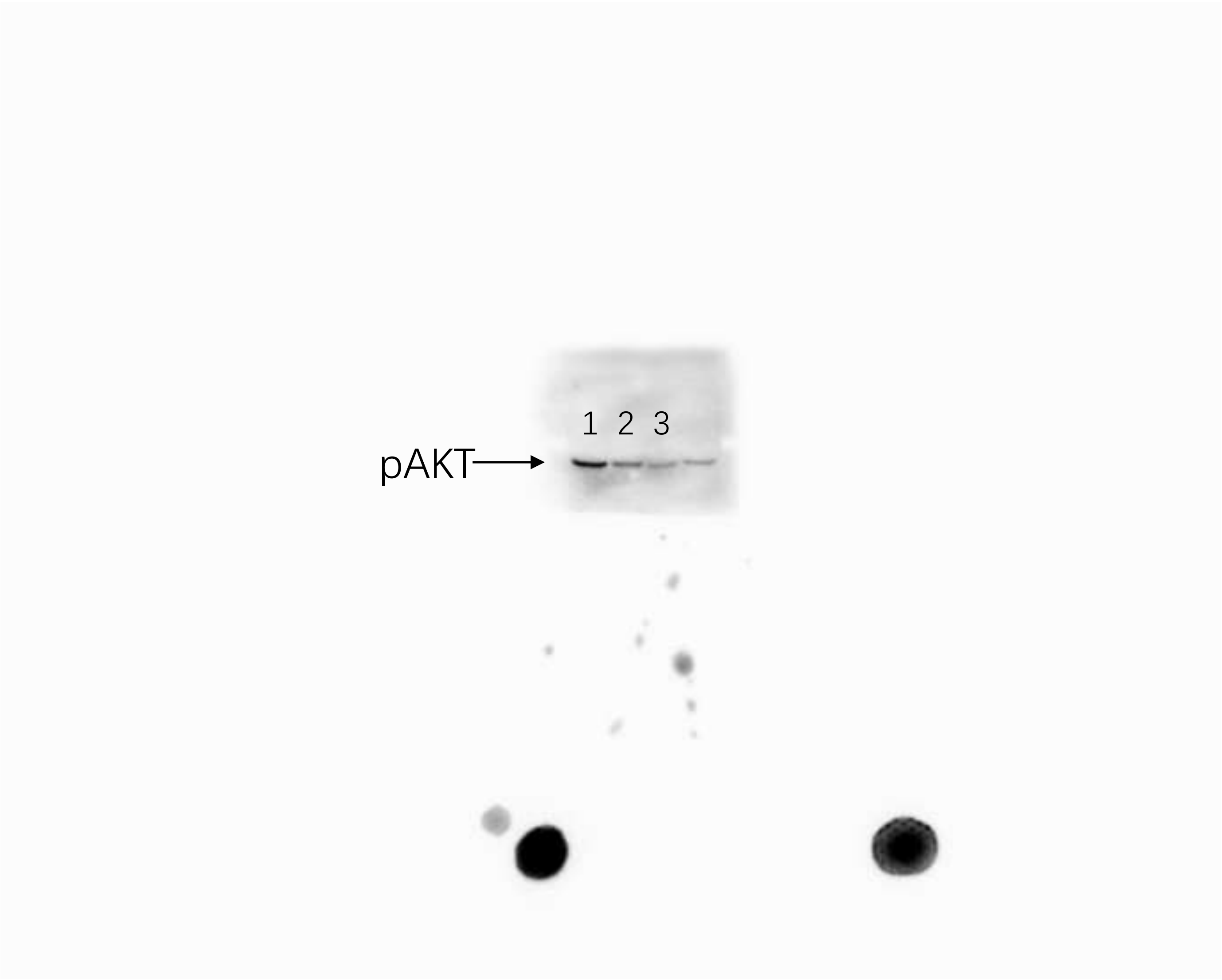

Full and uncropped western blot for Figure 6B Lanes 1-3 are on the figure

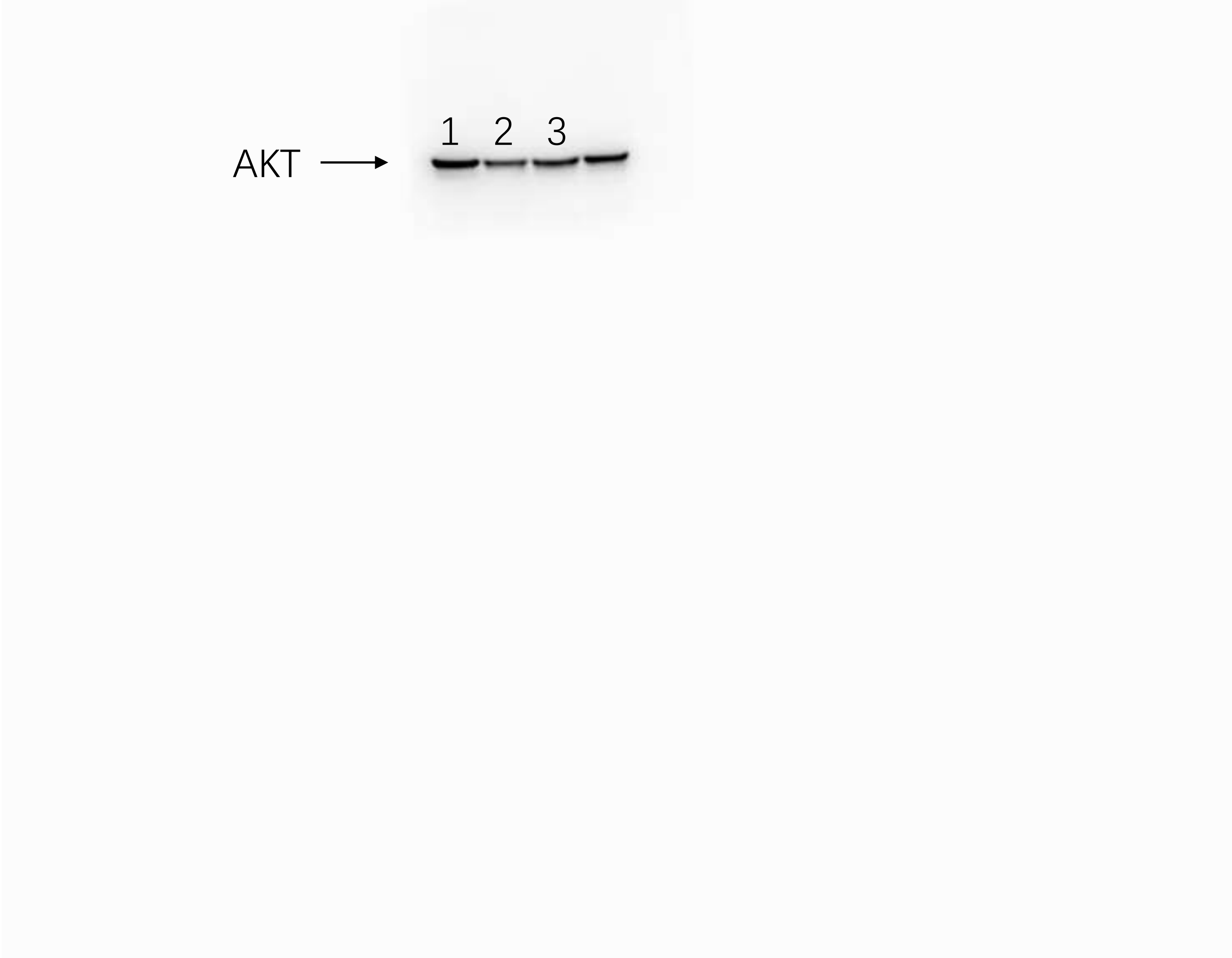

Full and uncropped western blot for Figure 6B Lanes 1-3 are on the figure

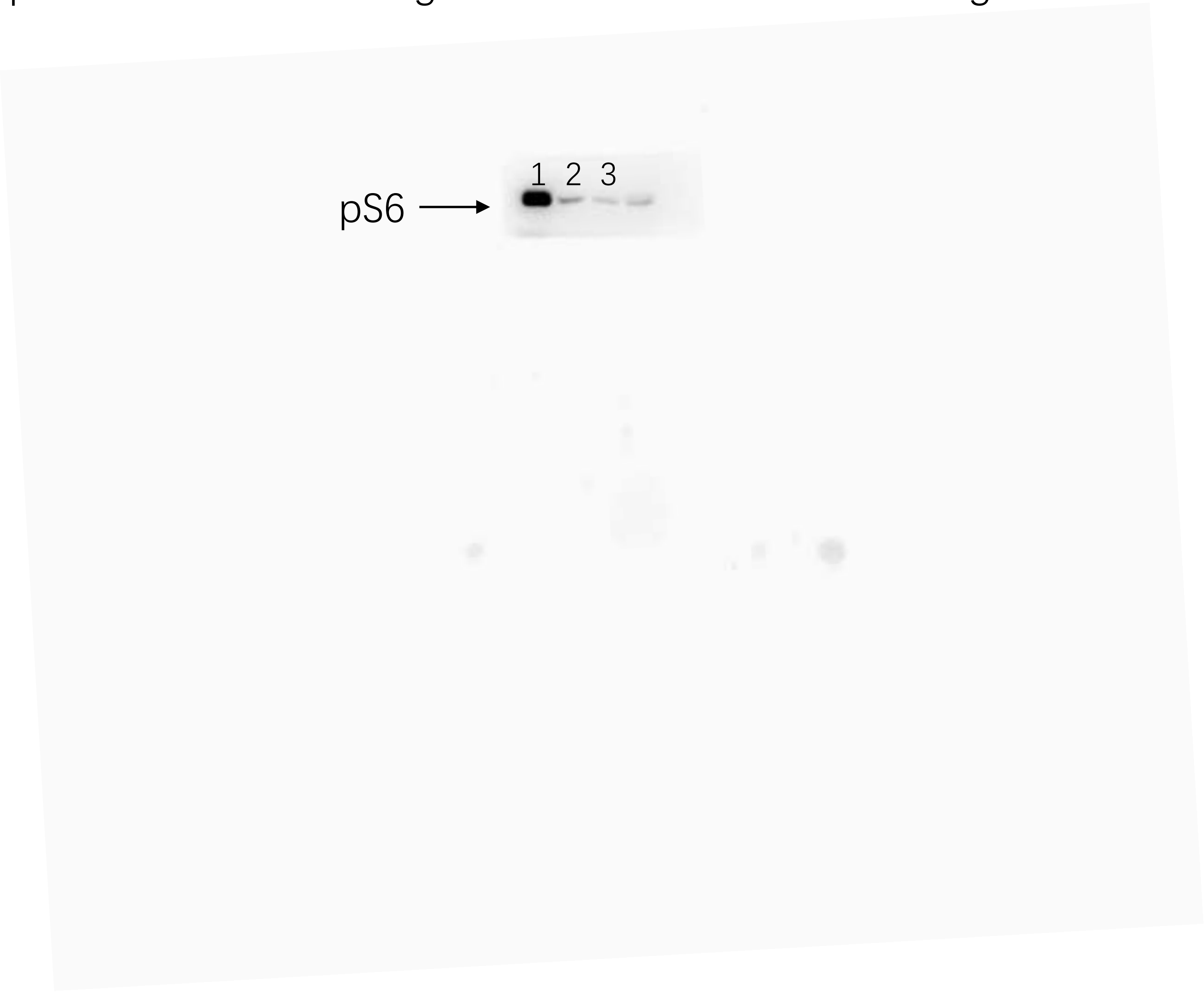

Full and uncropped western blot for Figure 6B Lanes 1-3 are on the figure

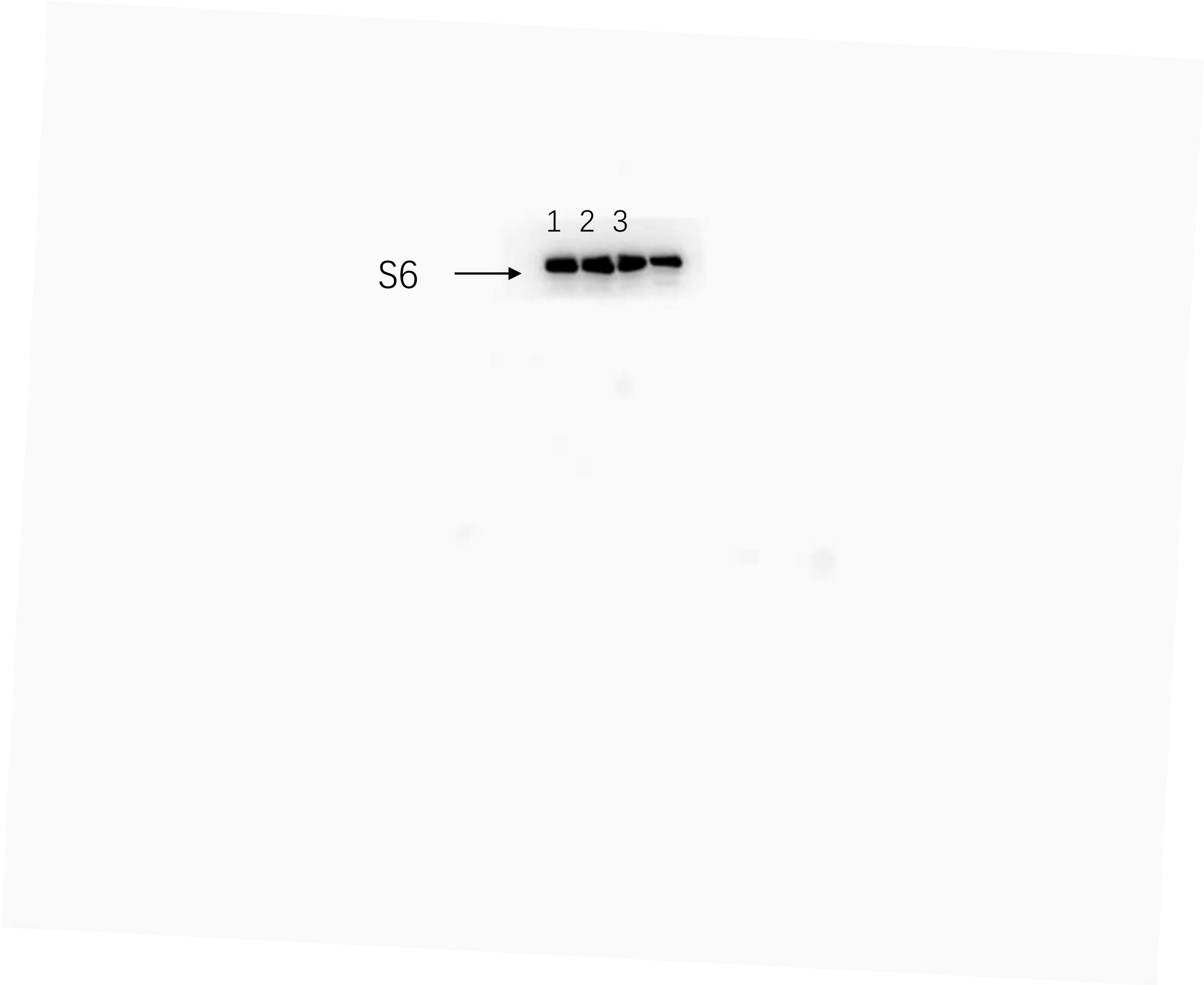

Full and uncropped western blot for Figure 6B Lanes 1-3 are on the figure

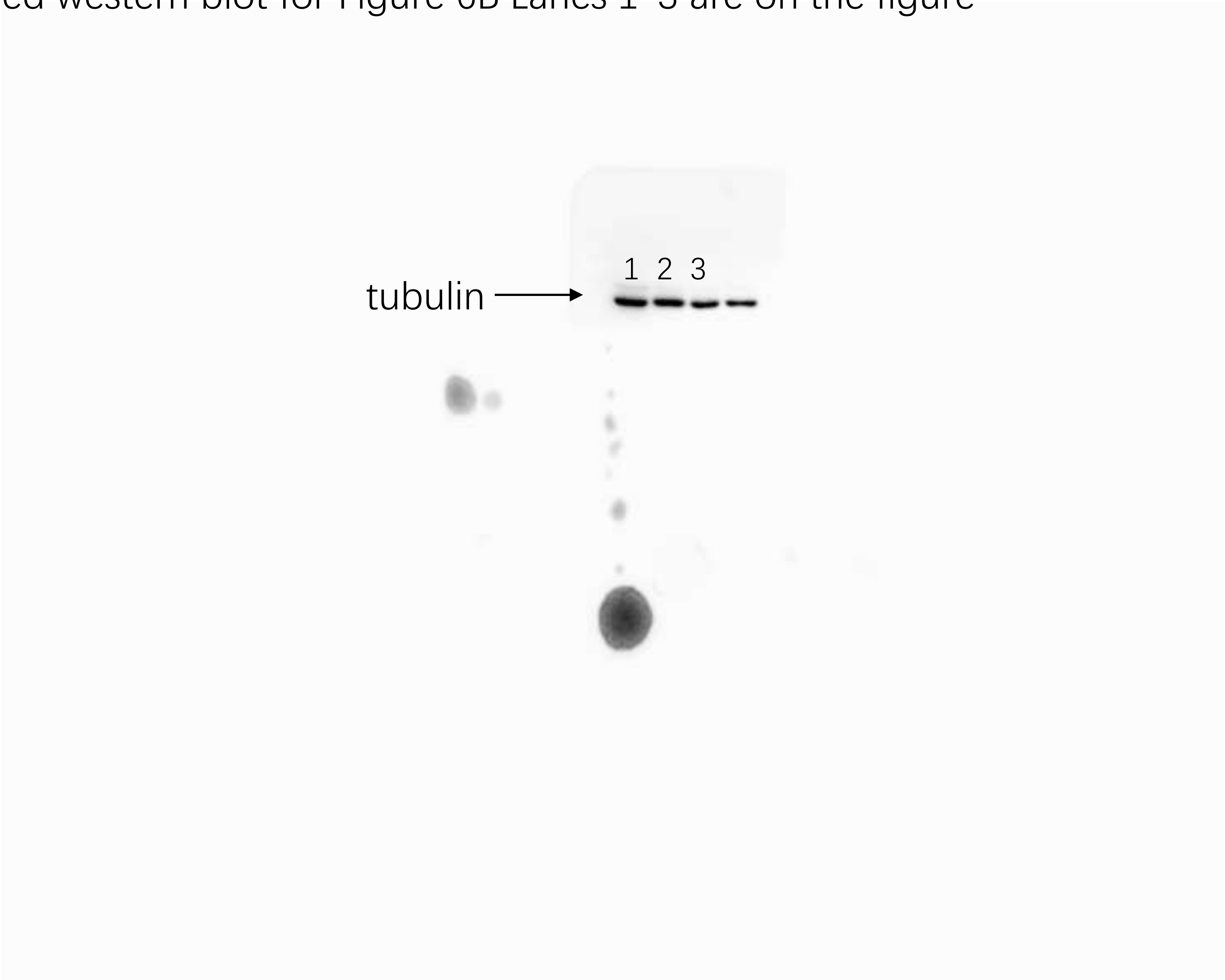

Full and uncropped western blot for Figure 6B Lanes 4-6 are on the figure

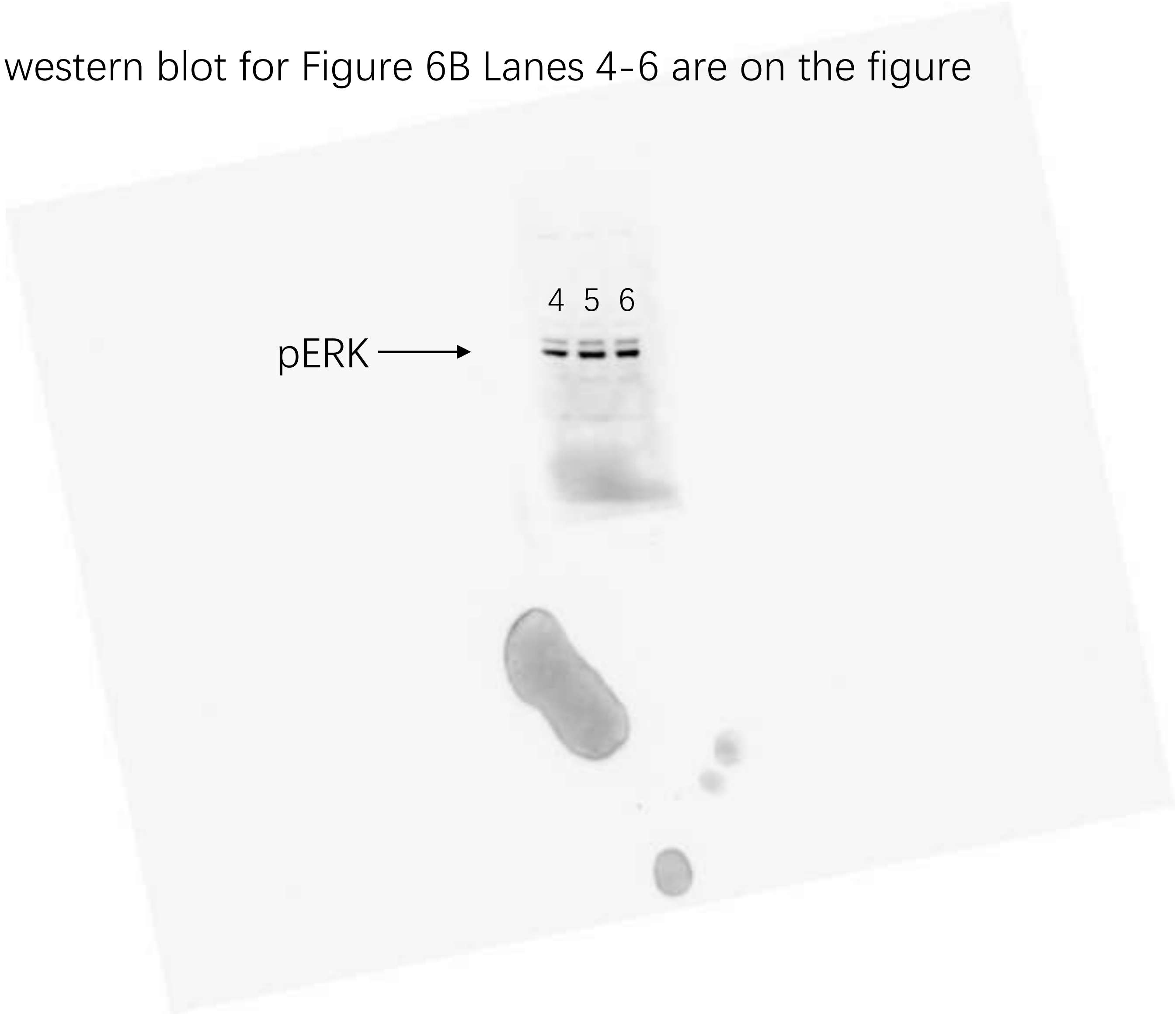

Full and uncropped western blot for Figure 6B Lanes 4-6 are on the figure

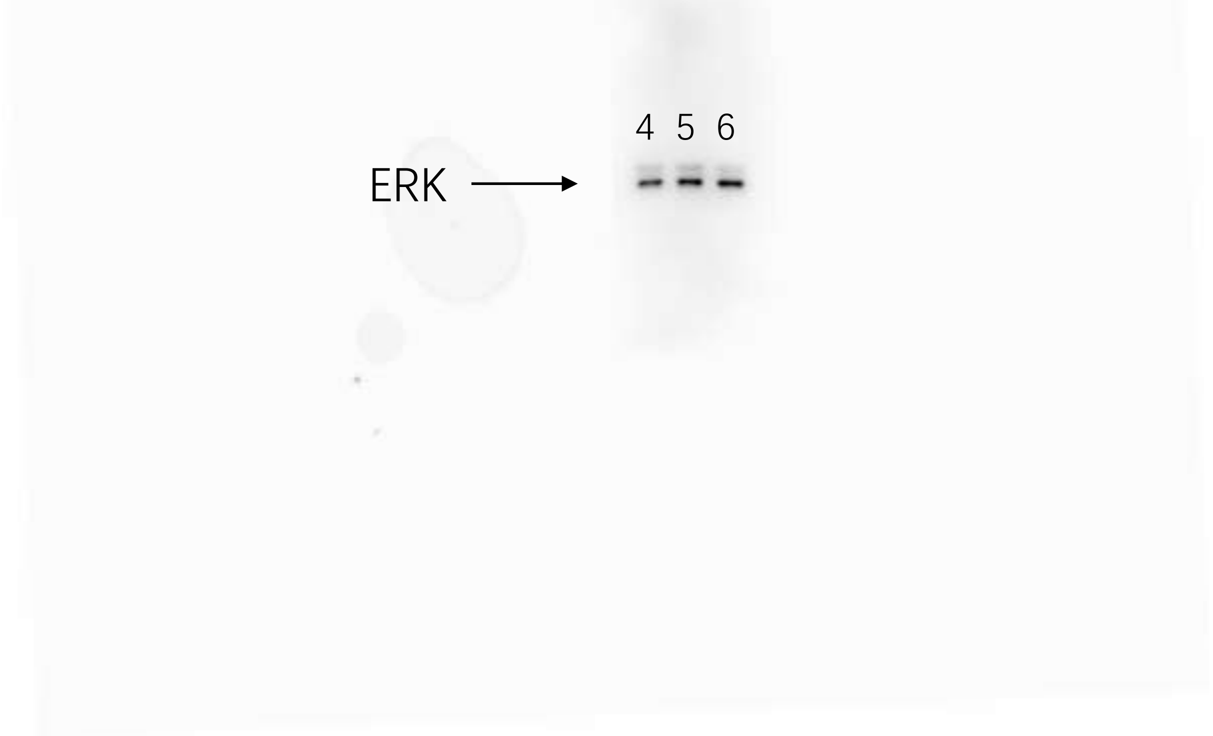

Full and uncropped western blot for Figure 6B Lanes 4-6 are on the figure

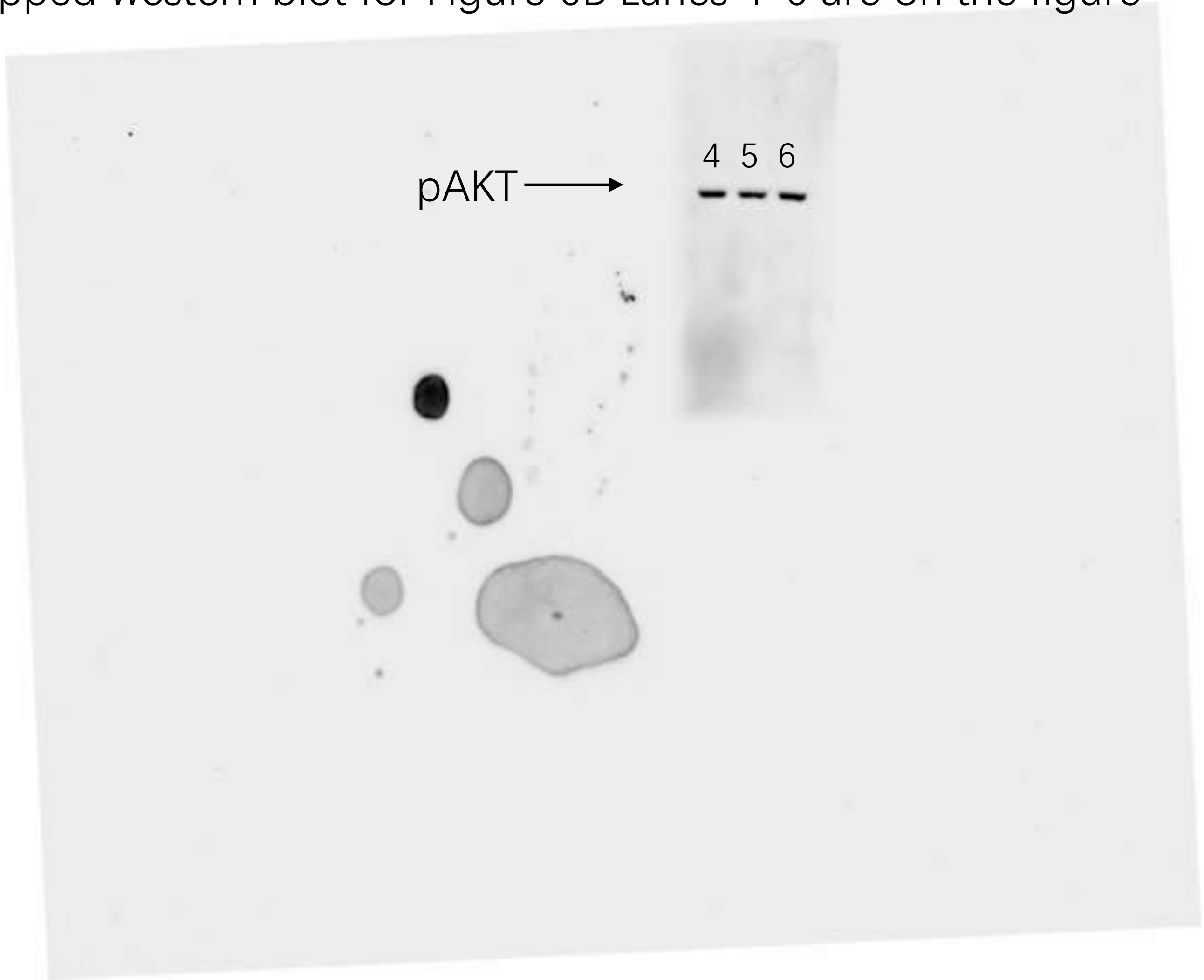

Full and uncropped western blot for Figure 6B Lanes 4-6 are on the figure

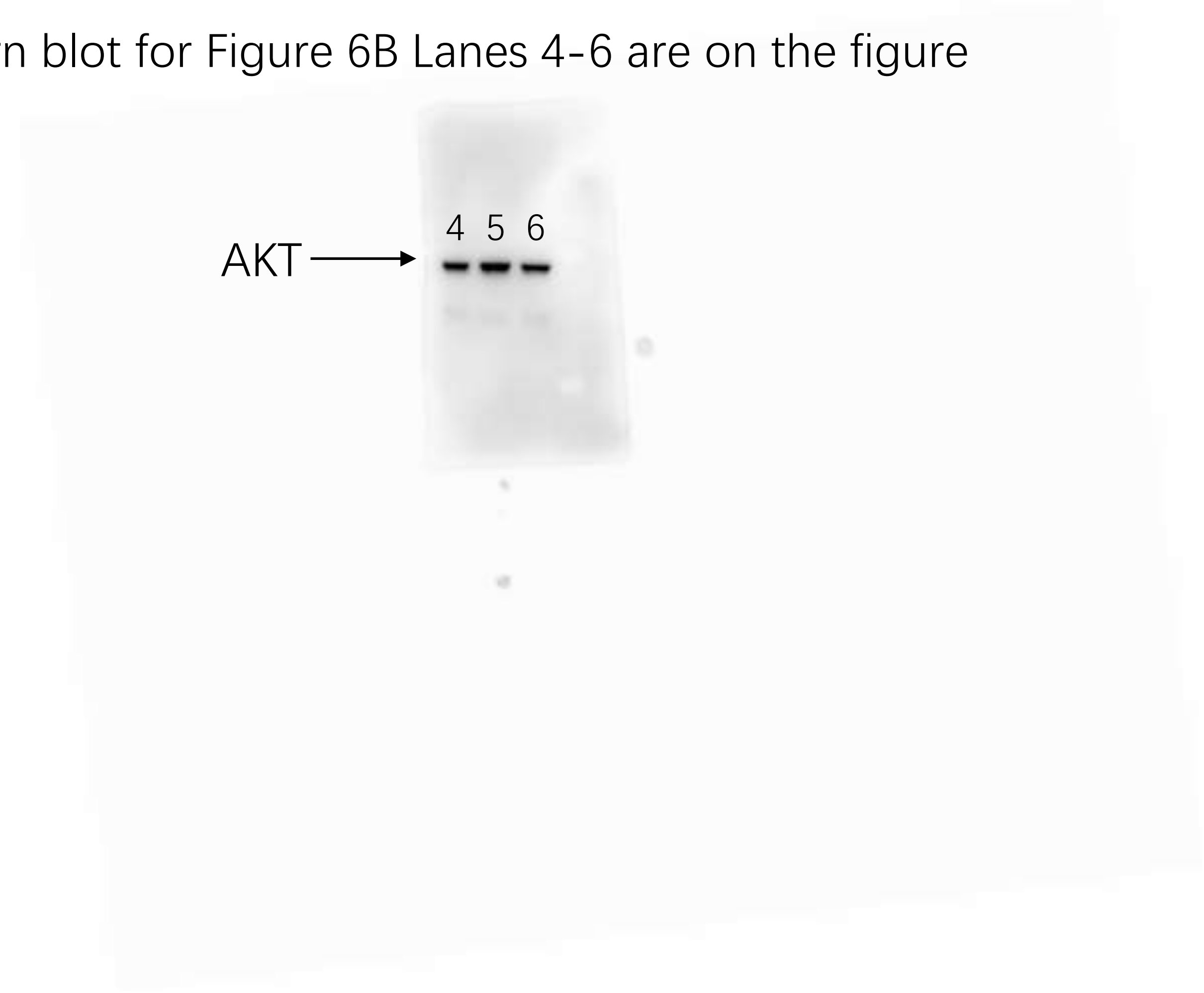

Full and uncropped western blot for Figure 6B Lanes 4-6 are on the figure

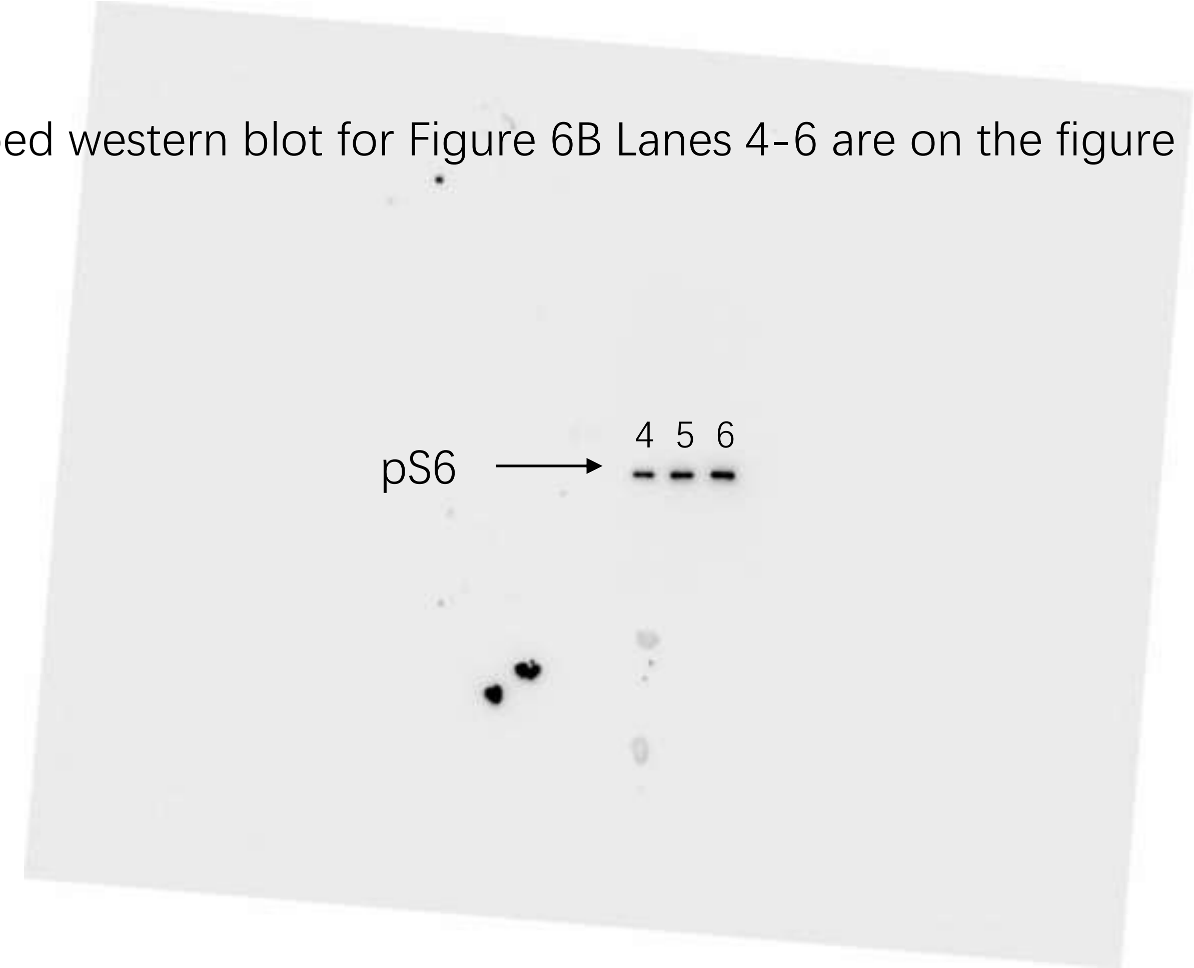

Full and uncropped western blot for Figure 6B Lanes 4-6 are on the figure

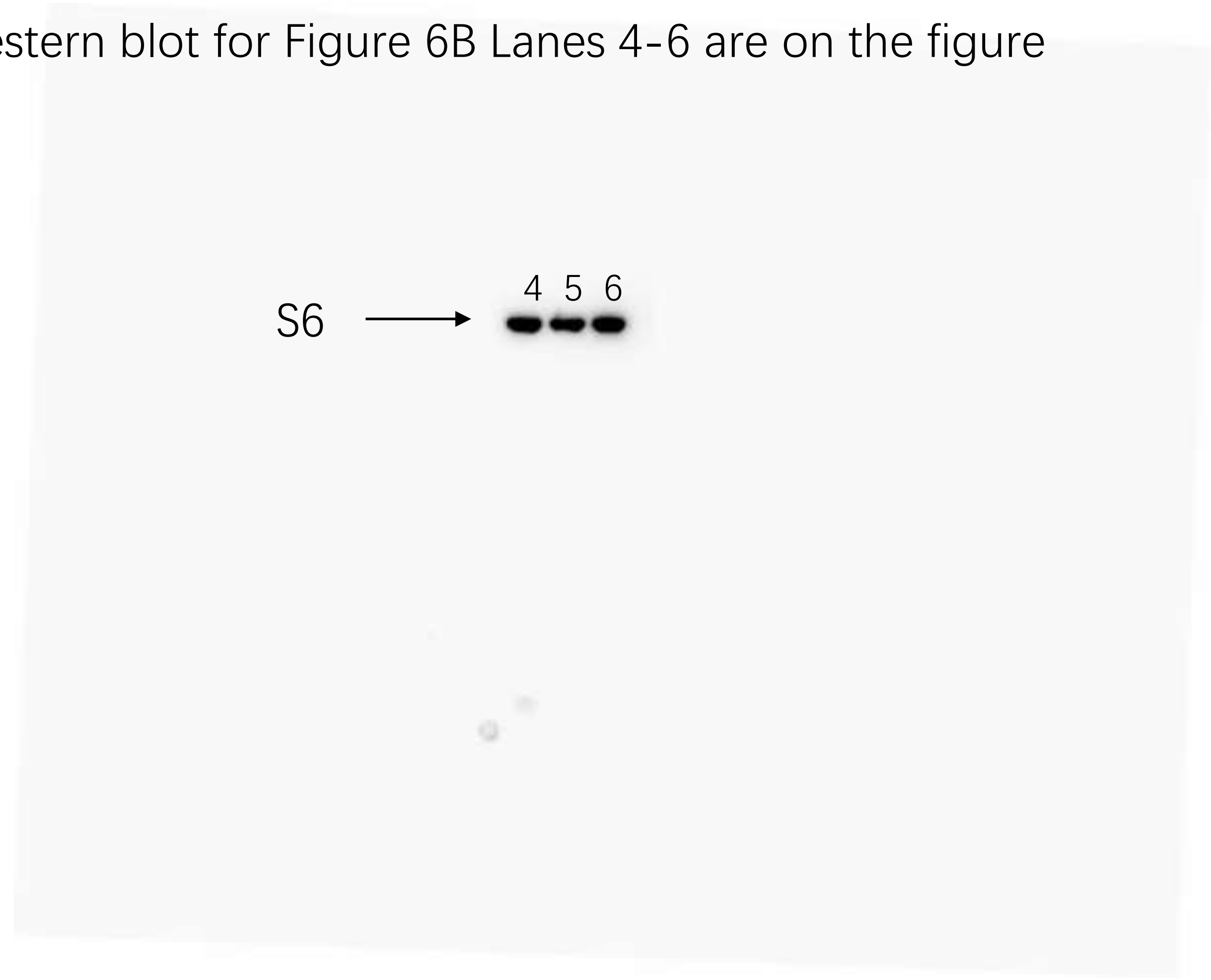

Full and uncropped western blot for Figure 6B Lanes 4-6 are on the figure

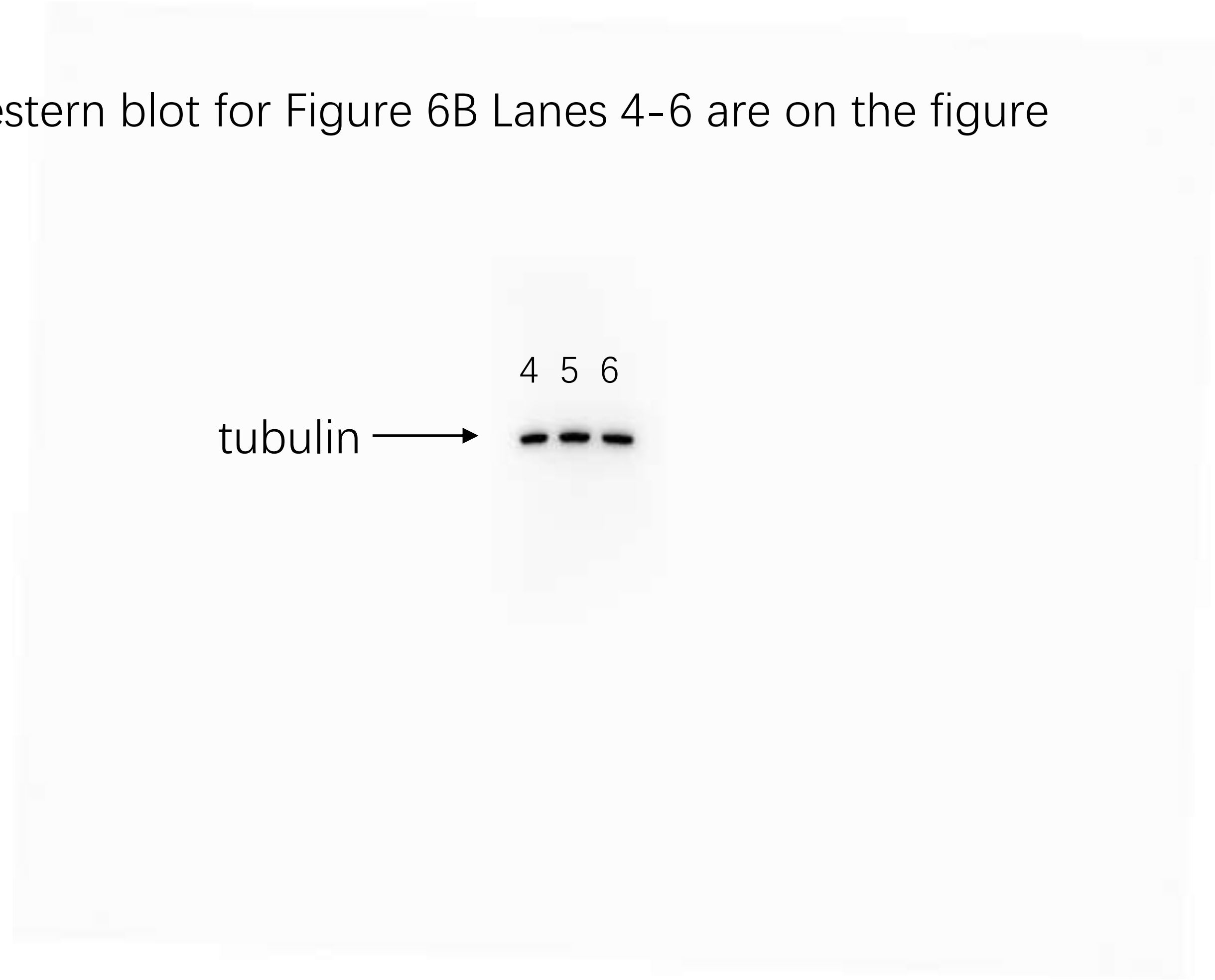

Full and uncropped western blot for Figure 6D Lanes 1-6 are on the figure

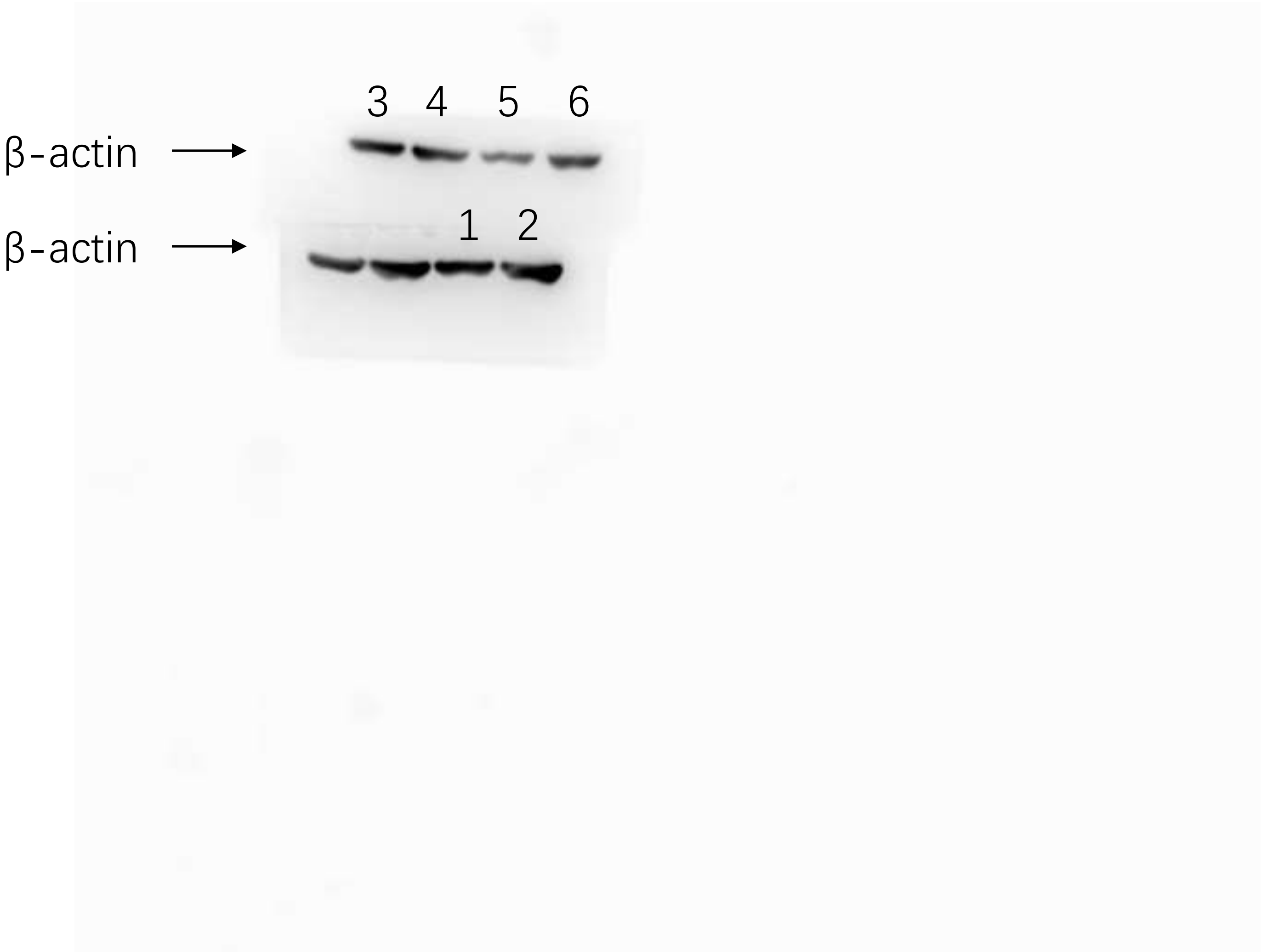

Full and uncropped western blot for Figure 6D Lanes 7-18 are on the figure

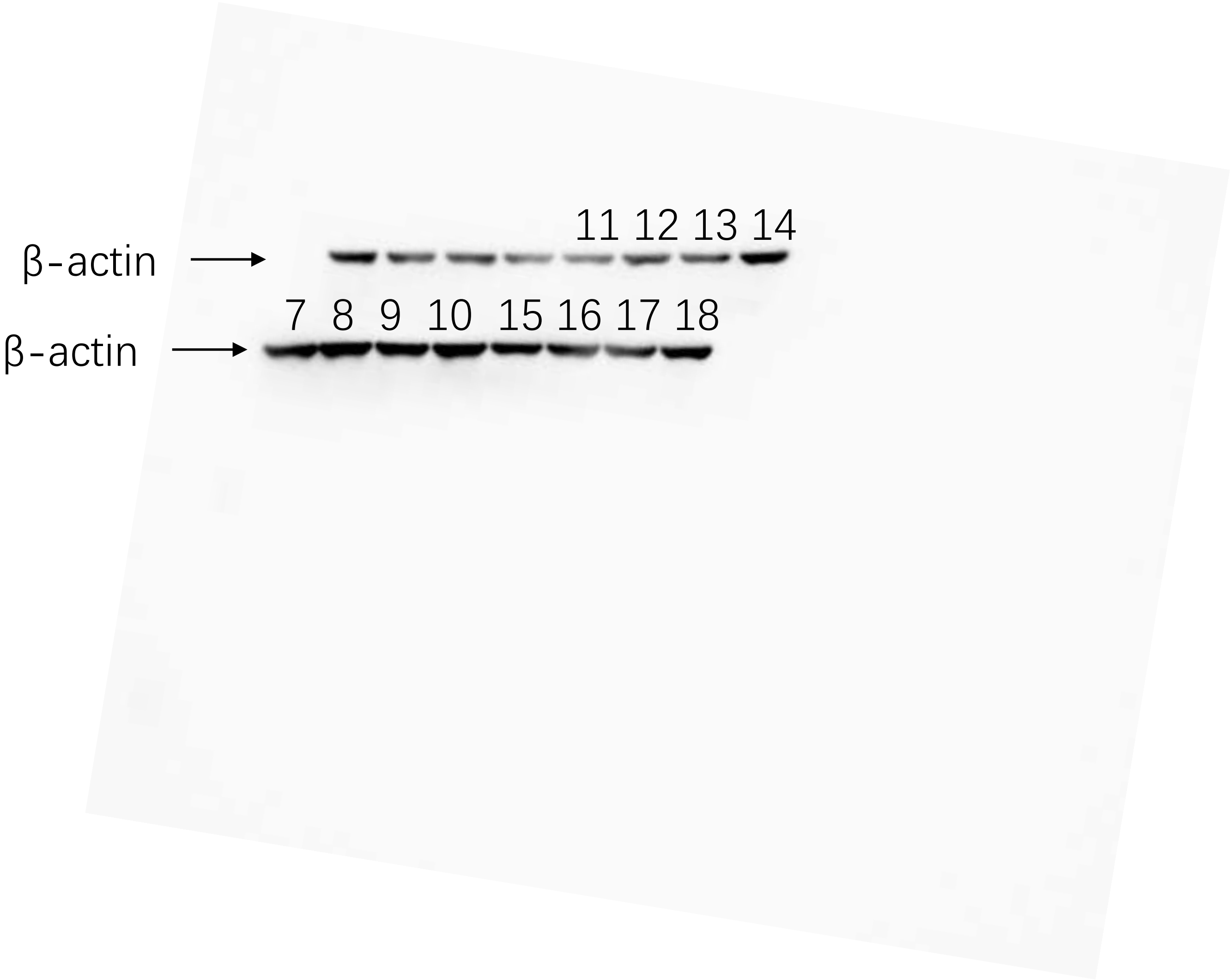

Full and uncropped western blot for Figure 6D Lanes 1-10 are on the figure

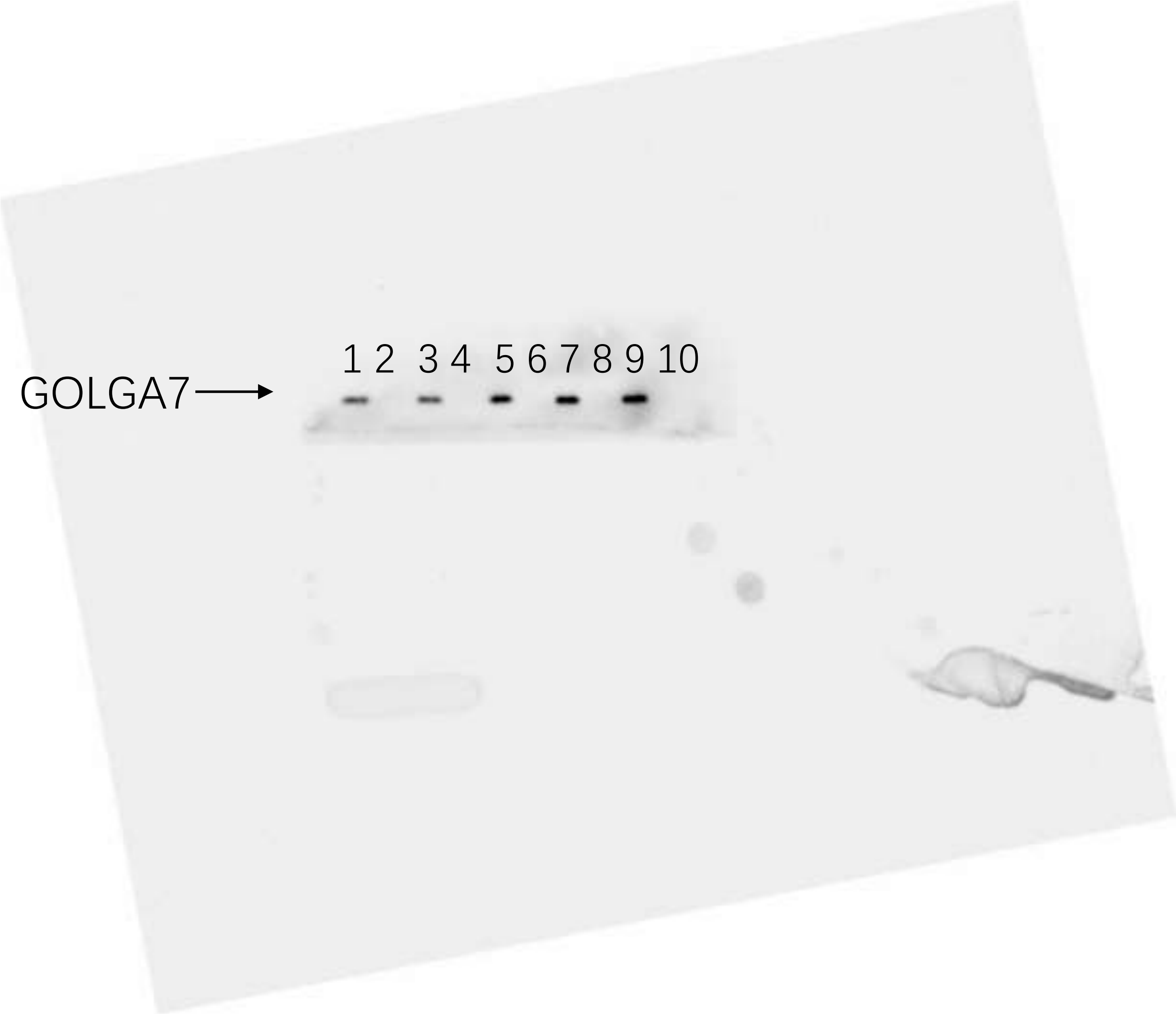

Full and uncropped western blot for Figure 6D Lanes 11-18 are on the figure

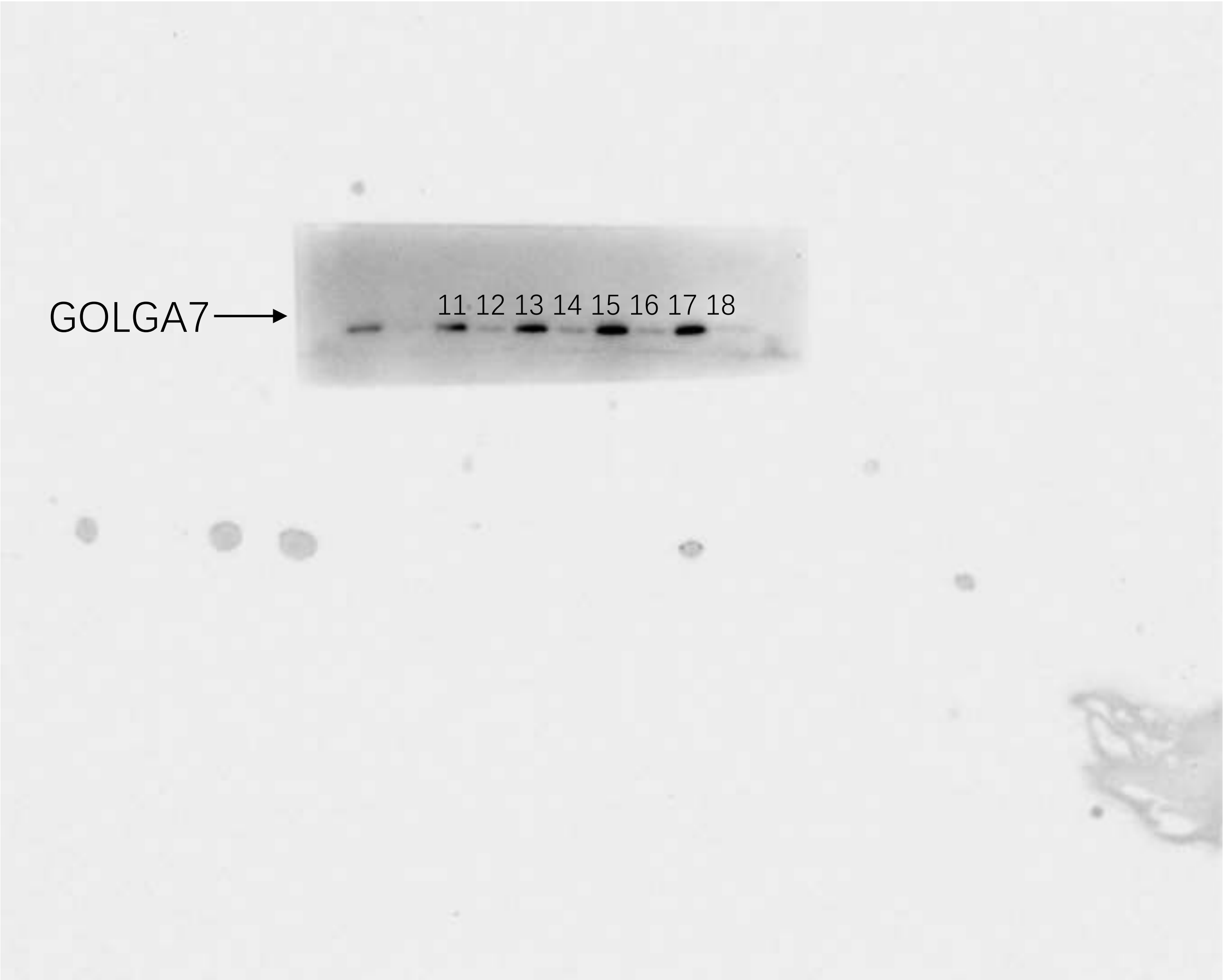

Full and uncropped western blot for Figure 6D Lanes 1, 2, 11-14 are on the figure

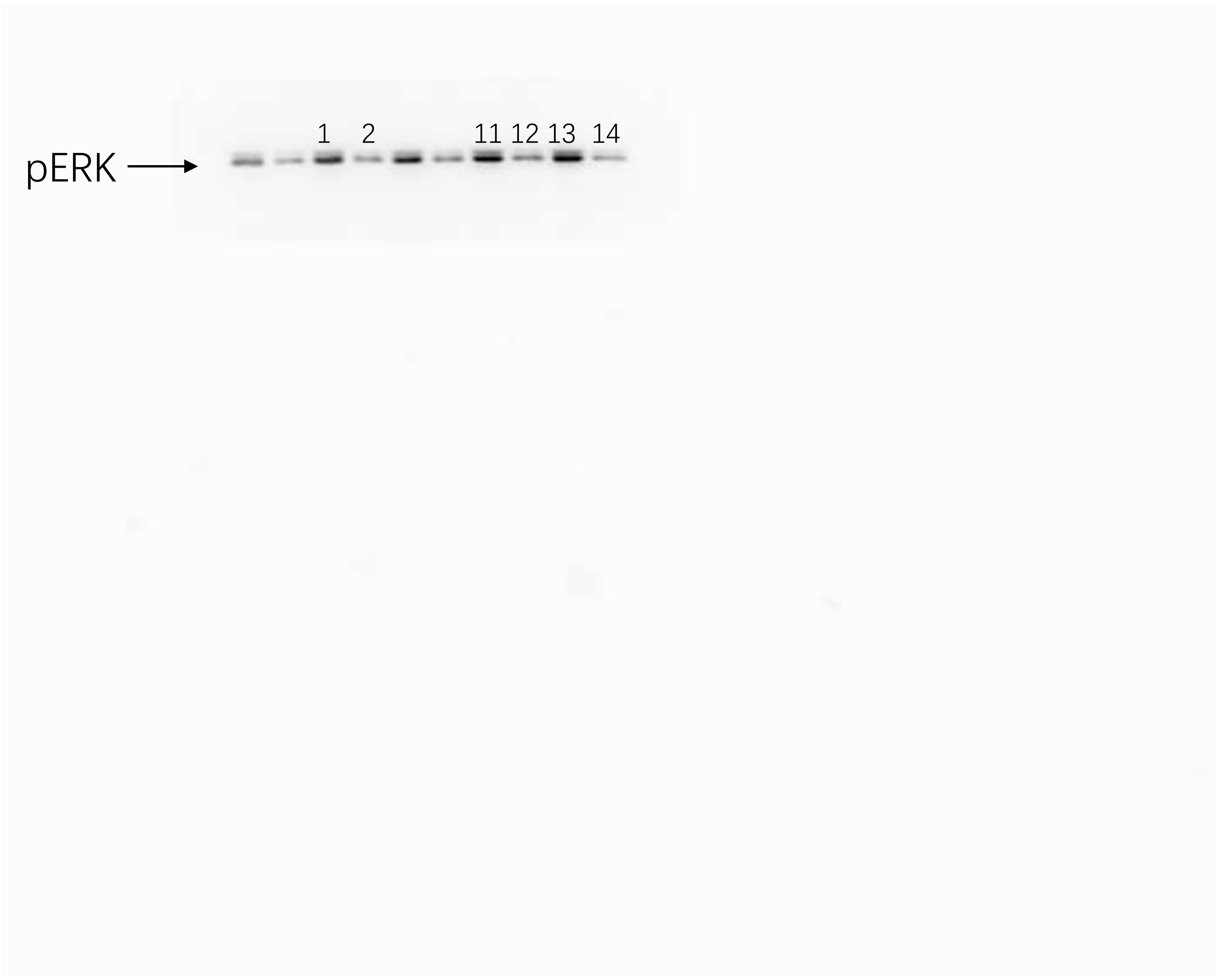

Full and uncropped western blot for Figure 6D Lanes 3-6 are on the figure

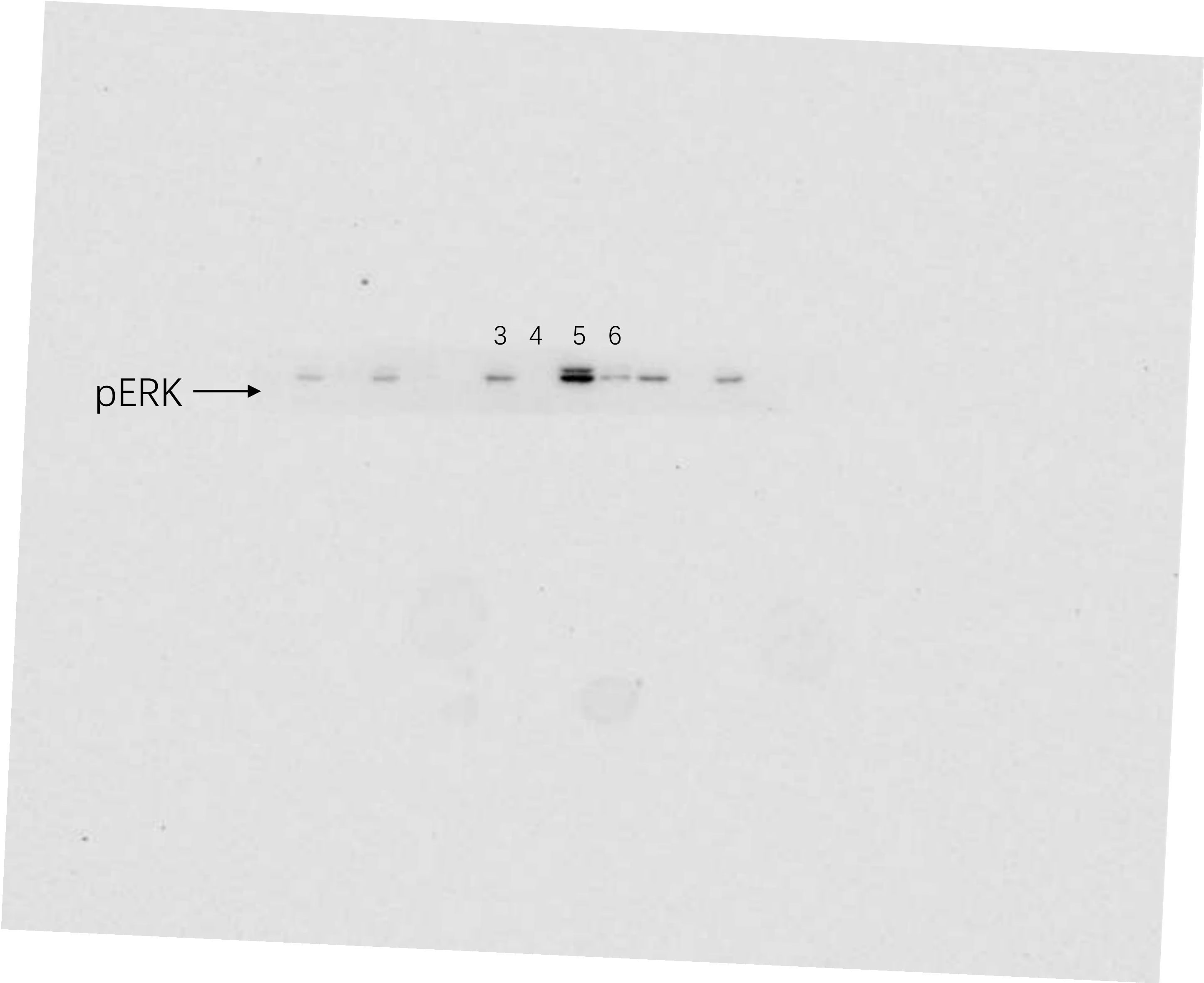

Full and uncropped western blot for Figure 6D Lanes 7-10 are on the figure

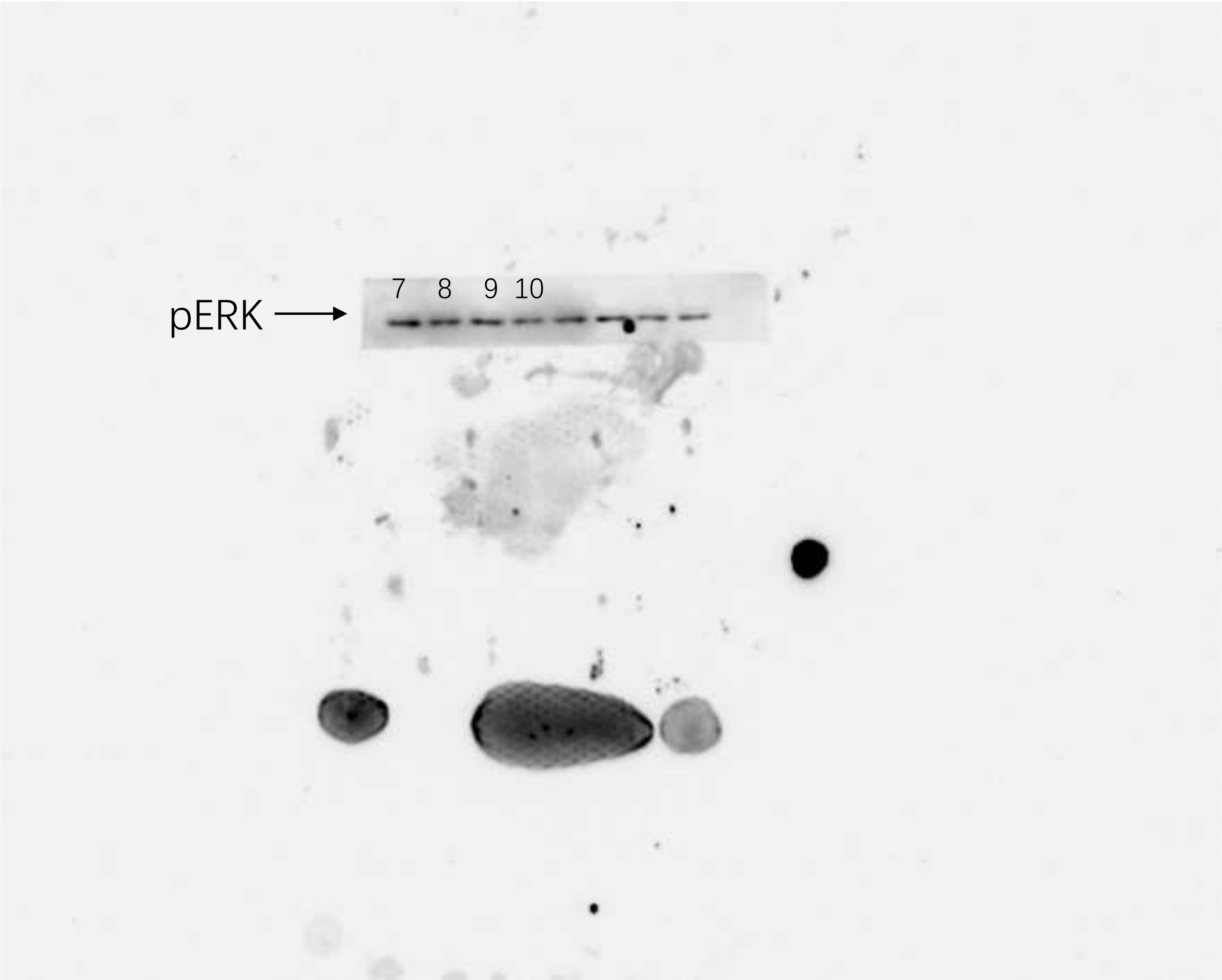

Full and uncropped western blot for Figure 6D Lanes 15-18 are on the figure

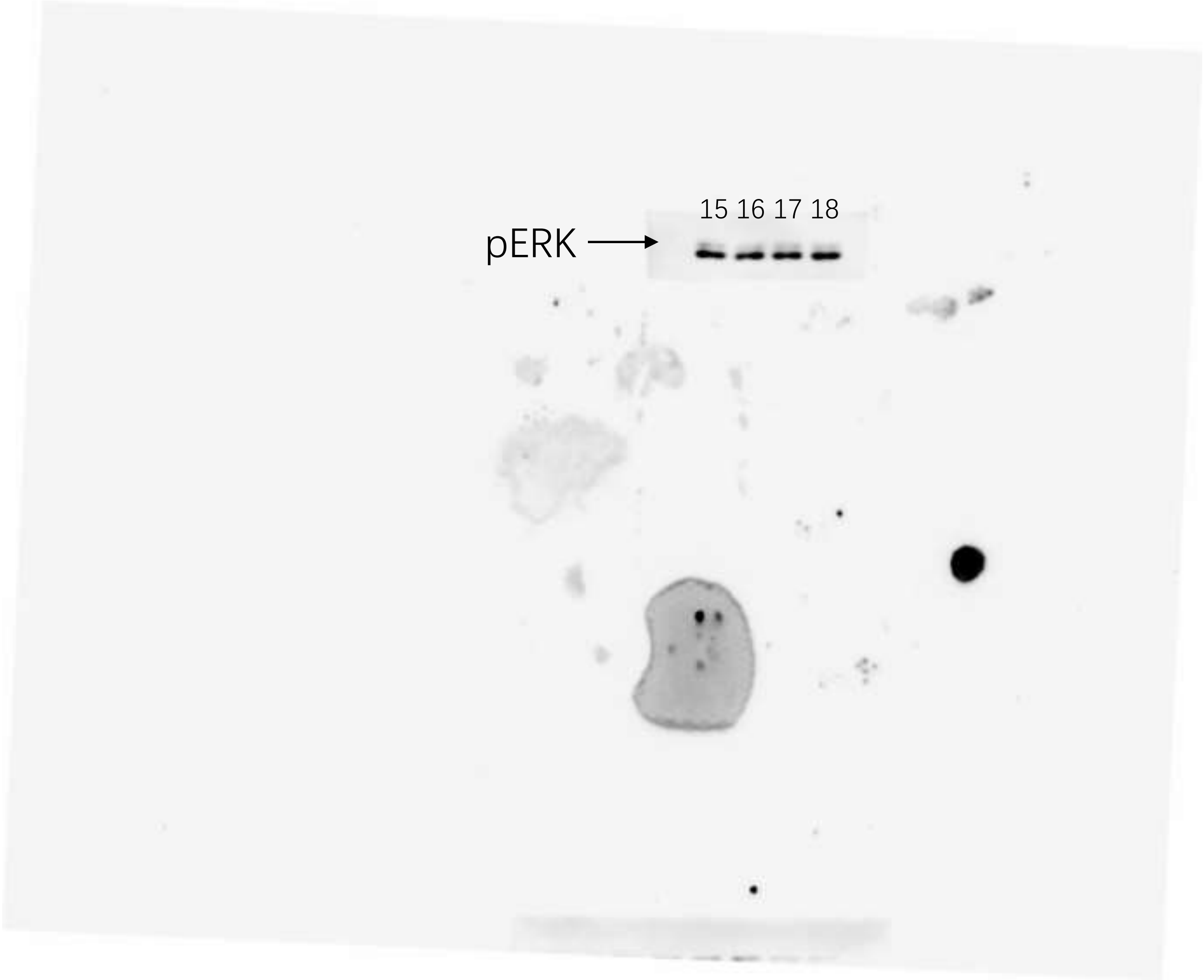

Full and uncropped western blot for Figure 6D Lanes 1, 2 are on the figure

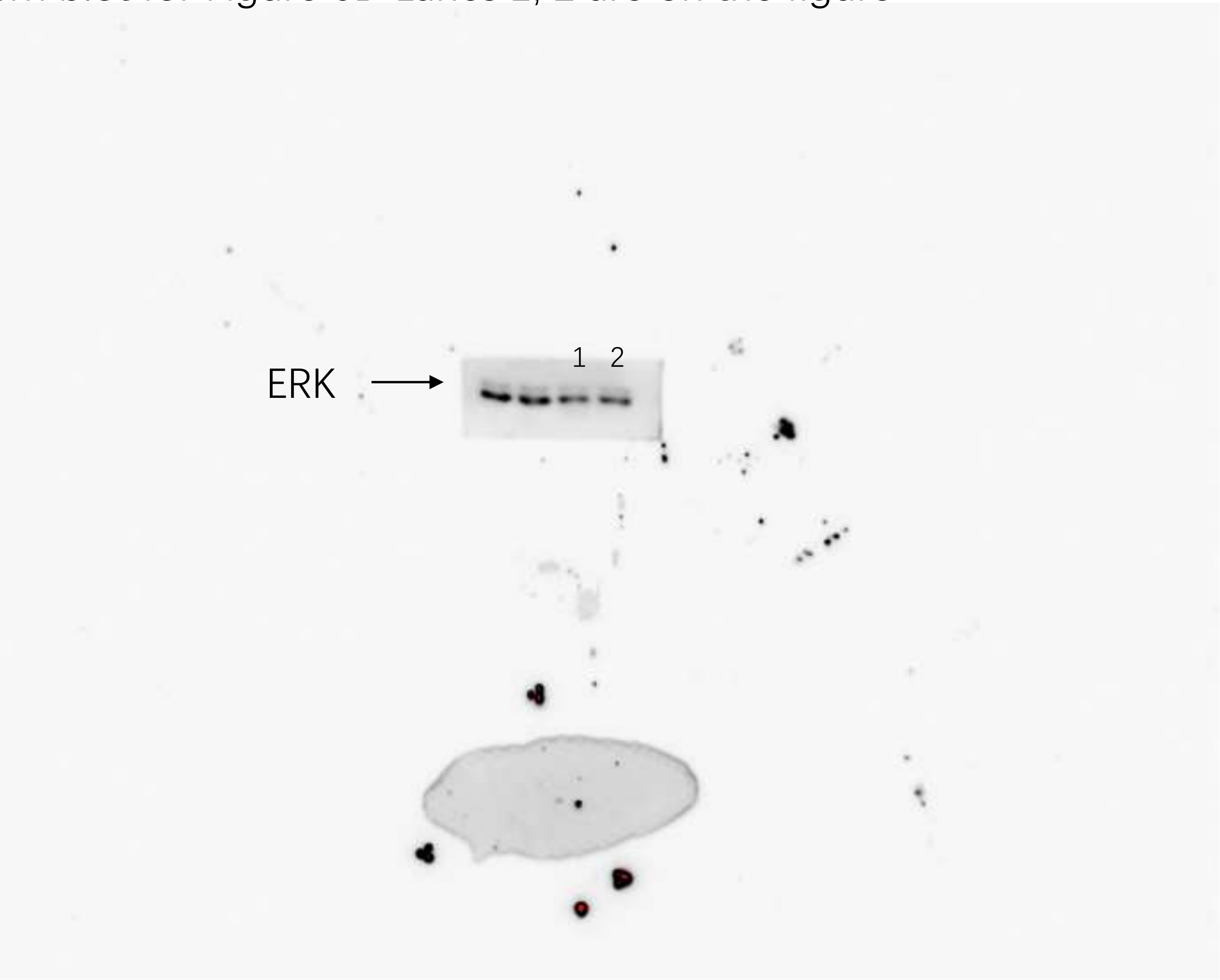

Full and uncropped western blot for Figure 6D Lanes 3-6, 11-14 are on the figure

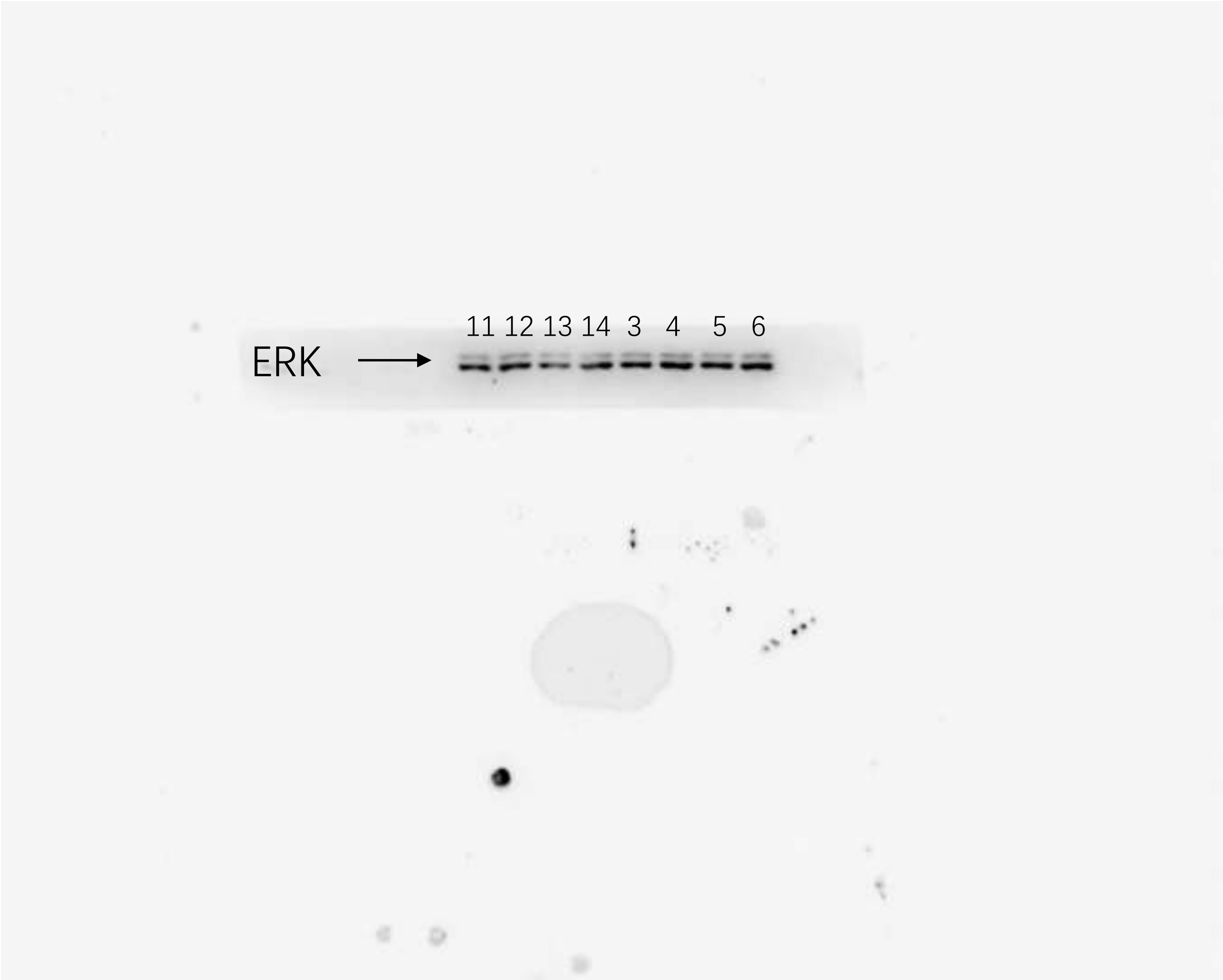

Full and uncropped western blot for Figure 6D Lanes 7-10 are on the figure

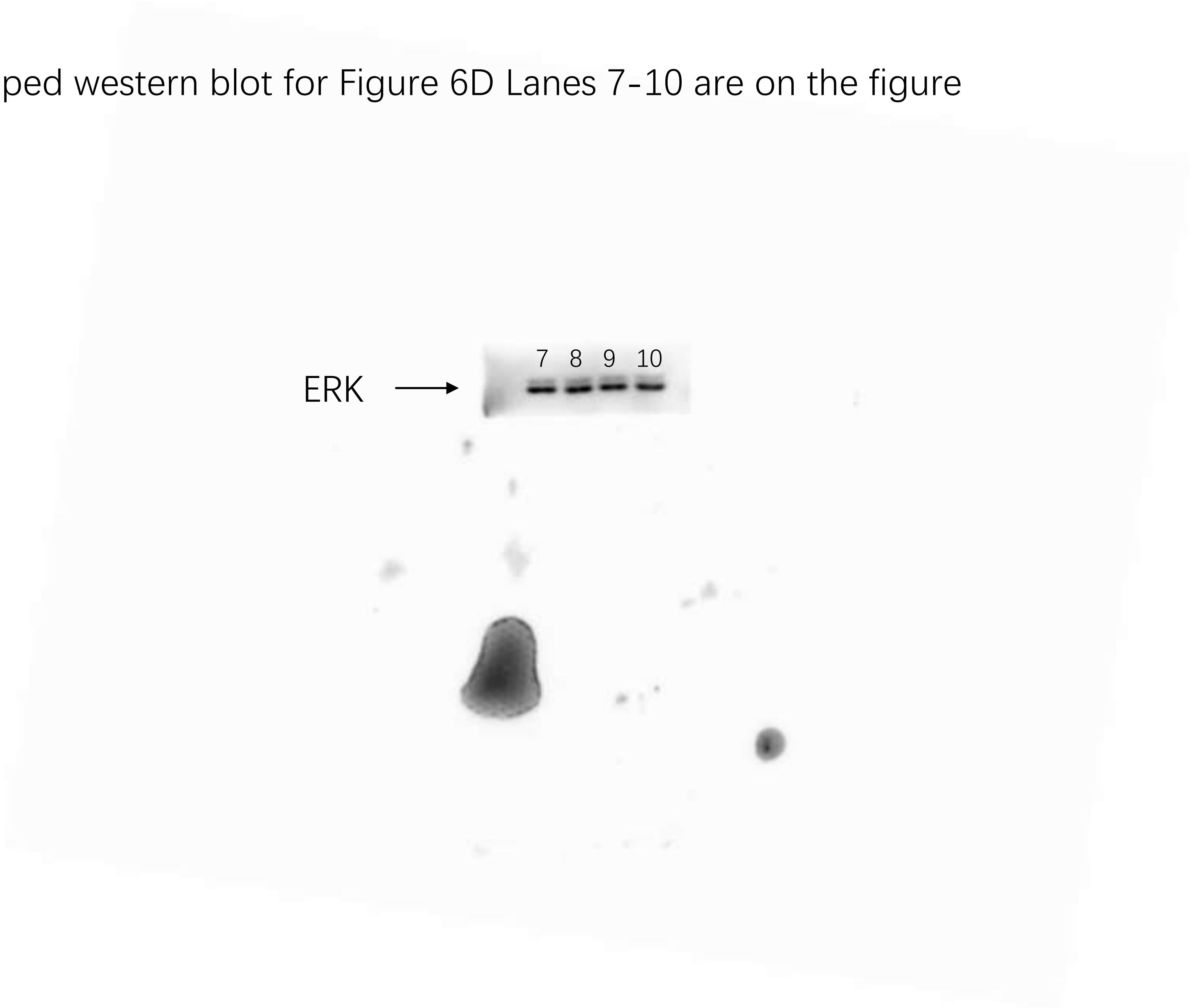

Full and uncropped western blot for Figure 6D Lanes 15-18 are on the figure

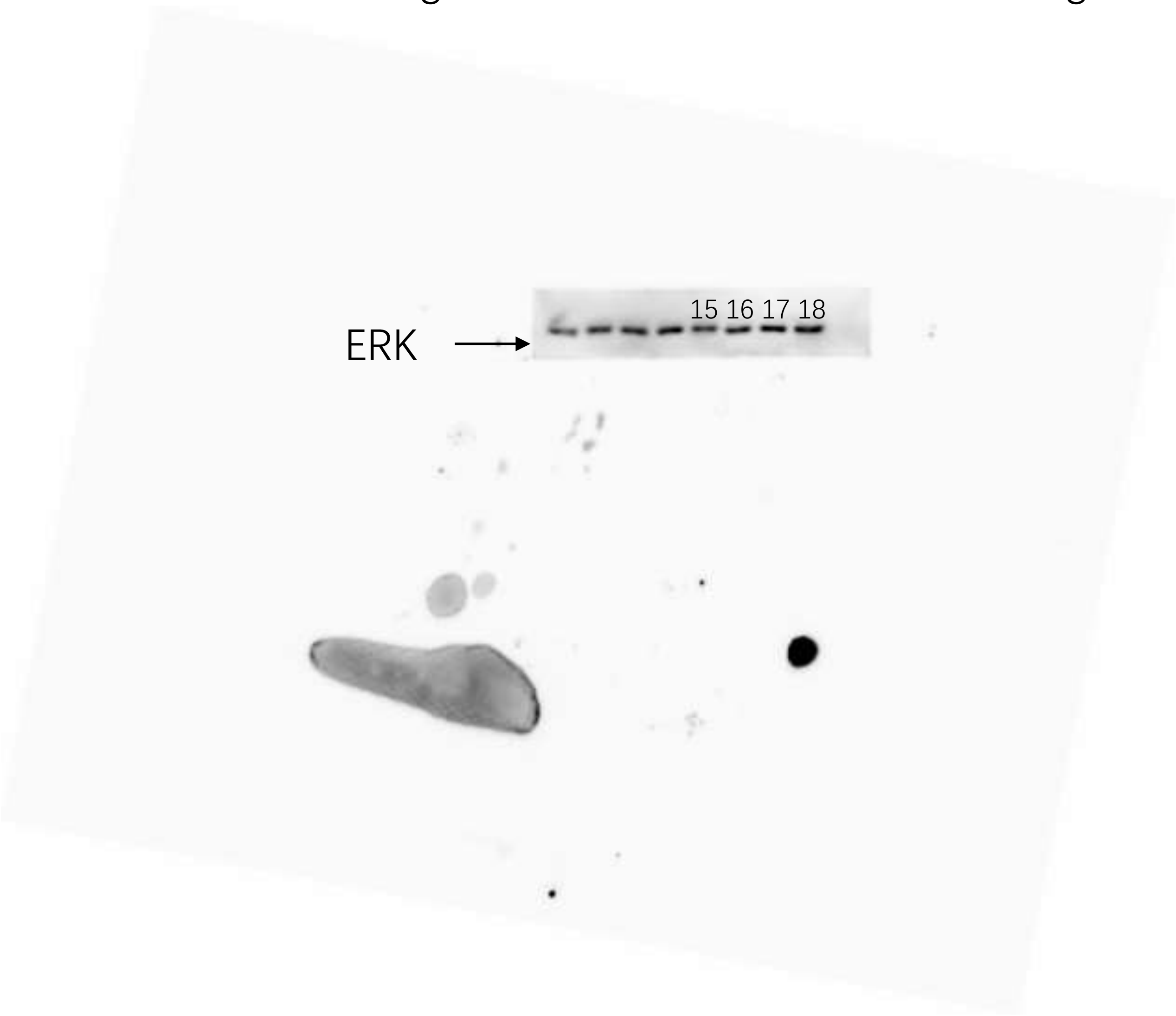

Full and uncropped western blot for Figure 6D Lanes 1-6 are on the figure

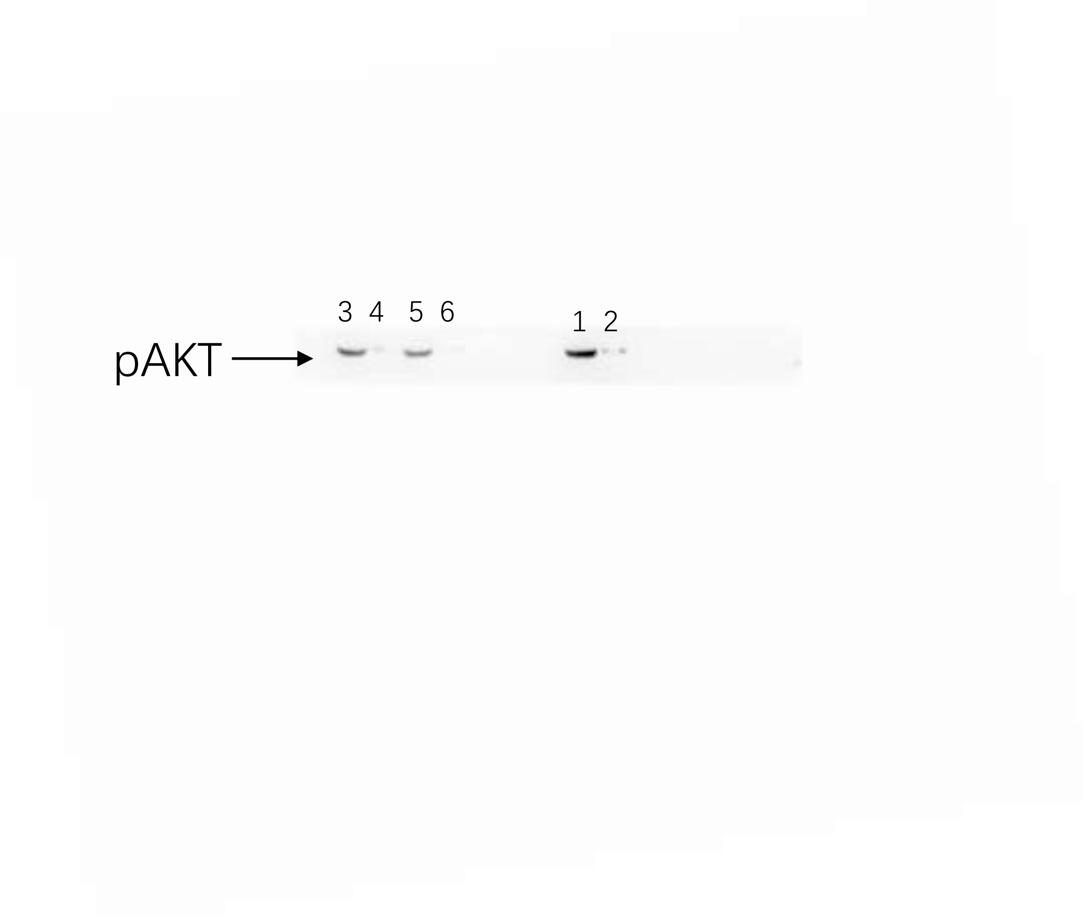

Full and uncropped western blot for Figure 6D Lanes 7-10 are on the figure

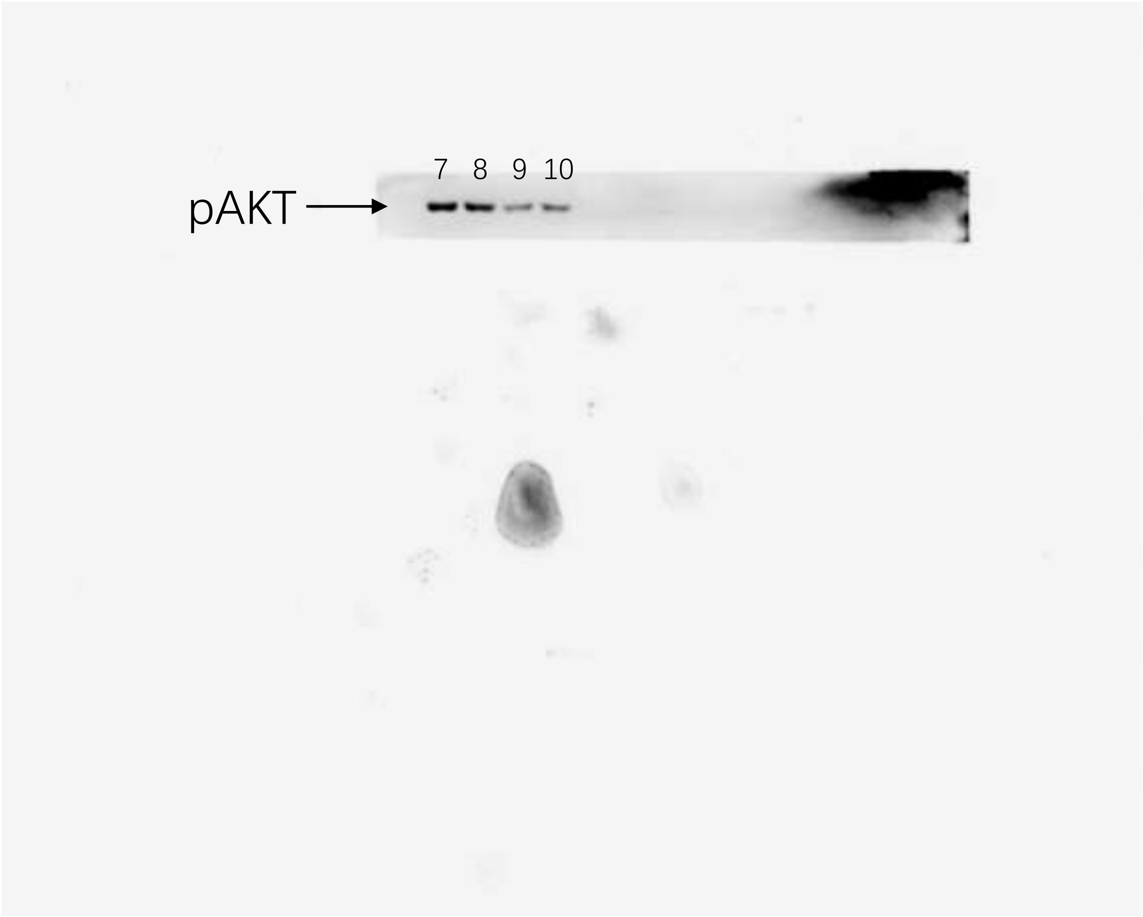

Full and uncropped western blot for Figure 6D Lanes 11-14 are on the figure

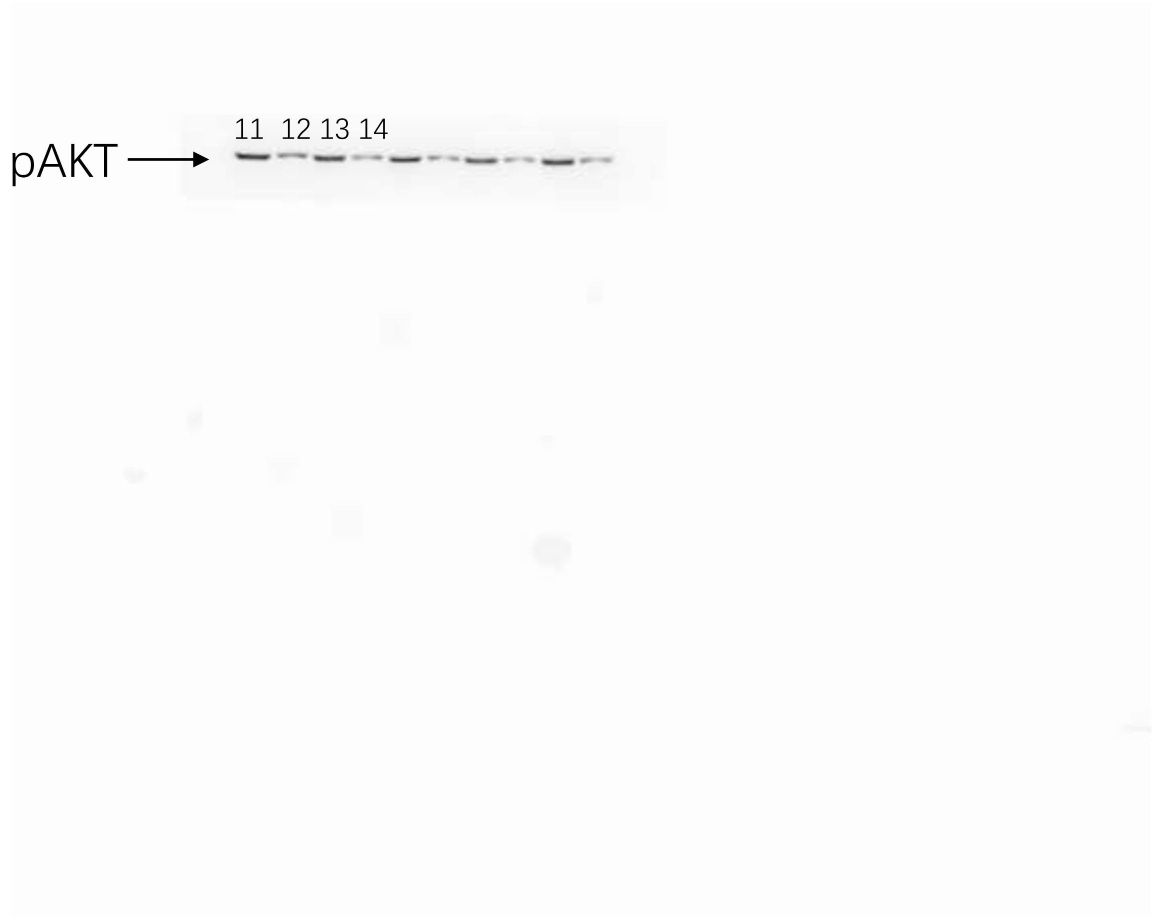

Full and uncropped western blot for Figure 6D Lanes 15-18 are on the figure

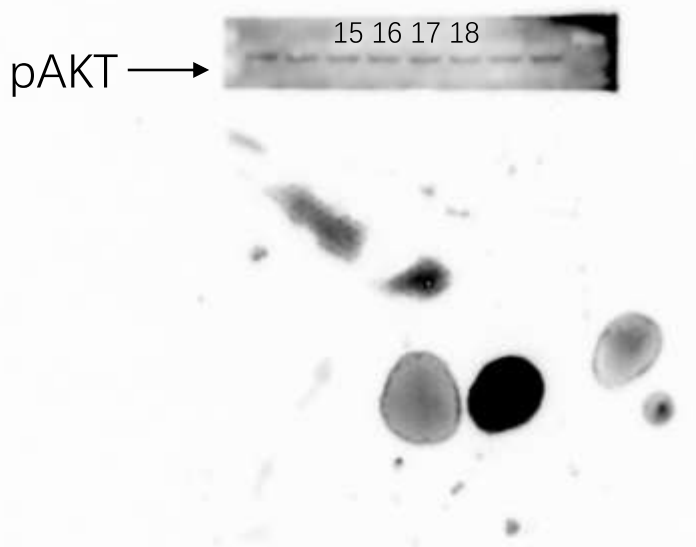

Full and uncropped western blot for Figure 6D Lanes 1-6 are on the figure

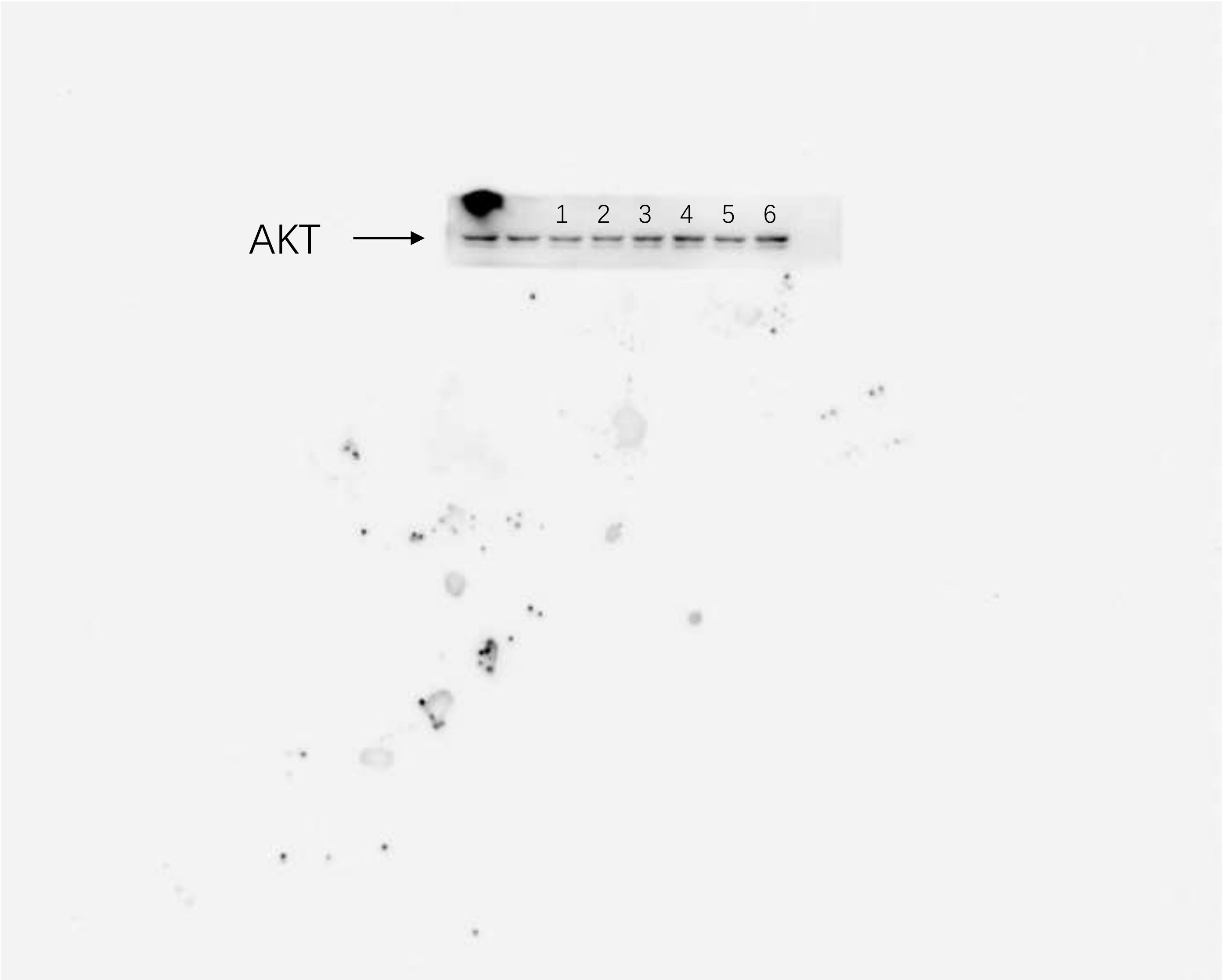

Full and uncropped western blot for Figure 6D Lanes 7-14 are on the figure

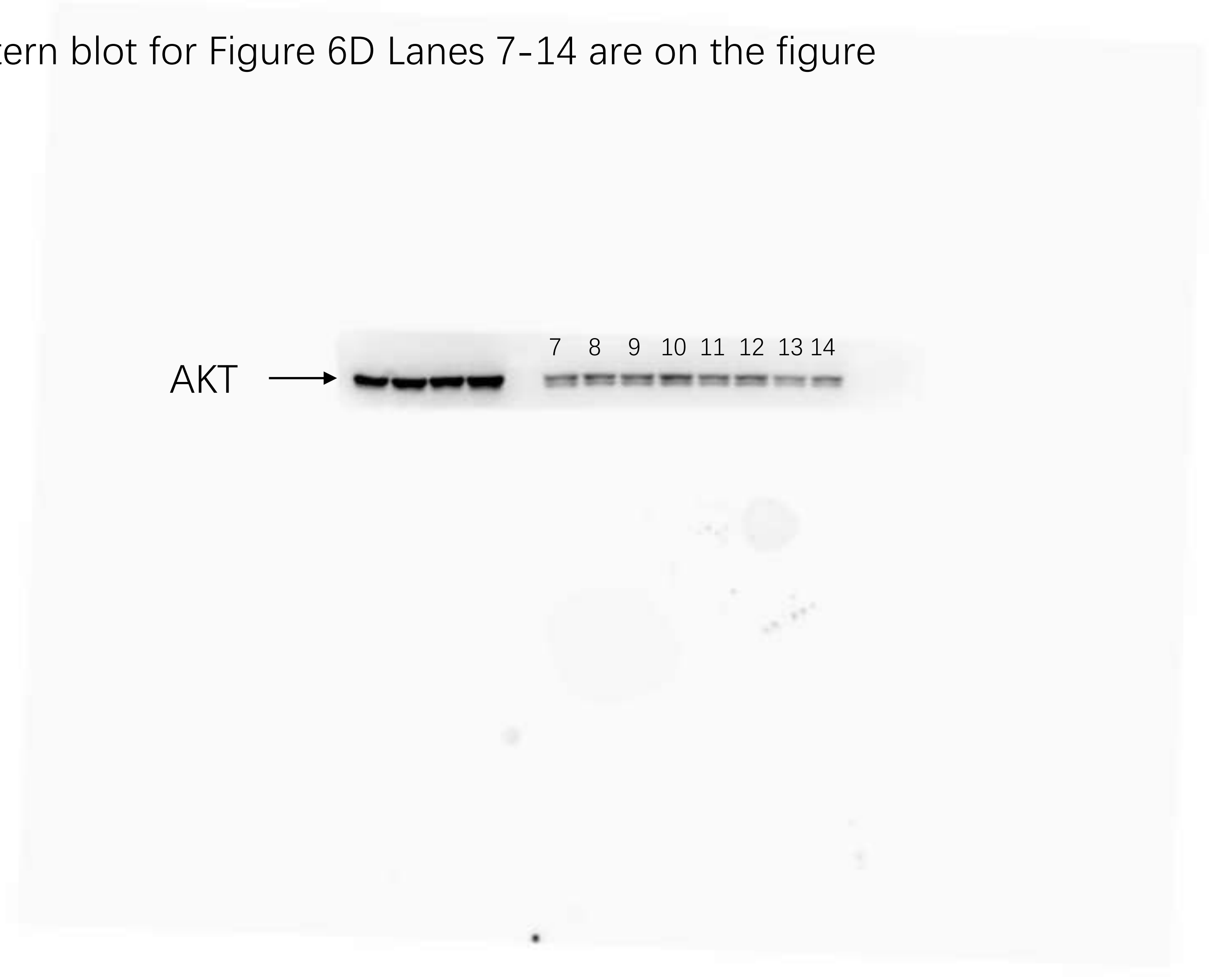

Full and uncropped western blot for Figure 6D Lanes 15-18 are on the figure

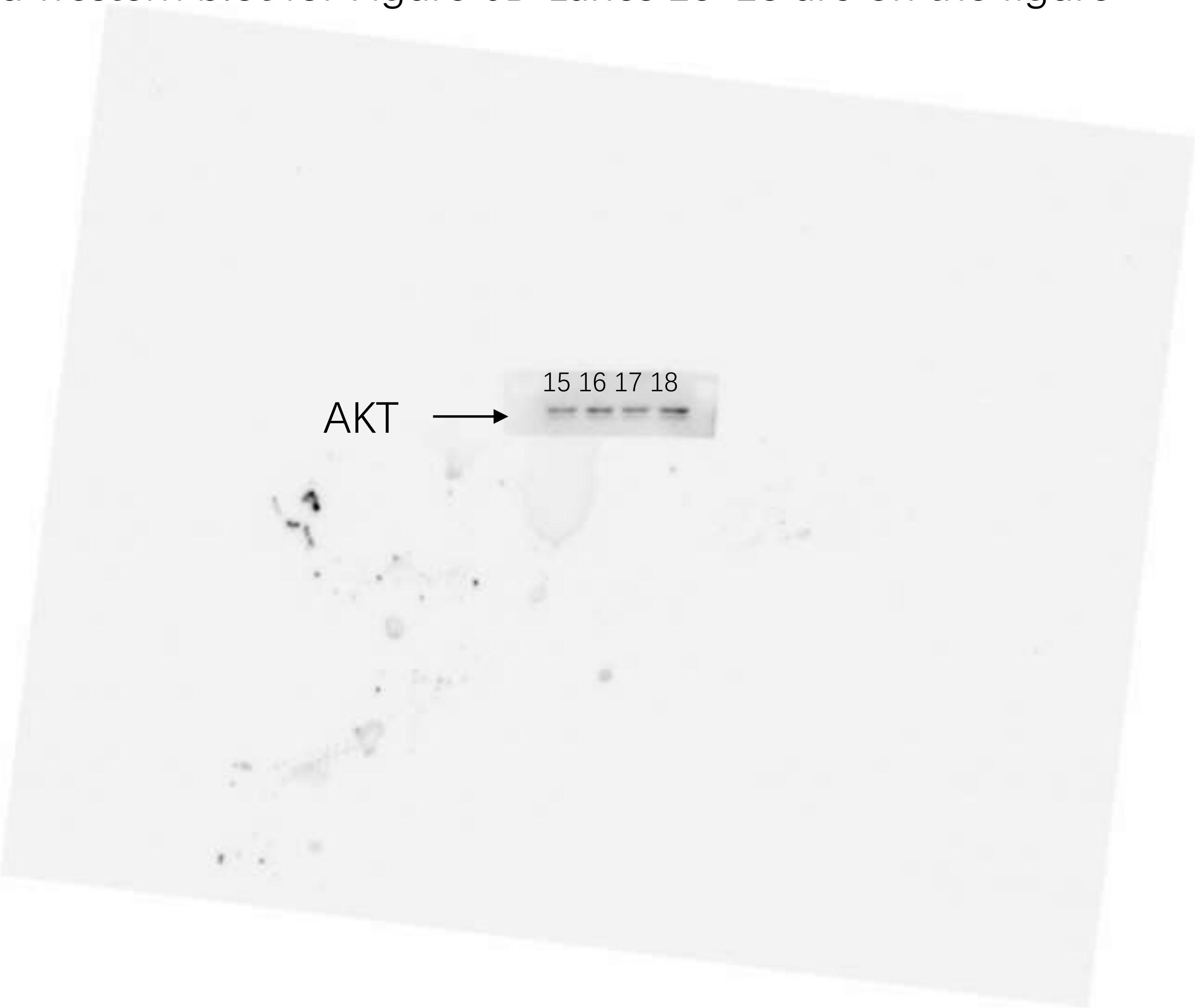

Full and uncropped western blot for Figure 6D Lanes 15-18 are on the figure

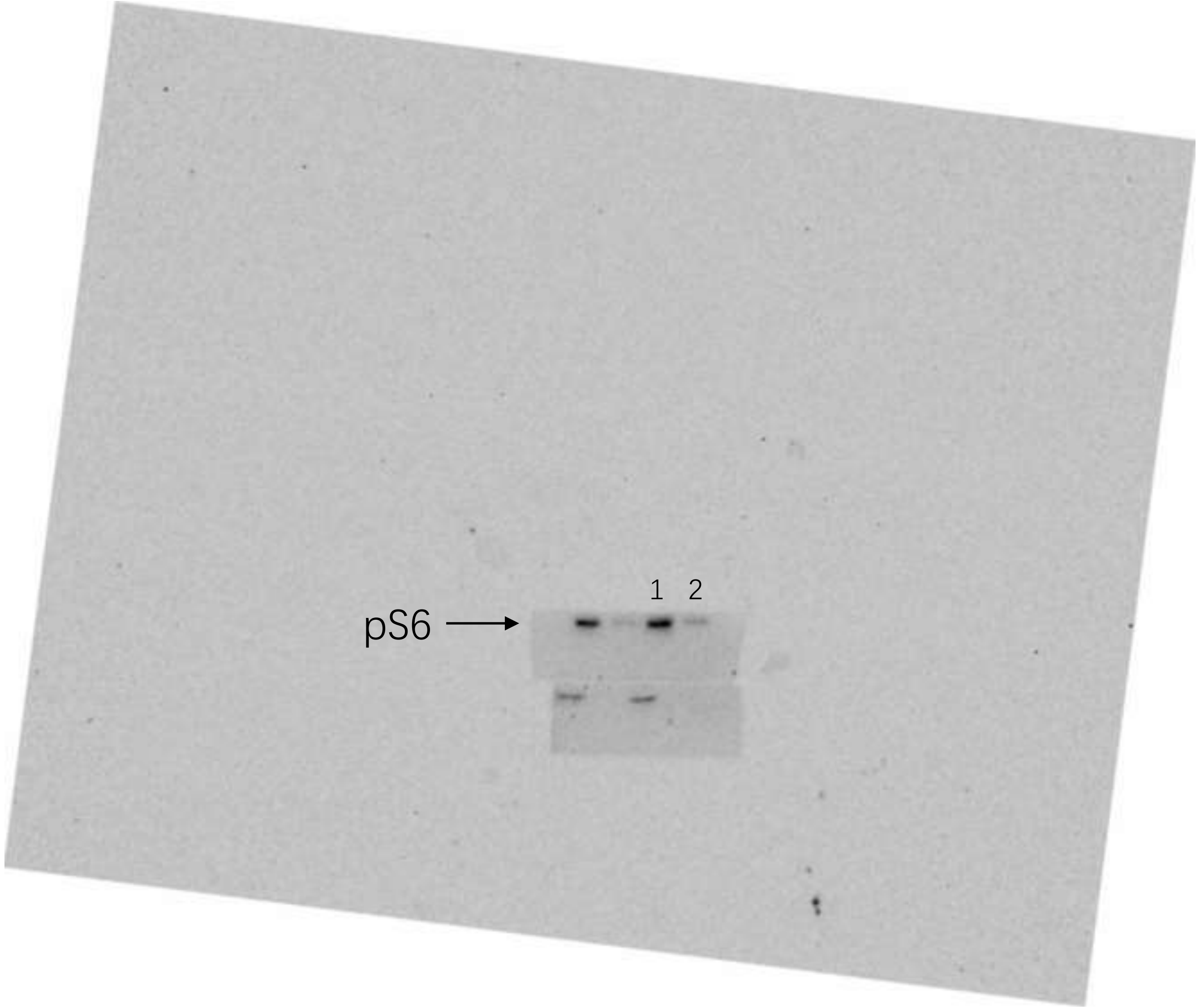

Full and uncropped western blot for Figure 6D Lanes 3-6, 11-14 are on the figure

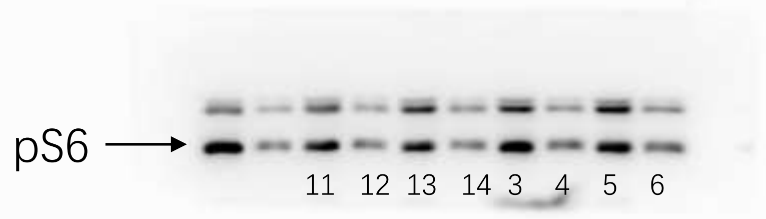

Full and uncropped western blot for Figure 6D Lanes 7-10, 15-18 are on the figure

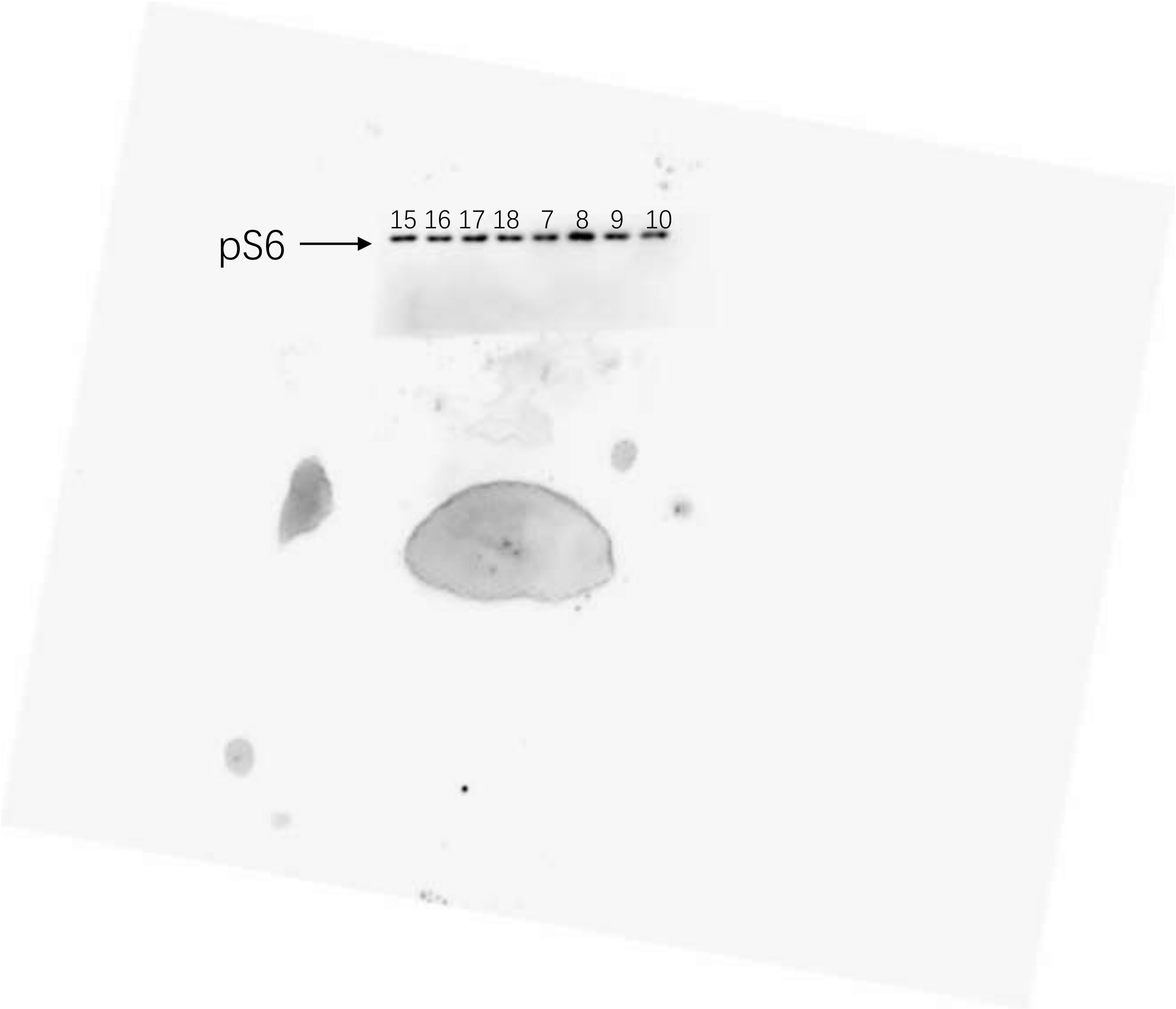

Full and uncropped western blot for Figure 6D Lanes 1-6, 11-14 are on the figure

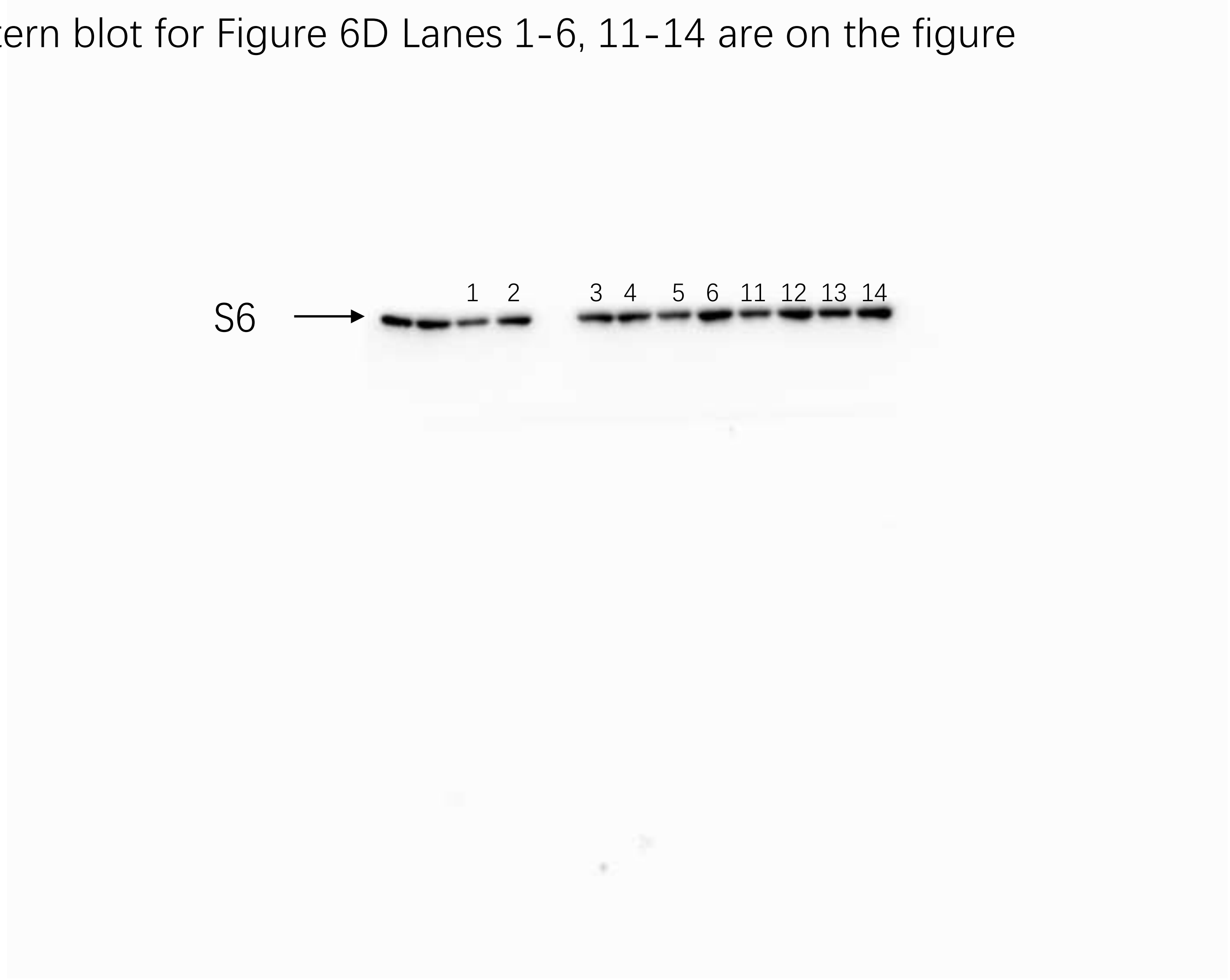

Full and uncropped western blot for Figure 6D Lanes 7-10, 15-18 are on the figure

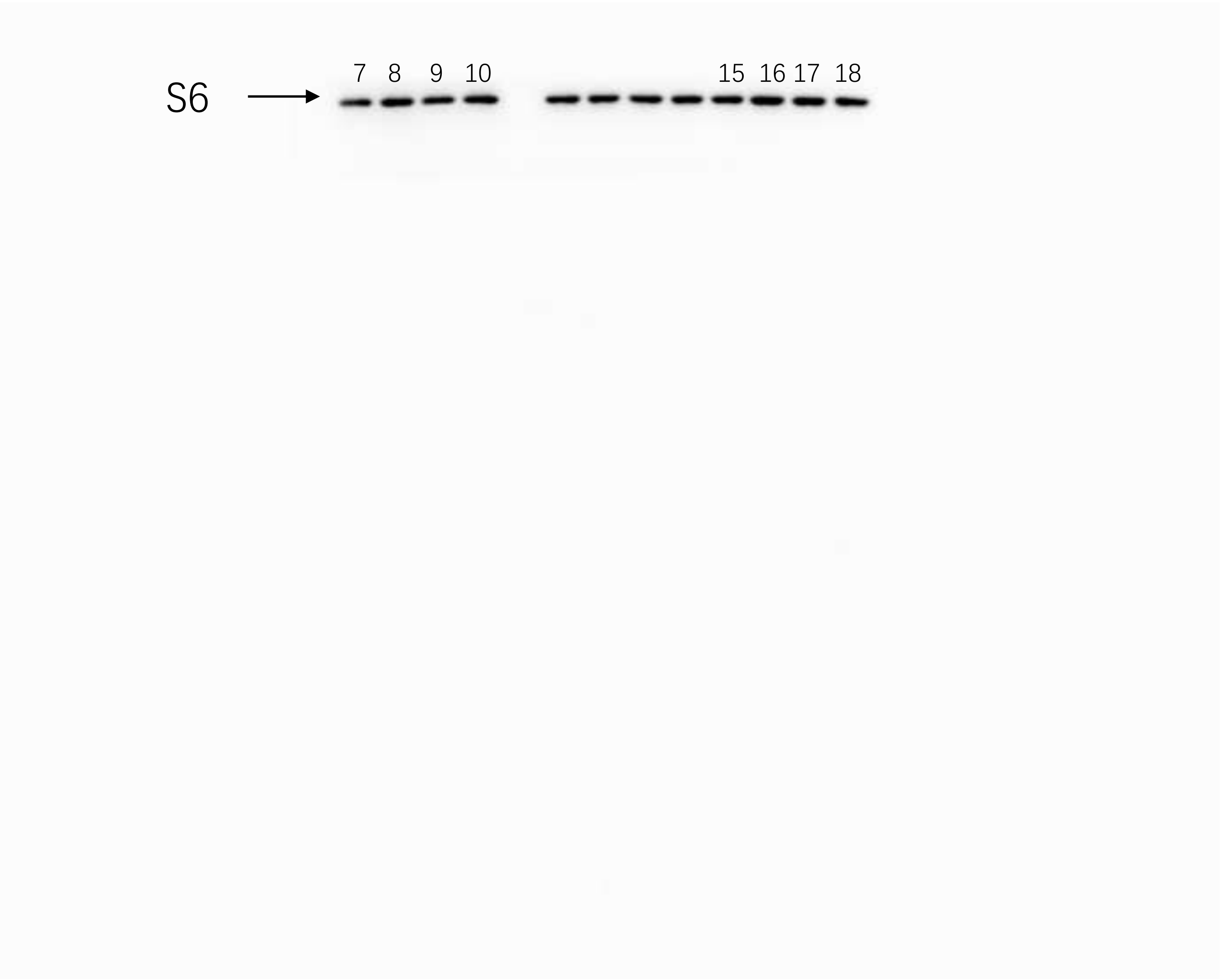

Full and uncropped western blot for Figure S2A Lanes 1-3 are on the figure

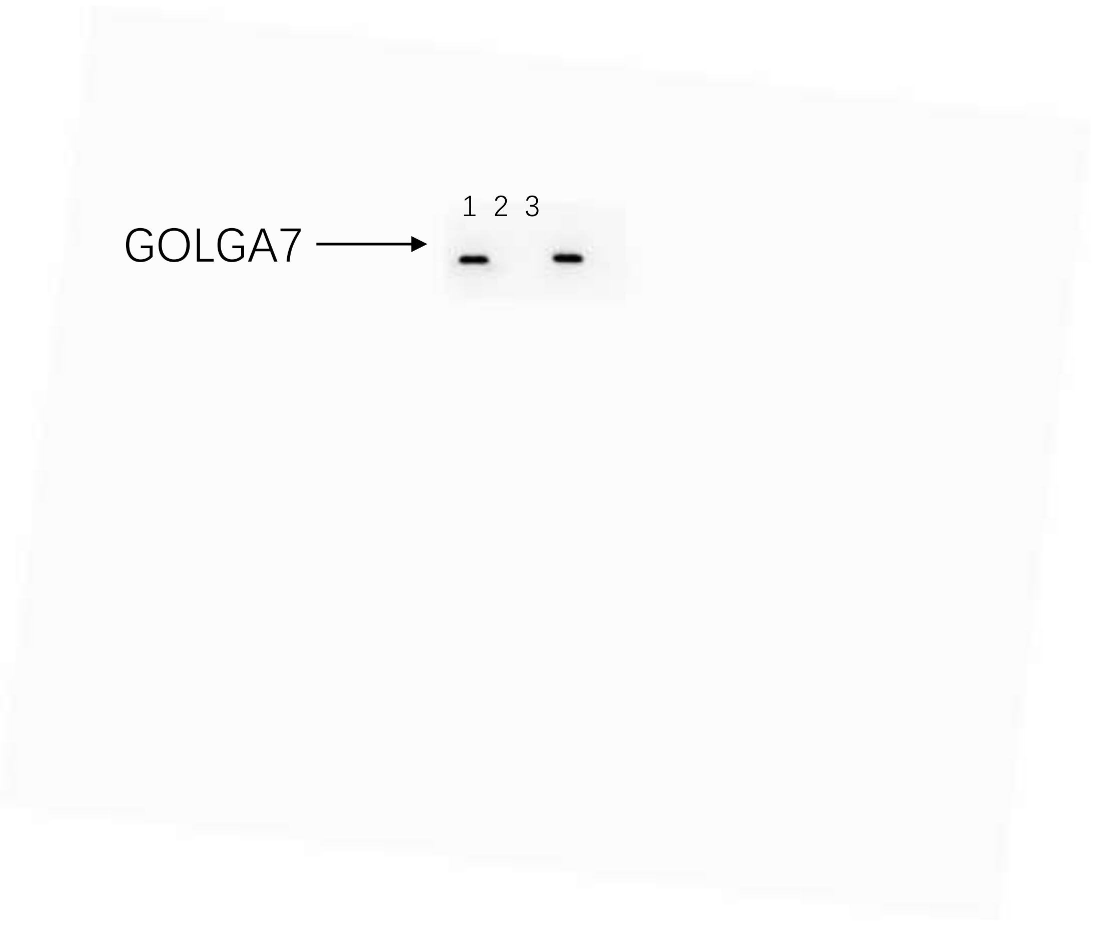

Full and uncropped western blot for Figure S2A Lanes 1-3 are on the figure

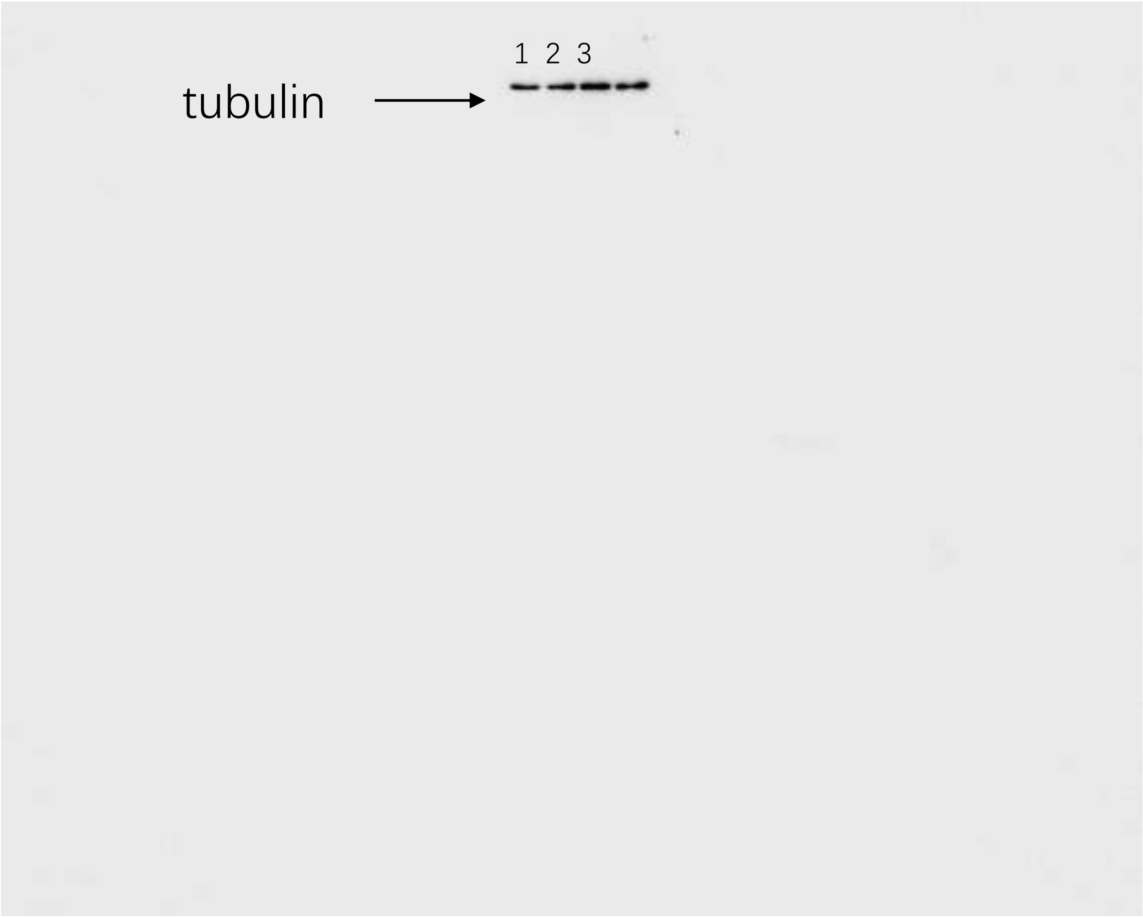

Full and uncropped western blot for Figure S2B Lanes 1-3 are on the figure

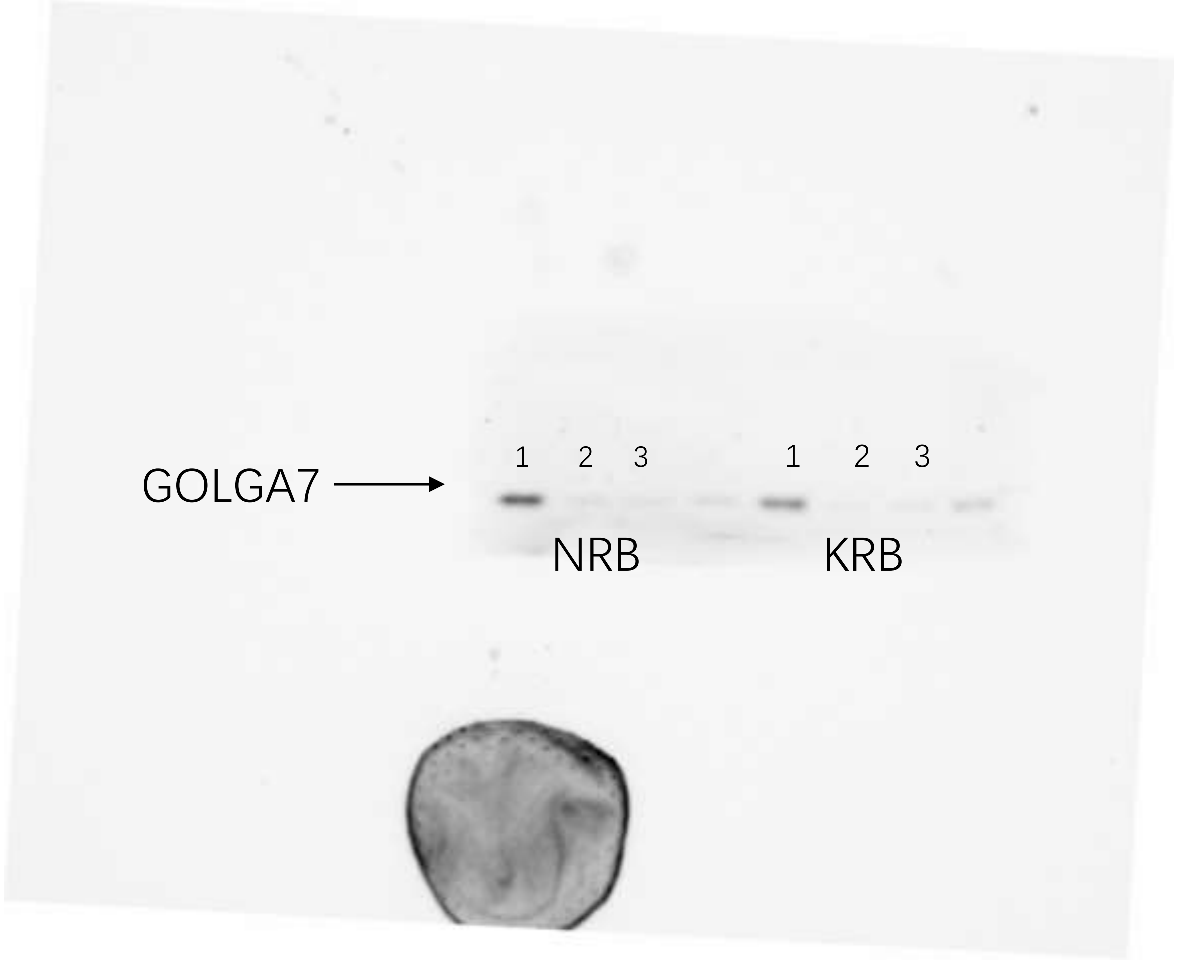

Full and uncropped western blot for Figure S2B Lanes 1-3 are on the figure

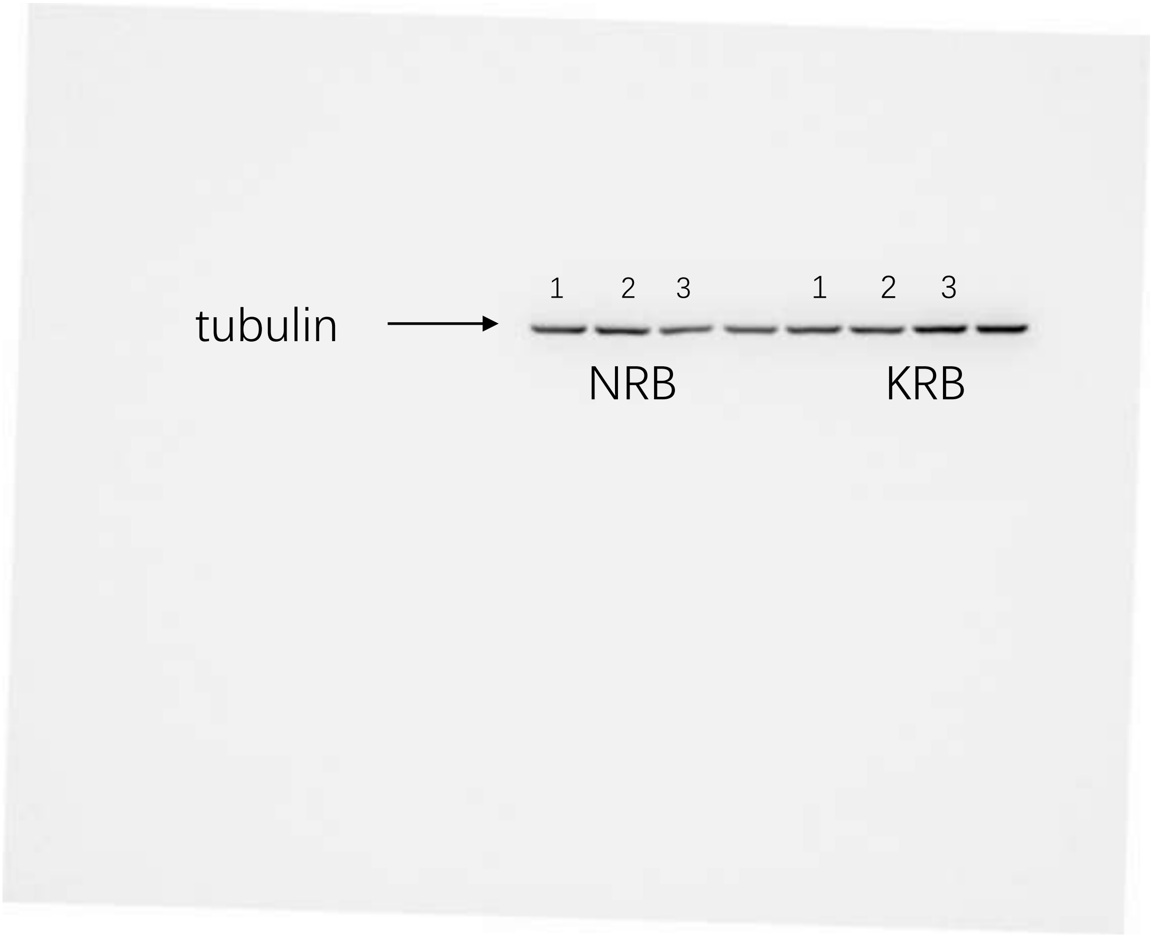

Full and uncropped western blot for Figure S5 Lanes 1-4 are on the figure

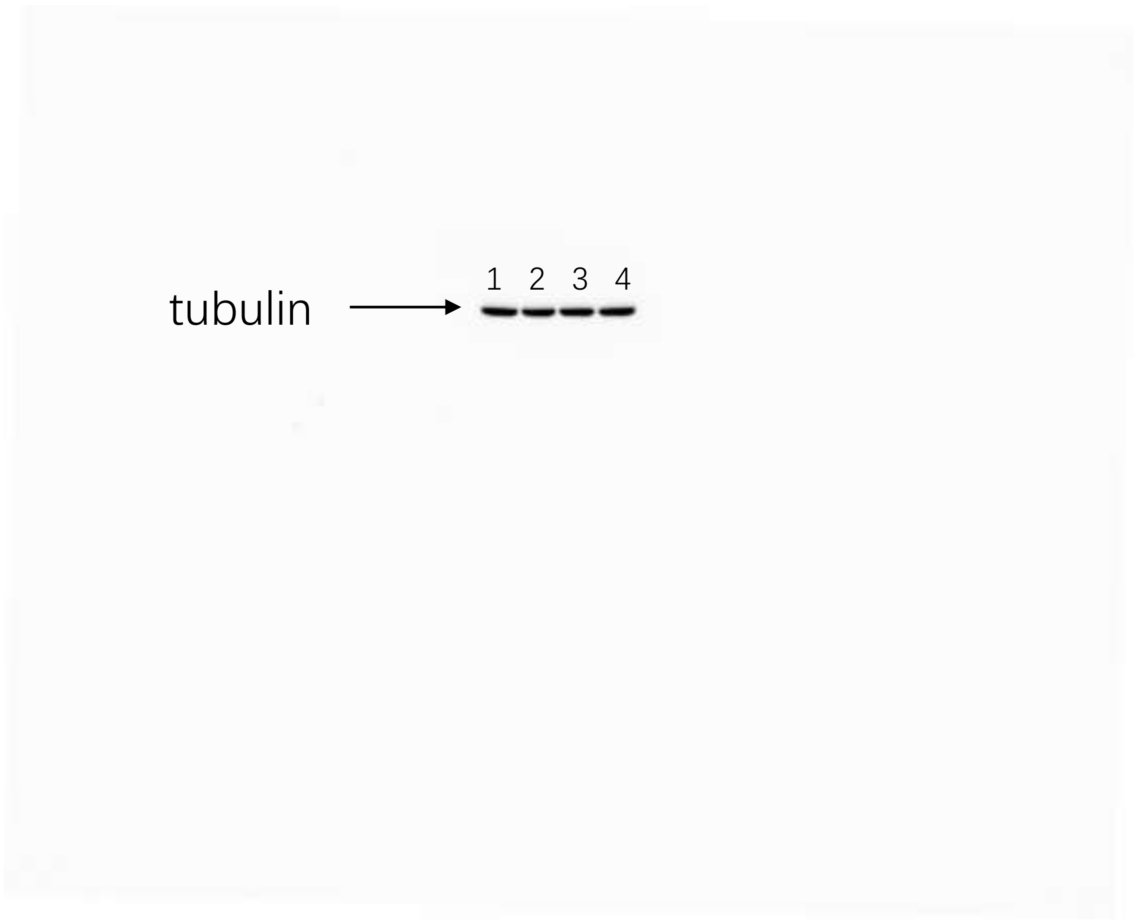

Full and uncropped western blot for Figure S5 Lanes 1-4 are on the figure

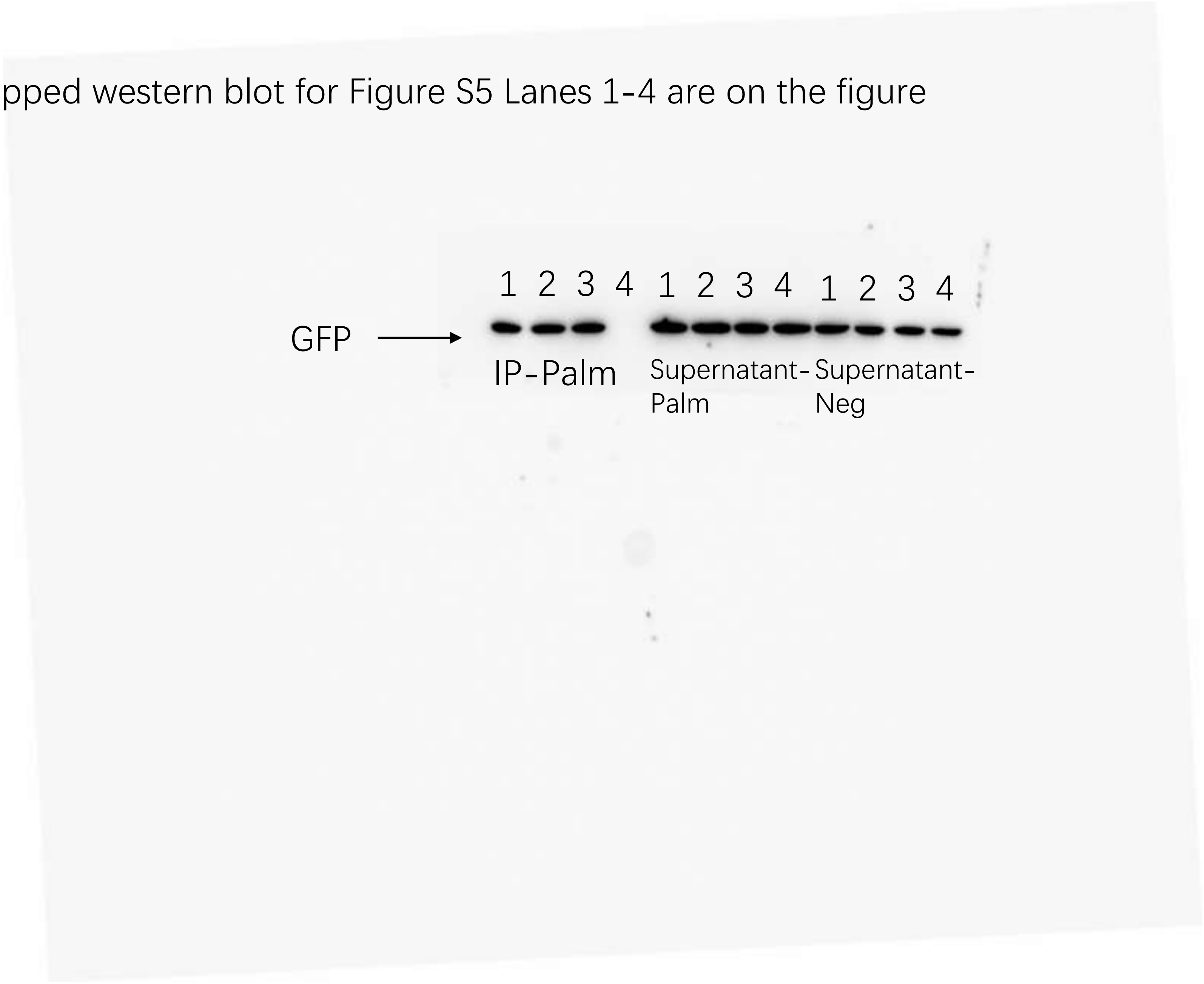

Full and uncropped western blot for Figure S5 Lanes 1-4 are on the figure

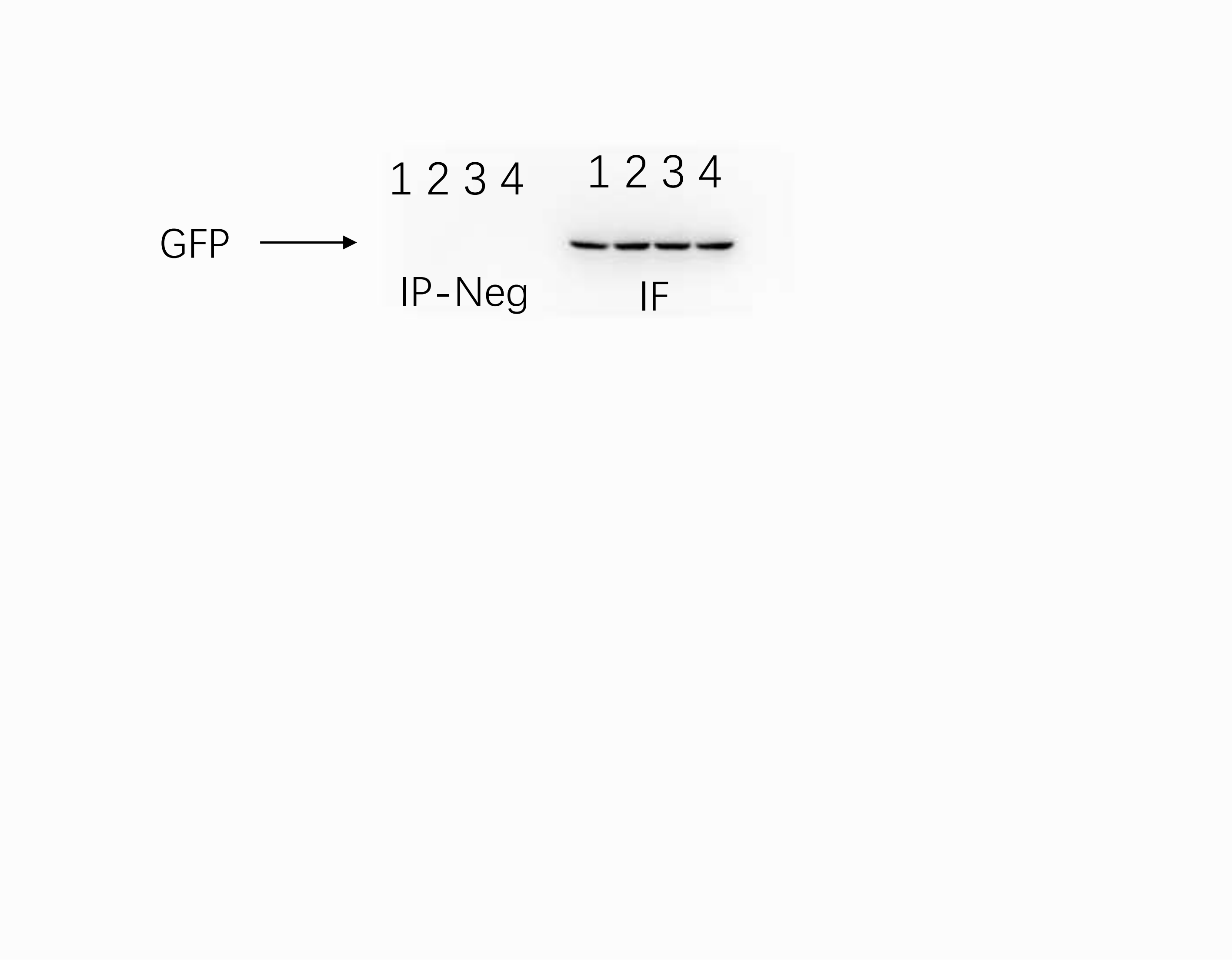

Supplement: Supplementary file 1 — Additional file 1. [file 12964_2024_1498_MOESM1_ESM.pdf]
