# Supplementary material for: GOLGA7 is essential for NRAS trafficking from the Golgi to the plasma membrane but not for its palmitoylation
Source: Cell Commun Signal. 2024 Feb 5;22:98. doi: 10.1186/s12964-024-01498-w (PMC10845536; doi:10.1186/s12964-024-01498-w)
Supplement: Supplementary file 2 — Additional file 2. [file 12964_2024_1498_MOESM2_ESM.docx]

**Supplementary Figures**

**
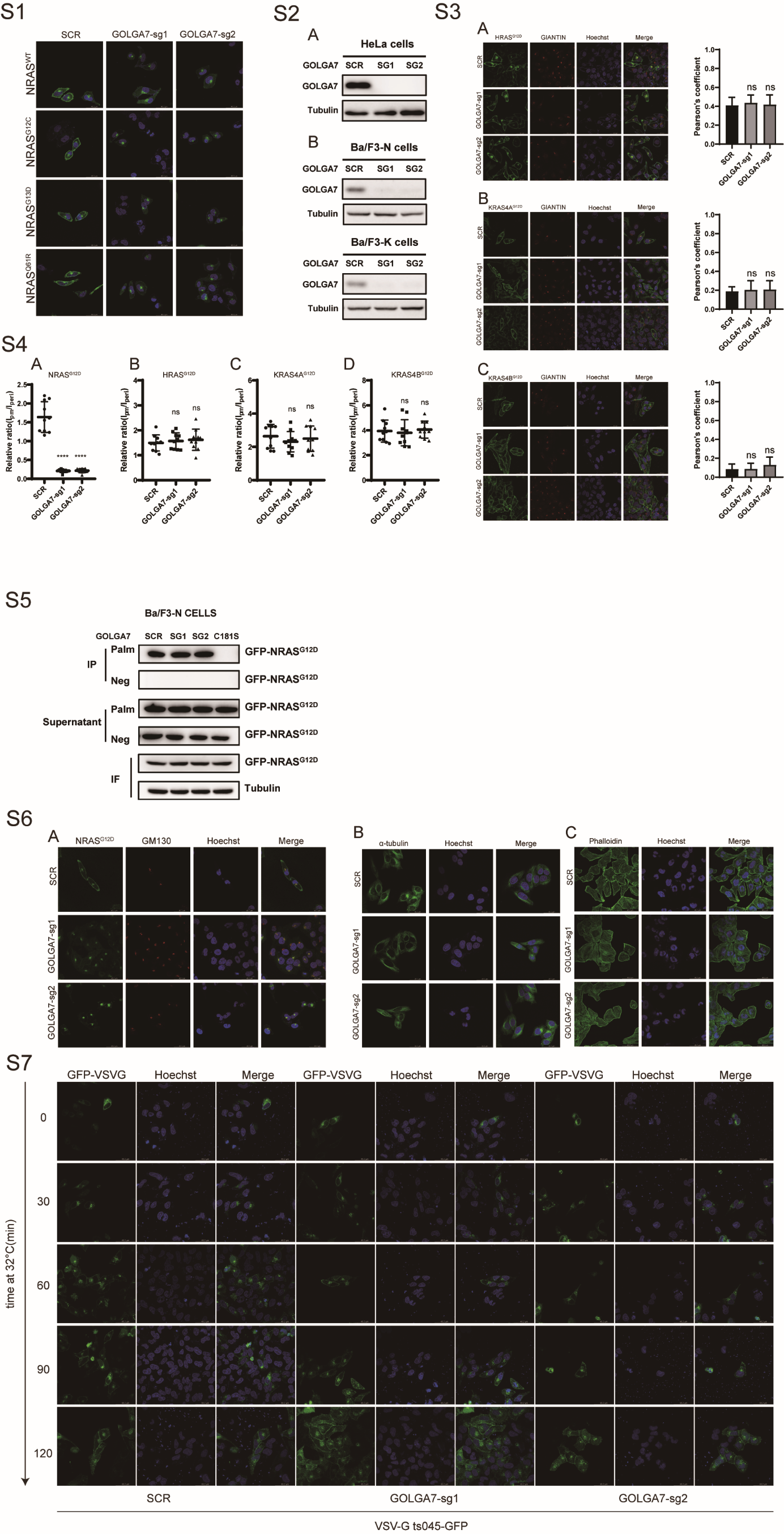
**

**
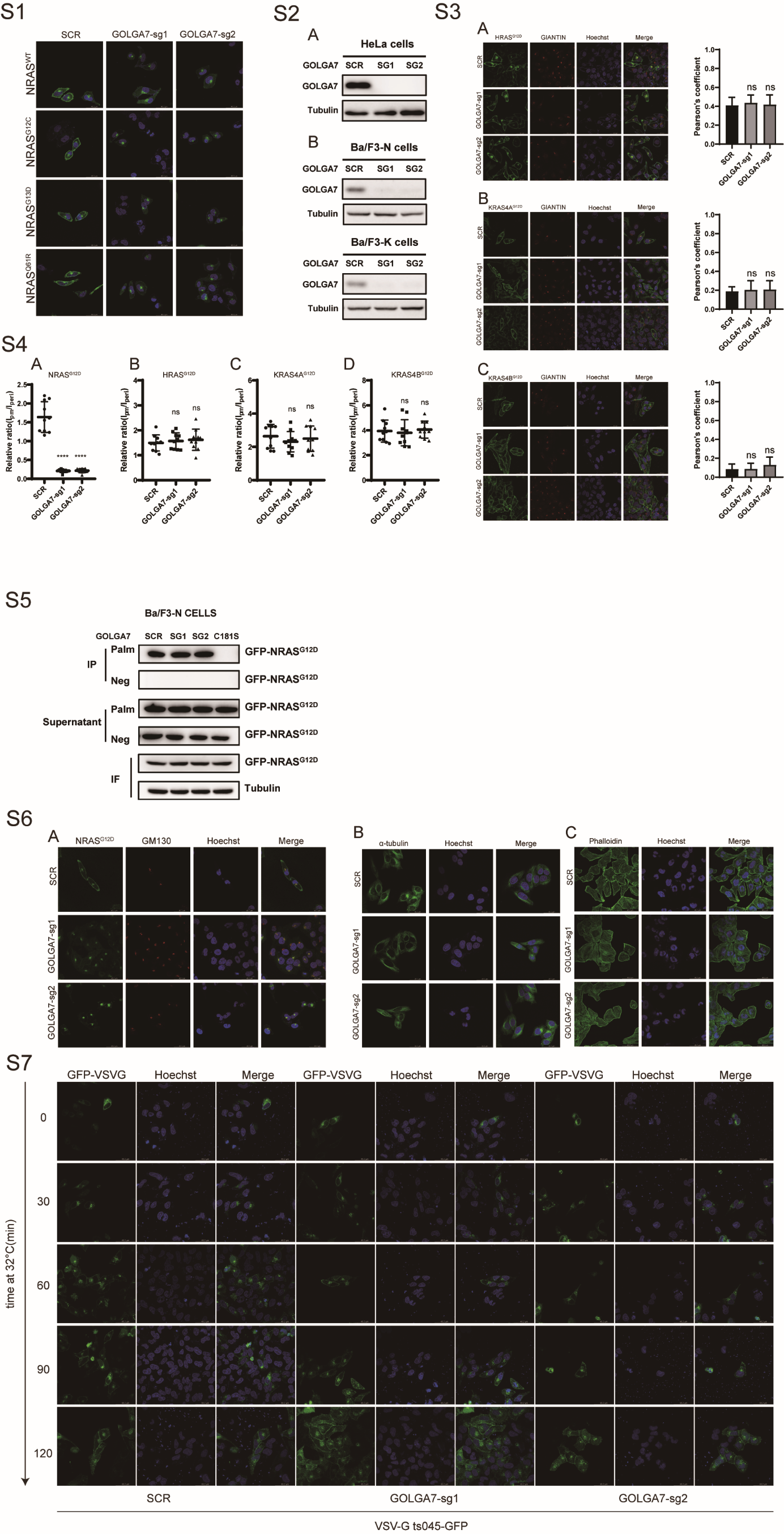
**

(S1) GFP-NRAS^WT/G12C/G13D/Q61R^ were visualized respectively by confocal microscopy in HeLa cells ± sgGOLGA7.

(S2 A) Lysates from HeLa cells ± sgGOLGA7 were immunoblotted using the indicated antibodies.

(S2 B) Lysates from Ba/F3-N and Ba/F3-K cells ± sgGOLGA7 were immunoblotted using the indicated antibodies.

(S3, left panel) Immunofluorescence showing the colocalization of GFP-HRAS^G12D^/GFP-KRAS4A^G12D^/GFP-KRAS4B^G12D^ and GIANTIN in HeLa cells ± sgGOLGA7. Scale bars, 42.6 µm.

(S3, right panel) Statistical quantification of the colocalizations (Pearson’s coefficient) of GFP-HRAS^G12D^/GFP-KRAS4A^G12D^/GFP-KRAS4B^G12D^ and GIANTIN. Values are the means ± SD from n = 15 per group, compared using two- tailed Student’s t test. GFP-HRAS^G12D^ GOLGA7-sg1, ns p =0.4195; GFP-HRAS^G12D^ GOLGA7-sg2, ns p =0.8342; GFP-KRAS4A^G12D^ GOLGA7-sg1, ns p =0.5926; GFP-KRAS4A^G12D^ GOLGA7-sg2, ns p =0.4994; GFP—KRAS4B^G12D^ GOLGA7-sg1, ns p =0.9265; GFP—KRAS4B^G12D^ GOLGA7-sg2, ns p =0.0886.

(S4) Relative fluorescence intensity ratio of the plasma membrane (I_pm_) versus average perinuclear region (I_peri_). Values are the means ± SD from n = 10 per group, compared using two- tailed Student’s t test. GFP-NRAS^G12D^ GOLGA7-sg1, **** p <0.0001; GFP-NRAS^G12D^ GOLGA7-sg2, **** p <0.0001; GFP-HRAS^G12D^ GOLGA7-sg1, ns p =0.5997; GFP-HRAS^G12D^ GOLGA7-sg2, ns p =0.4589; GFP-KRAS4A^G12D^ GOLGA7-sg1, ns p =0.2784; GFP-KRAS4A^G12D^ GOLGA7-sg2, ns p =0.6649; GFP—KRAS4B^G12D^ GOLGA7-sg1, ns p =0.7601; GFP—KRAS4B^G12D^ GOLGA7-sg2, ns p =0.7359.

(S5) Acyl-RAC assays of Ba/F3-N cells ± sgGOLGA7 transfected with GFP-NRAS^G12D^ or Ba/F3-N cells transfected with GFP-NRAS^G12D, C181S^. Palmitoylation of GFP-NRAS^G12D, C181S^ mutant is undetectable, while GFP-NRAS^G12D^ in Ba/F3-N cells ± sgGOLGA7 are all shown to be palmitoylated (Palm in IP). The preserved fraction of immunoprecipitants (Neg in IP) show the specificity of the assay. The cleaved fraction (Palm in Supernatant) and preserved fraction of supernatants (Neg in Supernatant) represent non-palmitoylated proteins and the total protein unbound to the resin, respectively.

(S6 A) Immunofluorescence showing the colocalization of GFP-NRAS^G12D^ and GM130 (Golgi) in HeLa cells ± sgGOLGA7. Scale bars, 42.6 µm.

(S6 B-C) Immunofluorescence showing the α-tubulin(B) and phalloidin/F-actin(C) in HeLa cells ± sgGOLGA7. Scale bars, 42.6 µm.

(S7) HeLa cells± sgGOLGA7 expressing ts045-VSVG-GFP were incubated at 40°C for 20h. The cells were then shifted to 32°C for 0 min, 30 min, 60 min, 90 min, or 120 min. Subsequently, the cells were fixed, permeabilized, and stained with Hoechst33342. Scale bars, 42.6 µm.
