# Supplementary material for: GOLGA7 is essential for NRAS trafficking from the Golgi to the plasma membrane but not for its palmitoylation
Source: Cell Commun Signal. 2024 Feb 5;22:98. doi: 10.1186/s12964-024-01498-w (PMC10845536; doi:10.1186/s12964-024-01498-w)
Supplement: Supplementary file 3 — Additional file 3. [file 12964_2024_1498_MOESM3_ESM.docx]

**Supplementary Table 1: List of antibodies used in this study.**

| **Antibody** | **Vendor** | **Catalogue Number** |
| --- | --- | --- |
| Na^+^K^+^ATPase | Abcam | ab76020 |
| GFP | Cell Signaling Technology | 2555S |
| GAPDH | Proteintech | HRP-60004 |
| GOLGA7 | ABclonal | Customer Antibody  Project number: WG-04524 |
| HA | Cell Signaling Technology | 3724 |
| ERK | Cell Signaling Technology | 4695 |
| Phospho-ERK | Cell Signaling Technology | 4370 |
| AKT | Cell Signaling Technology | 4691 |
| Phospho-AKT (Ser473) | Cell Signaling Technology | 4060 |
| S6 | Cell Signaling Technology | 2317 |
| Phospho-S6 | Cell Signaling Technology | 4858 |
| RCAS1 | Cell Signaling Technology | 12290P |
| Transferrin Receptor | Abcam | ab38171 |
| GIANTIN | Abcam | ab37266 |
| GM130 | Abcam | ab52649 |
| TGN46 | Thermo | MA3-063 |
| α-Tubulin | Proteintech | HRP-66031 |
| β-actin | Proteintech | HRP-60008 |
| Anti-rabbit HRP | Cell Signaling Technology | 7074 |
| Goat anti-Rabbit Alexa Fluor™ 488 | Abcam | ab150077 |
| Goat anti-Rabbit Alexa Fluor™ 555 | Abcam | ab150078 |
| Hoechst 33342 | Beyotime | C1025 |
